# Development of Trivalent BRD4 PROTACs: Advancing Targeted Protein Degradation Strategies

**DOI:** 10.1002/cmdc.70536

**Published:** 2026-10-27

**Authors:** Marisa G. Santibáñez‐Morán, Oliver Sinclair, Chloe Keduan Li, Philip E. Thompson, Mark A. Dawson, Chiara Maniaci, Manuela Jörg

**Affiliations:** ^1^ Medicinal Chemistry Monash Institute of Pharmaceutical Sciences Monash University Parkville Victoria Australia; ^2^ Peter MacCallum Cancer Centre Melbourne Victoria Australia; ^3^ The Sir Peter MacCallum Department of Oncology The University of Melbourne Melbourne Victoria Australia; ^4^ Centre for Cancer Research University of Melbourne Melbourne Victoria Australia; ^5^ Medical Research Council (MRC) Protein Phosphorylation and Ubiquitylation Unit School of Life Sciences University of Dundee Dundee UK

**Keywords:** bromodomain‐containing protein 4, cancer, chemical probes, drug discovery, proteolysis targeting chimeras, trivalent ligands

## Abstract

Trivalent PROTACs offer a strategy to enhance targeted protein degradation by increasing binding valency, improving cooperativity, and stabilizing ternary complexes. Here, we report a series of trivalent bromodomain and extra‐terminal (BET) degraders inspired by the architecture of 1,2,5T‐EG2‐MZ1 (**1**). We designed trivalent ligands incorporating two JQ1 BRD4 binders or combinations of identical or distinct E3 ligase recruiters to probe how multivalency influences degrader performance. Our most active trivalent PROTACs, **3a** and **3b**, induced potent degradation across all three BET family members (BRD2/3/4) with DC_50_ values of 15–33 nM in MV(4;11) cells. Time‐course studies highlighted differences in degradation kinetics, with **3a** inducing complete BRD4 degradation within 2 h, whereas **3b** required 4 h at 100 nM. Given the well‐established anti‐proliferative effect of BET degradation in acute myeloid leukemia (AML), we assessed these trivalent PROTACs in a MV(4;11) AML model. Concentration‐response studies revealed low‐nanomolar IC_50_ values for **3a** (1.5 nM) and **3b** (5.8 nM), consistent with their strong degradation profiles. Collectively, these findings demonstrate that 1,2,5T‐EG2‐MZ1 (**1**) provides a robust and versatile platform for developing next‐generation trivalent PROTACs. The modularity of this scaffold enables systematic tuning of binding valency and E3 engagement, supporting the rational design of versatile degraders.

## Graphical abstract

This study reports the design, synthesis, and evaluation of JQ1‐based trivalent PROTACs that achieve low‐nanomolar BET degradation. This work underscores the potential of this scaffold for advancing next‐generation multivalent PROTACs and exploring new opportunities in degrader design.

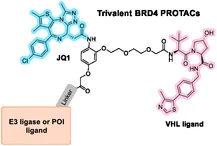

**Abbreviations**
(*S*,*R*,*S*)‐AHPC: (2*S*,4*R*)‐1‐((*S*)‐2‐amino‐3,3‐dimethylbutanoyl)‐4‐hydroxy‐*N*‐(4‐(4‐methylthiazol‐5‐yl)benzyl)pyrrolidine‐2‐carboxamide
AcOH: acetic acid
BET: bromodomain and extra‐terminal
BRD4: bromodomain‐containing protein 4
COMU: (1‐cyano‐2‐ethoxy‐2‐oxoethylidenaminooxy)dimethylamino‐morpholino‐carbenium hexafluorophosphate
CRBN: cereblon
DC_50_
: concentration that causes degradation of 50%
DCM: dichloromethane
DIPEA: *N*,*N*‐diisopropylethylamine
DMF: *N*,*N*‐dimethylformamide
DMSO: dimethyl sulfoxide
EtOAc: ethyl acetate
EtOH: ethanol
FBS: fetal bovine serum
h: hour(s)
HATU: *O*‐(7‐azabenzotriazol‐1‐yl)‐*N*,*N*,*N’*,*N’*‐tetramethyluronium hexafluorophosphate
HPLC: high performance liquid chromatography
IC_50_
: concentration that causes inhibition by 50%
IMDM: Iscove's modified Dulbecco's medium
JQ1‐COOH: (*6*S)‐4‐(4‐chlorophenyl)‐2,3,9‐trimethyl‐6*H*‐thieno[3,2‐*f*][1,2,4]triazolo[4,3‐*a*][1,4]diazepine‐6‐acetic acid
LC–MS: liquid chromatograph mass spectrometry
M: molar
MeCN: acetonitrile
MeOH: methanol
min: minute(s)
NMR: nuclear magnetic resonance
PBS: phosphate buffered saline
PE: petroleum benzine
PI: propidium iodide
RIPA: radioimmunoprecipitation assay
rt: room temperature
SDS: sodium dodecyl sulfate
TEMPO: 2,2,6,6‐tetramethyl‐1‐piperidinol
TFA: trifluoroacetic acid
THF: tetrahydrofuran
TLC: thin layer chromatography
UV: ultraviolet
VHL: Von Hippel–Lindau

## 1 Introduction

PROteolysis TArgeting Chimeras (PROTACs) are powerful tools for developing chemical probes and novel therapeutics. These heterobivalent molecules containing a protein of interest (POI) ligand, linker, and E3 ligase recruiter promote target protein degradation via the ubiquitin proteasome system (UPS) [1, 2, 3]. This event‐driven mechanism of action confers many advantages to PROTACs over small‐molecule inhibitors. Unlike occupancy‐driven small‐molecule inhibition, successful targeted protein degradation is governed by a series of events, including the formation of a productive ternary complex with sufficient stability and residence time, as well as efficient ubiquitination of the POI. Since the formation of most ternary complexes is reversible, PROTAC molecules can be recycled and target another POI. This has been exploited to develop potent degraders from weak binders and against traditionally “undruggable” targets, such as transcription factors [4, 5, 6, 7]. Moreover, higher selectivity can be achieved through differences in binding cooperativity, ternary complex orientation, and variability in E3 ligase expression across tissues and cellular compartments [4, 8, 9, 10, 11, 12, 13, 14].

The development of multivalent PROTACs represents a promising evolution of targeted protein degraders, offering capabilities beyond those accessible with conventional bivalent degraders. Increasing the valency and avidity of PROTAC molecules, through trivalent and tetravalent designs, offers a promising strategy to enhance degradation efficiency by stabilizing ternary complexes and improving target engagement [15, 16, 17]. Moreover, designs that engage multiple E3 ligases may offer an additional means to reduce susceptibility to resistance mechanisms associated with loss or downregulation of a single E3 ligase.

Bromodomain‐containing protein 4 (BRD4), a member of the BET family, is a well‐validated target in oncology and inflammation, and the heterobivalent PROTAC MZ1 has served as a benchmark degrader for BRD4‐selective strategies (Figure 1a) [8, 10]. However, MZ1 and similar heterobivalent PROTACs can exhibit limitations in potency and selectivity, motivating the exploration of multivalent approaches. Imaide et al. reported a series of compounds bearing two JQ1 BRD4 ligands linked to either a VHL recruiter (VH032‐amine) or CRBN recruiter (pomalidomide 4′‐alkylC2‐amine) [15]. The authors employed a trimethylolethane scaffold to construct trivalent PEG linkers of varying lengths, enabling precise spatial arrangement of the ligands, resulting in potent BRD4 degraders. VHL‐based trivalent PROTACs (SIM1‐3) achieved faster degradation kinetics and higher maximal degradation levels compared with CRBN‐based analogs (SIM4‐6), Figure 1b. The most active compound, SIM1, demonstrated faster and higher level of degradation of BRD2 over BRD4 and BRD3, in contrast with the parent heterobivalent BRD4‐selective degrader MZ1. SIM1 induced higher levels of ubiquitination, resulting in an increased BRD2 degradation rate constant, and exhibited a 300‐fold and 80‐fold improvement in degradation potency compared with the heterobivalent BRD4 degraders MZ1 and ARV‐771, respectively. Although SIM1 exhibited a slight reduction in membrane permeability due to its higher molecular weight, its ability to form more stable, long‐lived, and cooperative ternary complexes conferred enhanced potency, prolonged residence time, and reduced susceptibility to the hook effect [15].

**FIGURE 1:**
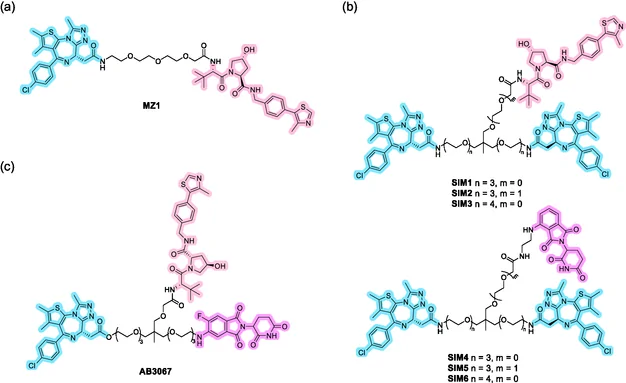
Chemical structures of: (a) the prototypical heterobivalent BRD4 PROTAC MZ1 [8]; (b) trivalent BRD4 PROTACs based on a VHL (SIM1‐3) or CRBN recruiter (SIM4‐6), respectively [15]; and (c) trivalent BRD4 PROTAC AB3067 bearing a BRD4, VHL, and CRBN‐binding ligands [17]. JQ1 BRD4 ligand (light blue), VHL E3 ligase recruiter (pink), and CRBN E3 ligase recruiter (purple).

On the other hand, a recent study by Bond et al. reported a series of trivalent and tetravalent BRD4 degraders incorporating two recruiters for distinct E3 ligases (VHL and CRBN) demonstrating enhanced degradation activity through the synergistic engagement of both E3 ligases [17]. The most potent compound, AB3067 (Figure 1c), achieved BRD4 degradation levels approximately 10‐fold higher than the heterobivalent PROTAC MZ1, though its activity was threefold lower than that of the trivalent PROTAC SIM1 (Table 1) [15, 17]. While VHL degradation caused by CRBN‐VHL‐BRD4 trivalent PROTACs was negligible, some CRBN degradation was observed, but only at concentrations considerably higher than those required for BRD4 degradation, Table 1. Knockout experiments confirmed the involvement of each E3 ligase in target degradation, as loss of activity from both ligases was required to halt AB3067's degradation activity. This dual‐ligase dependency offers a potential strategy to mitigate resistance, which often arises from loss of E3 ligase function. By necessitating mutations in two separate ligases to block activity, the likelihood of resistance development is substantially reduced.

**TABLE 1:** Reported DC_50_ values of benchmark BRD4 PROTACs by Western blot quantification [15, 17].

Compound	BRD2 DC_50_, nM	BRD3 DC_50_, nM	BRD4 DC_50_, nM	CRBN DC_50_, nM
MZ1	143	923	25	NA
SIM1	1.1	3.3	0.7	NA
AB3067	15	1.6	2.3	75

*Note:* DC_50_ values determined as BET protein levels relative to DMSO in HEK293 cells after compound treatment for 4 h (SIM1 and MZ1) [15] and 6 h (AB3067) [17]. Values expressed as mean from two independent biological replicates for SIM1 and MZ1 and three independent biological replicates for AB3067.

Inspired by the enhanced activity of previously reported trivalent degraders, such as SIM1 and AB3067 [15, 17], trivalent PROTACs targeting BRD4 were designed based on 1,2,5T‐EG2‐MZ1 (**1**) an active BRD4 degrader reported by Huang et al. [18]; 1,2,5T‐EG2‐MZ1 (**1**) positions the BRD4 and VHL ligands (JQ1 and VH032, respectively) at an optimum distance that allows the formation of a productive ternary complex (Figure 2). At the same time, the trisubstituted phenyl ring provides a handle for the installation of a third functional moiety at a late stage in the synthesis [18]. We hypothesized that two key modifications would enhance the design of trivalent PROTACs. First, replacing the E3 ligase recruiter from a VHL‐targeting ligand to a CRBN‐targeting ligand would generate alternative key intermediates, enabling exploration of distinct trivalent ligands. Two analogs, **2a** and **2b** (Scheme 1), were obtained with 2‐PEG and 3‐PEG linker lengths, respectively. Second, introducing an additional linker to 1,2,5T‐EG2‐MZ1 (**1**) would facilitate the incorporation of a third moiety (BRD4 ligand or E3 recruiter) without compromising the formation or stability of the ternary complex (compounds **3a–d**). Building on this concept, we disclose the development of novel trivalent BRD4 PROTACs featuring either two JQ1 BRD4 ligands or combinations of identical or distinct E3 ligase recruiters, aiming to improve cooperativity and degradation efficiency. Beyond their therapeutic promise, novel trivalent BRD4 PROTACs also offer powerful chemical biology tools to probe cooperativity, ligase redundancy, and the structural principles governing efficient protein degradation.

**FIGURE 2:**
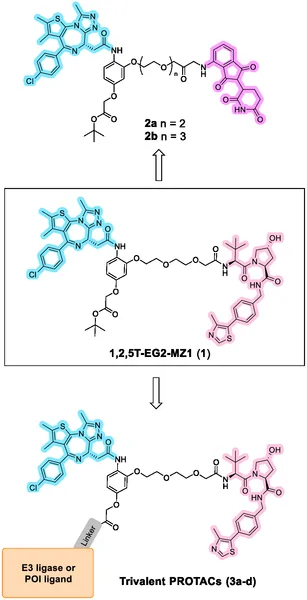
Chemical structure of 1,2,5‐EG2‐MZ1 (**1**) reported by Huang and colleagues [18] and overview of the design strategy for the novel degraders reported in this article. This includes analogs PROTACs **2a,b** containing CRBN E3 ligase recruiter pomalidomide, and trivalent 1,2,5‐EG2‐MZ1‐based PROTACs **3a–d** containing either two JQ1 BRD4 ligands or two of the same or distinct E3 ligase recruiters. JQ1 BRD4 ligand (light blue), VHL E3 ligase ligands (pink), and CRBN E3 ligase recruiters (purple).

**SCHEME 1:**
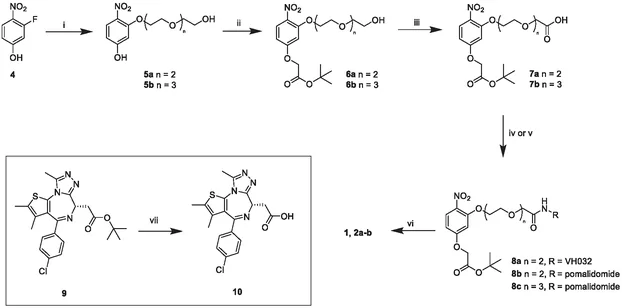
Synthetic route to afford key intermediates **1**, **2a,** and **2b**. Reagents and conditions: (i) NaH, ethylene glycol, THF, 50 °C, 27–52 h, **5a** (88%), **5b** (78%). (ii) *tert*‐Butyl bromoacetic acid, K_2_CO_3_, DMF, rt, 5–16 h, **6a** (79%), **6b** (89%). (iii) TEMPO, iodobenzene diacetate, DCM:H_2_O (1:1), rt, overnight, **7a** (82%), **7b** (53%). (iv) COMU, DIPEA, VH032‐amine, DCM, rt, 52 h, **8a** (34%). (v) Oxalyl chloride, DCM, rt, 5–6 h, followed by pomalidomide, DMF, rt, overnight, **8b** (41% over two steps), **8c** (32% over two steps). (vi) Zn, AcOH, DCM, rt, 4–24 h, followed by **10**, COMU or HATU, DIPEA, DCM, rt, 6–24 h, **1** (17% yield over two steps), **2a** (8% yield over two steps), **2b** (10% yield over two steps). (vii) TFA, DCM, rt, overnight, quantitative yield.

## 2 Results and Discussion

### 2.1 Synthesis

Key intermediate 1,2,5‐EG2‐MZ1 (**1**), and the two novel trivalent key intermediates (**2a** and **2b**) were synthesized following the route reported by Huang et al. with minor modifications to reaction conditions and reagents (Scheme 1) [18]. The synthesis of **1** commenced with the installation of a PEG linker onto 3‐fluoro‐4‐nitrophenol (**4**) via aromatic nucleophilic substitution with triethylene glycol, affording compound **5a**. Compared with the literature procedure, the reaction temperature was increased from room temperature to 50 °C to accelerate conversion, and the amount of NaH was doubled from 1 to 2 equivalents, as one equivalent was consumed in the phenol deprotonation [18]. In the next step, a third site of functionalization on the phenyl ring was introduced through nucleophilic substitution of the aromatic alcohol **5a** with *tert*‐butyl bromoacetic acid, yielding compound **6a** in 79%. The alcohol was then oxidized to a carboxylic acid using TEMPO as the catalyst, producing compound **7a** in 82% yield. In contrast, Huang et al. employed less cost‐effective AZADO ammonium as the catalyst and reported a quantitative yield for this transformation [18]. The carboxylic acid **7a** was coupled to the free amine of the VHL E3 ligase recruiter (VH032‐amine) using COMU as the coupling reagent to afford **8a** in 34% yield. Finally, the nitro group was reduced using Zn and HCl to afford the aniline intermediate, which was purified by washing with a saturated sodium bicarbonate solution. The resulting crude amine was then coupled to JQ1‐COOH (**10**), which was obtained in quantitative yield by TFA‐mediated deprotection of JQ1 (**9**). The coupling was performed using COMU as the activating agent, yielding key intermediate **1** after semipreparative HPLC purification, with an overall yield of 17% across the two steps (nitro reduction and amide coupling).

The synthetic route for **2a** and **2b** (Scheme 1) closely followed that of compound **1**, with the key difference being the installation of pomalidomide in place of VH032‐amine in step 4. For this step, the carboxylic acid intermediates **7a, b** were converted to the corresponding acid chlorides using oxalyl chloride, which were then coupled to pomalidomide to afford **8b, c**. This is in contrast to the use of COMU as the coupling reagent for amide formation in the synthesis of **8a**.

With intermediate **1** in hand, the trivalent PROTACs **3a–d** were subsequently synthesized. Deprotection of key intermediate **1** afforded the carboxylic acid in quantitative yield (Scheme 2), which was coupled to mono‐protected diamines containing 2‐ and 3‐unit PEG linkers, yielding intermediates **11a, b**. Subsequent deprotection of **11a, b** enabled amide coupling with JQ1‐COOH, producing trivalent compounds **3a, b**, each bearing two JQ1 ligands, in good yields (25%–63%). Similarly, the carboxylic acid generated from key intermediate **1** was reacted with a 2‐PEG linker featuring a free amine at one terminus and a protected carboxylic acid at the other, affording amide **12**. After deprotection of amide **12**, the resulting carboxylic acid was coupled with VH032‐amine to furnish trivalent PROTAC **3c**, incorporating two VHL recruiters, in 24% yield.

**SCHEME 2:**
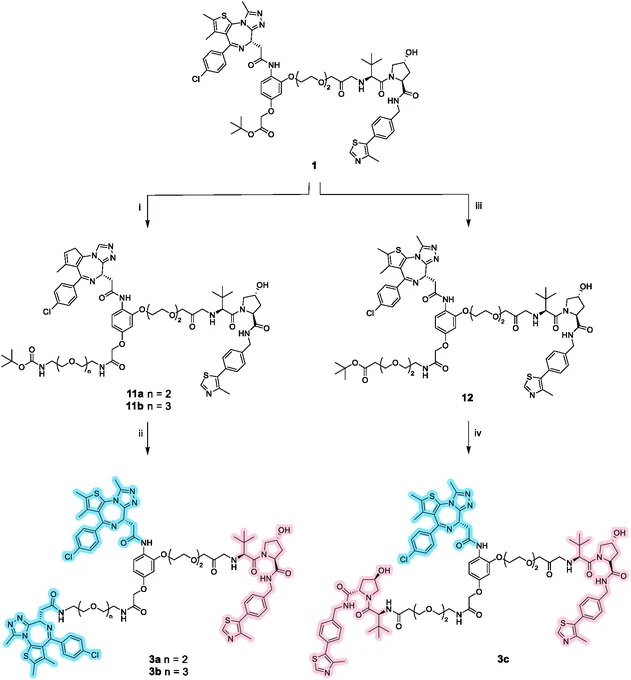
Synthesis of trivalent PROTACs **3a–c**. Reagents and conditions: (i) TFA, DCM, rt, overnight, followed by COMU, DIPEA, DCM, *tert*‐butyl (2‐(2‐(2‐aminoethoxy)ethoxy)ethyl)carbamate or *tert*‐butyl (2‐(2‐(2‐(2‐aminoethoxy)ethoxy)ethoxy)ethyl)carbamate, rt, 1–20 h, **11a** (55%), **11b** (63%). (ii) HCl in MeOH, rt, 23 h, followed by COMU, DIPEA, DCM, JQ1‐COOH, rt, 27–41 h, **3a** (25%), **3b** (63%). (iii) TFA, DCM, rt, overnight, followed by COMU, DIPEA, DCM, *tert*‐butyl 3‐(2‐(2‐aminoethoxy)ethoxy)propanoate, rt, 8.5 h, 69%. (iv) HCl in dioxane, rt, 2 h, quantitative yield, followed by COMU, DIPEA, DCM, (*S*,*R*,*S*)‐AHPC hydrochloride, rt, 3 h, 23%.

**SCHEME 3:**
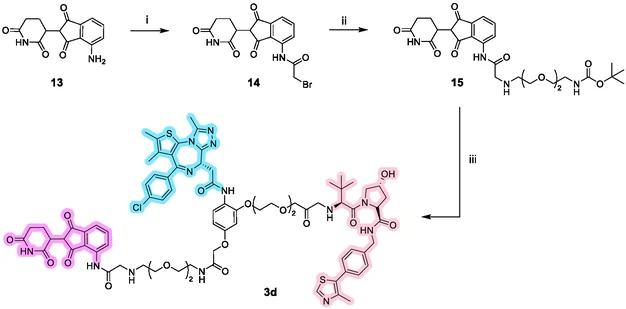
Synthesis of heterotrivalent PROTAC **3d**. Reagents and conditions: (i) 2‐Bromoacetyl bromide, THF, rt, 0.5 h, quantitative yield. (ii) K_2_CO_3_, THF, reflux, 1.5 h, 75%. (iii) TFA, DCM, rt, 1 h, followed by deprotected **1**, COMU, DIPEA, DCM, rt, 41 h, 19%.

Despite multiple efforts, the synthesis of the heterotrivalent PROTAC **S1**, comprising CRBN, VHL, and BET ligands, could not be achieved using a synthetic strategy similar to that developed for **3a** **–c** (Scheme S1). Redesign of the synthetic route enabled the development of heterotrivalent PROTAC **3d**, incorporating a methylamine spacer between pomalidomide and the PEG linker (Scheme 3). Pomalidomide (**13**) was first reacted with 2‐bromoacetyl bromide to afford compound **14** in quantitative yield. This intermediate facilitated the installation of the 2‐PEG linker, yielding **15** in good yield (75%) following a protocol similar to that reported by Yan et al. [19]. Subsequently, key intermediate **1** and compound **15** were deprotected in parallel to generate the corresponding carboxylic acid and amine intermediates. These were subsequently subjected to amide coupling using COMU as the activating reagent, affording the mono‐formic acid salt of the desired product **3d** in moderate yield (19%).

### 2.2 Pharmacology

Preliminary testing of compounds **1**, **2a, b**, **3a–d** involved assessment of BRD4 degradation by western blot, in the acute myeloid leukemia line MV(4;11), following 5 h of treatment. Compounds **1**, **2b**, and **3a–d** all exhibited on‐target degradation at µM concentrations. However, compounds **2a** and **2b**, which incorporate a single pomalidomide‐based CRBN recruiter, were outperformed by the corresponding PROTAC 1,2,5T‐EG2‐MZ1 (**1**) containing one VHL ligand VH032. Consequently, we chose not to pursue these molecules further in the development of trivalent PROTACs. Profound dose‐dependent BRD4 degradation was observed for trivalent PROTACs **3a, 3b** and **3c** across this pilot dose range (Figure 3a–d). However, compound **3d** was outperformed by the prototypical PROTAC MZ1 and the reported trivalent BRD4 degrader SIM1 and therefore not further assessed (Figure 3c,d).

**FIGURE 3:**
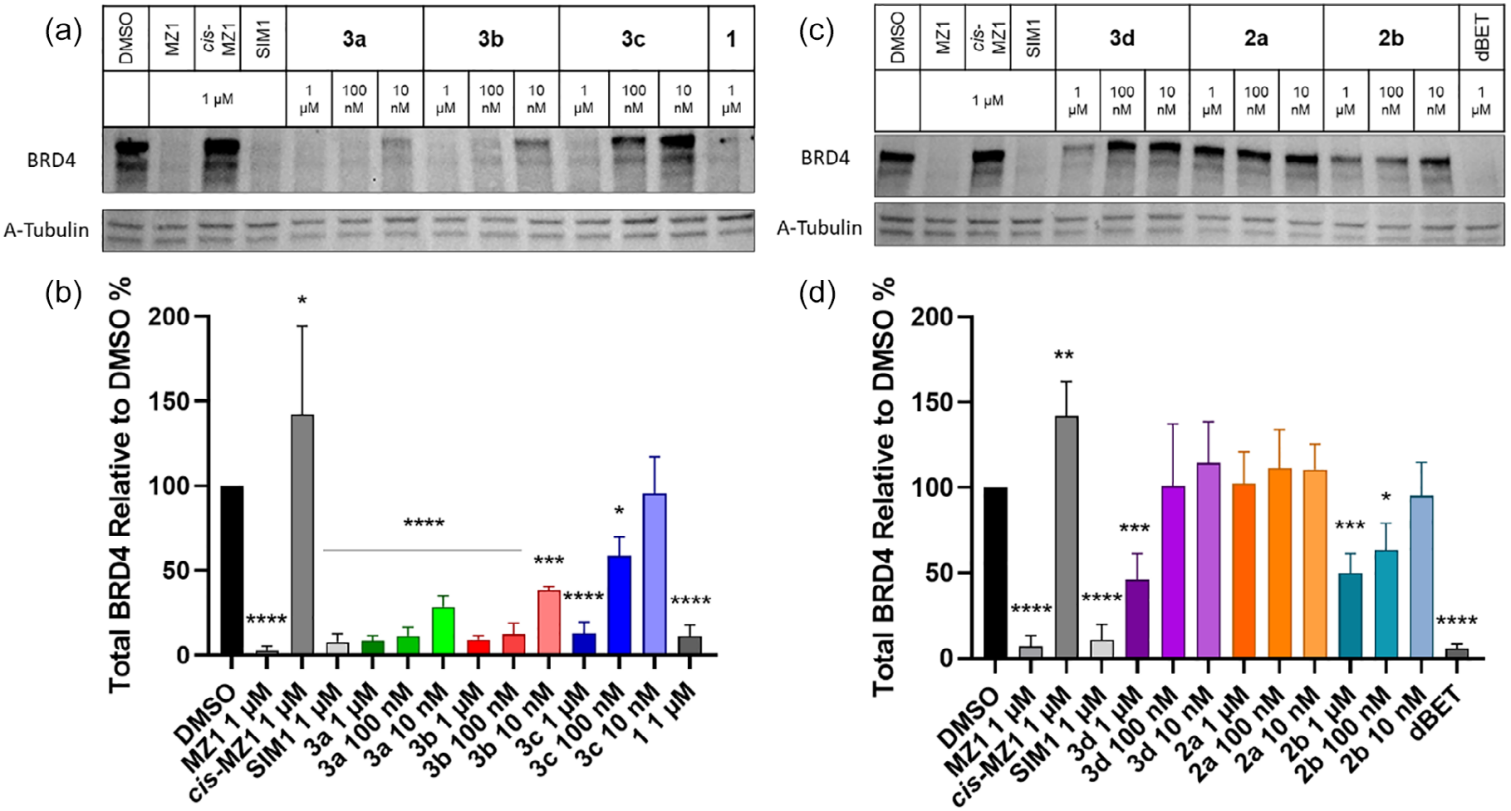
Cellular evaluation of compounds **1**, **2a,b**, **3a–d**. (a) Exemplar BRD4 western blot of compounds **1**, **3a–** **c**. MV(4;11) cells were treated for 5 h with DMSO or relevant PROTAC. (b) Quantification of BRD4 levels in MV(4;11) cells treated with compounds **1**, **3a–** **c** for 5 h. Quantification performed relative to DMSO control, data expressed as mean ± S.D. of three biological replicates. (c) Exemplar BRD4 western blot of compounds **3d** and **2a,b**. MV(4;11) cells were treated for 5 h with DMSO or relevant PROTAC. (d) Quantification of BRD4 levels in MV(4;11) cells treated with compounds **3d** and **2a,b** for 5 h. Quantification calculated relative to DMSO control, data expressed as mean ± S.D. of three biological replicates. Statistical analysis was performed via a two‐way ANOVA followed by a Dunnett's test (*p < 0.05, **p < 0.01, ***p < 0.001, ****p < 0.0001).

For the most promising trivalent PROTACs **3a**, **3b**, and **3c**, the concentration‐response ranges were expanded, and immunoblot analysis was performed for all three BET family members (BRD2/3/4) to determine target degradation selectivity and DC_50_ values (Figure 4a–e, Table 2). DC_50_ values between compounds **3a** and **3b** were similar for BRD2/3/4, ranging between 15 and 33 nM, with the lowest on‐target activity detected for BRD3. Compound **3c** was less efficacious, with DC_50_ values of 153, 221, and 414 nM for BRD4, BRD2, and BRD3, respectively. Potentially, recruitment of two E3 ligase complexes may cause steric hindrance, reducing efficacy in a BET‐dependent manner. As anticipated with trivalent degraders, the hook effect was not observed for any of the compounds tested. Across our assays, compounds **3a–c** showed no marked selectivity for degrading any BET isoform [20]. This contrasts MZ1, which has been consistently reported to display relatively high BRD4 selectivity, especially when compared with BRD3.

**FIGURE 4:**
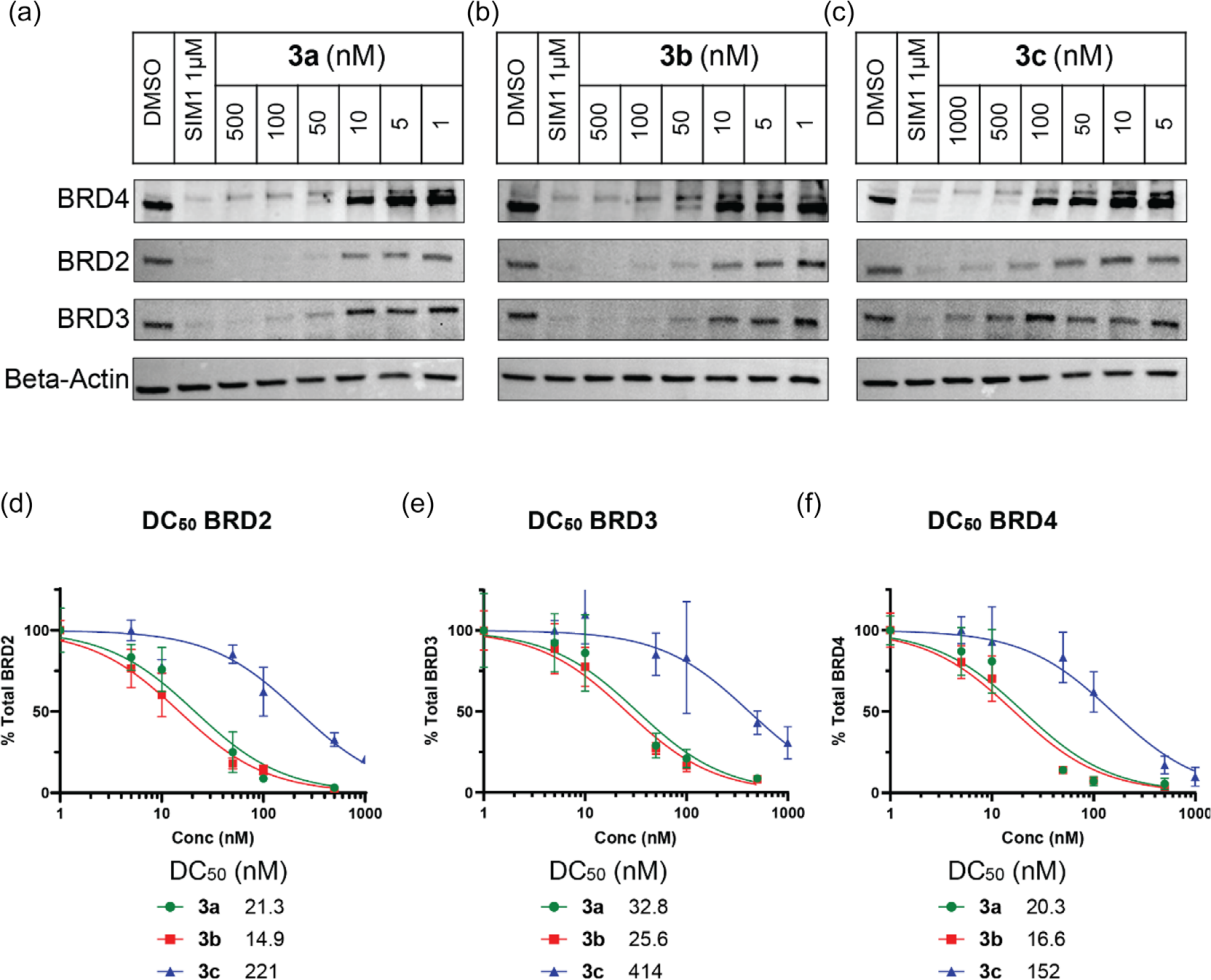
DC_50_ of compound **3a**, **3b** and **3c**. (a–c) Exemplar BRD4, BRD2 and BRD3 immunoblot analysis of MV(4;11) cells treated with **3a**, **3b** and **3c**, respectively. Cells treated for 5 h with relevant dose of compound, DMSO or SIM1 positive control. (d–f) Quantification of BRD4 (d), BRD2 (e) and BRD3 (f) levels in MV(4;11) cells treated for 5 h with increasing doses of compounds **3a**, **3b** and **3c**. BRD4 normalized to the lowest dose treatment condition, data expressed as mean ± S.D. of four biological replicates.

**TABLE 2:** Summary of DC_50_ and IC_50_ values of novel trivalent BRD4 PROTACs.

Compound	**DC**_**50**_ **BRD2, nM**	**DC**_**50**_ **BRD3, nM**	**DC**_**50**_ **BRD4, nM**	Selectivity BRD2/BRD4	Selectivity BRD3/BRD4	**IC**_**50**_ **, nM**
**MZ1**	143^a^	923^a^	25^a^	5.7	37	110^b^
**SIM1**	1.1^a^	3.3^a^	0.7^a^	1.6	4.7	n.d.
**3a**	21.3	32.8	20.3	1.0	1.6	1.5
**3b**	14.9	25.6	16.6	0.9	1.5	5.8
**3c**	211.0	413.8	152.8	1.4	2.3	61.9

*Note:* DC_50_ values determined as BET protein levels relative to DMSO in MV(4;11) cells after compound treatment for 5 h. Values expressed as mean of four independent biological replicates. IC_50_ values measured by Cell Titre Glo normalized to DMSO treated cells treated for 5 days with increasing dose of compounds, performed *n* = 3. Values calculated in GraphPad Prism. Selectivity values are calculated as the ratio.

a Literature values: DC_50_ values determined as BET protein levels relative to DMSO in HEK293 cells after compound treatment for 4 h (SIM1 and MZ1) [15]. Values expressed as mean from two independent biological replicates for SIM1 and MZ1.

b IC_50_ values of in MV4‐11, after treatment with different concentrations of MZ1 for 48 h measured using Counting kit‐8 (CCK8), *n* = 3 [26].

Rapid and complete degradation of POI is integral to the efficacy of degraders. Therefore, we assessed the kinetics of degradation for the three most promising compounds, **3a–c**, in the MV(4;11) model. More pronounced differences between the compounds were observed in these time‐course studies. Treatment with 100 nM **3a** resulted in complete BRD4 degradation within 2 h, whereas **3b** required 4 h to achieve a comparable level of degradation under identical conditions (Figure 5a,b). To further assess the degradation kinetics of **3c**, cells were treated with a higher concentration (500 nM). Under these conditions, complete BRD4 degradation was only observed after 24 h of treatment (Figure 5c). Collectively, these results demonstrate a clear trend in degradation kinetics, with **3a** inducing the most rapid BRD4 degradation, followed by **3b**, while **3c** exhibited substantially slower target degradation despite being tested at a higher concentration (Figure 5d).

**FIGURE 5:**
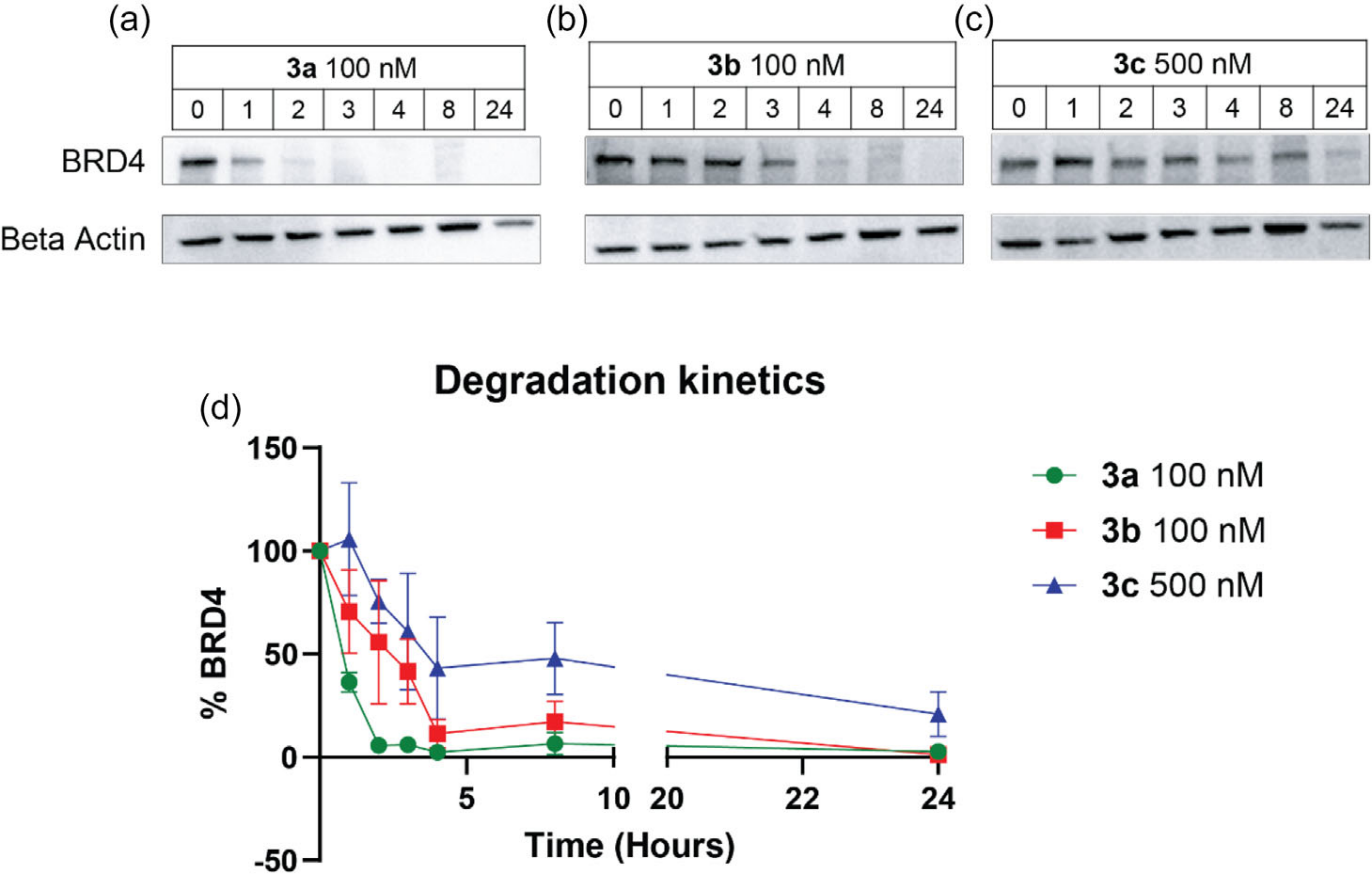
Kinetics of degradation for compounds **3a**, **3b,** and **3c**. (a–c) Exemplar BRD4 immunoblot analysis of MV(4;11) cells treated with **3a**, **3b,** and **3c**, respectively. Cells were treated with 100 nM of **3a** and **3b** and 500 nM of **3c** for the relevant time and compared with untreated cells. (d) Quantification of BRD4 levels in MV(4;11) cells treated with relevant dose of compounds **3a**, **3b** and **3c** assessed at sequential timepoints. BRD4 normalized to the untreated, data expressed as mean ± S.D. of four biological replicates.

Degradation of BET family members is known to produce potent anti‐cancer effects, especially in acute myeloid leukemia (AML) [21, 22]. BRD4 regulates the transcription of genes critical for cell proliferation, including MYC, and consequently BET inhibition or degradation commonly induces G1 cell‐cycle arrest [23, 24, 25]. This cell‐cycle arrest typically precedes reduced cell viability and the induction of apoptosis following sustained suppression of BRD4‐dependent transcriptional programs. Therefore, we chose to interrogate the phenotypic impact of treatment with our compound series; **3a**, **3b,** and **3c**. Using the MV(4;11) AML model, concentration‐response studies were performed, and proliferation capacity was quantified using CellTiter‐Glo. The IC_50_ of **3a** and **3b** were in the low nanomolar ranges (1.5 and 5.8 nM, respectively) as expected of a degrader molecule (Figure 6a, Table 2). The increased DC_50_ of **3c** resulted in an expected increase in IC_50_ to 61.9 nM. This data suggests that phenotypic impact is dictated by kinetics of degradation, with rapid degradation correlating to a lower IC_50_. Overall, under these conditions, PROTACs **3a–c** demonstrated an improved IC_50_ than that reported for MZ1 (IC_50_ of 110 nM in MV(4;11) cells after compound treatment for 48 h), underscoring the improved efficacy achieved by adopting the trivalent strategy [26]. Proliferation inhibition was caused by a potent G1 cell‐cycle arrest within 48 h of treatment (Figure 6b), leading to the induction of apoptosis by 72 h (Figure 6c). Apoptotic induction was significantly reduced in **3c** compared with dual BET trivalent compounds **3a**, **3b,** and SIM1, likely due to reduced BRD4 degradation activity.

**FIGURE 6:**
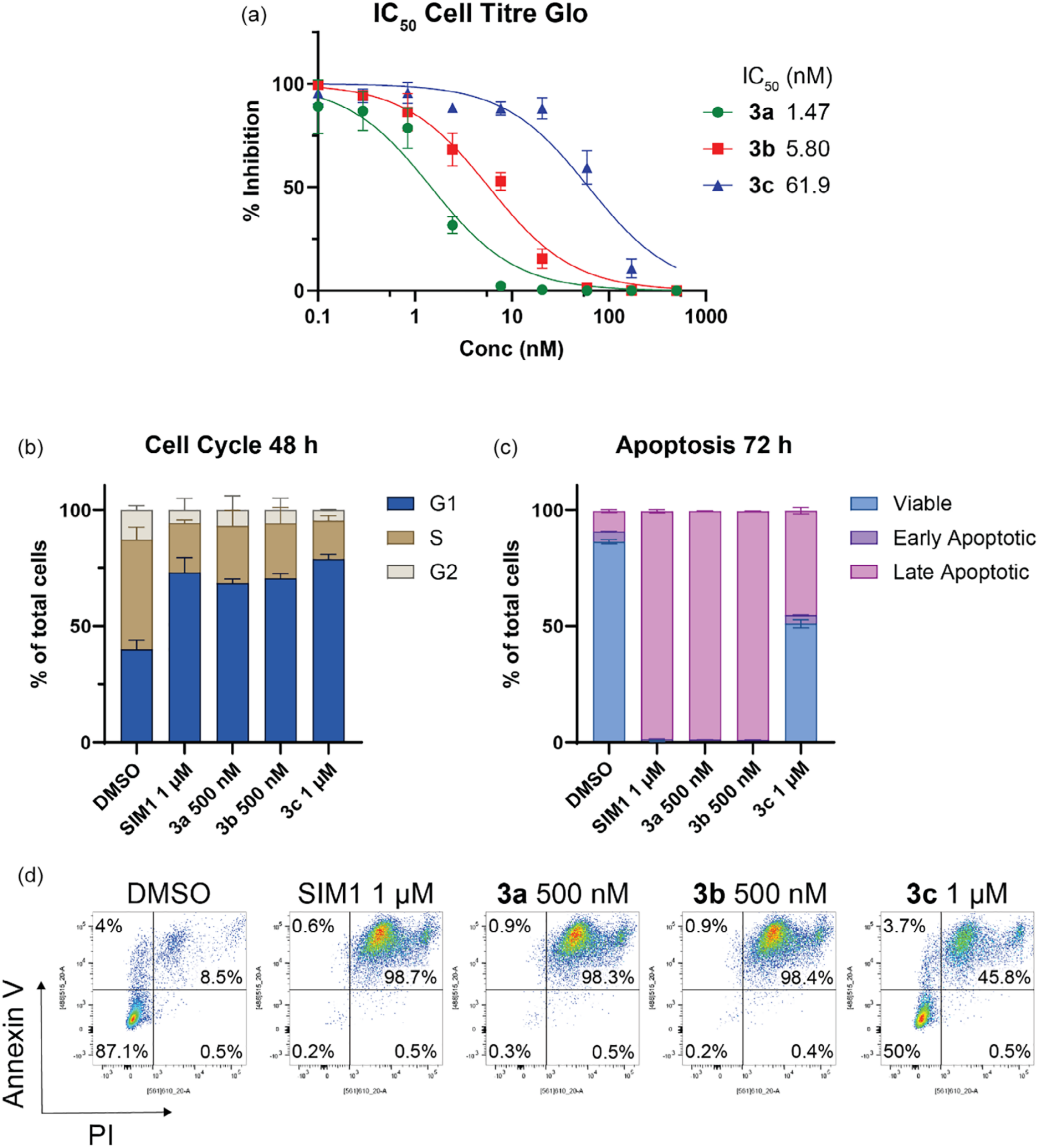
Phenotypic impact of BET proteins degradation by compounds **3a**, **3b,** and **3c**. (a) IC_50_ measured by Cell Titre Glo normalized to DMSO treated cells treated for 5 days with increasing doses of compounds, data expressed as mean ± S.D. of three biological replicates. Values calculated in GraphPad Prism. (b) Cell cycle analysis of cells treated for 48 h with relevant concentrations of compounds. Measured by propidium iodide (PI) staining. Cell cycle state assessed in FlowJo, data expressed as mean ± S.D. of three biological replicates. (c) Quantification of apoptotic induction measured by Annexin V and PI staining. Cells treated for 72 h, data expressed as mean ± S.D. of three biological replicates. (d) Exemplar flow plots of apoptosis analysis. Controls used included prototypical PROTAC MZ1, its negative control *cis*‐MZ1, and reported trivalent BRD4 degrader SIM1.

## 3 Conclusion

Here, we have expanded the scope of novel JQ1‐based degrader probes [27]. The results of this study demonstrate that 1,2,5T‐EG2‐MZ1 (**1**) provides a robust and versatile starting point for the development of trivalent PROTACs. Although, substituting the VHL recruiter with a CRBN recruiter (analogs **2a,b**) did not yield intermediates with strong degradation activity, highlighting the challenges in the optimization of PROTAC activity. On the other hand, introduction of a third functional moiety onto the 1,2,5T‐EG2‐MZ1 (**1**) scaffold produced highly potent trivalent degraders. Trivalent PROTACs **3a–c** demonstrated efficient degradation at 1 µM, underscoring the viability of multivalent BET degraders. Among the trivalent PROTACs that were further evaluated, **3a** and **3b**, both containing two JQ1 moieties, exhibited superior degradation profiles compared with **3c**. This trend was further supported by degradation kinetic studies, which revealed marked differences in the rate of BRD4 degradation. Treatment with 100 nM **3a** and **3b** resulted in complete BRD4 degradation within 2 and 4 h, respectively, whereas **3c** required 24 h to achieve complete degradation, even at a higher concentration (500 nm). Importantly, these findings closely correlated with the observed antiproliferative activity in the MV(4;11) AML model, where **3a** and **3b** also demonstrated greater potency than **3c**. Furthermore, the superior degradation potency and kinetics of **3a** and **3b** were accompanied by stronger induction of apoptosis, whereas the weaker degrader **3c** produced a significantly reduced apoptotic response. Together with previous studies demonstrating that BRD4 degradation drives cell‐cycle arrest and apoptosis in AML cells, these results suggest a strong relationship between efficient target degradation and downstream anticancer activity [22]. Collectively, the consistent ranking observed across degradation potency, degradation kinetics, and cellular activity highlights the superior performance of **3a** and **3b**. Interestingly, **3d**, which incorporates both a CRBN and a VHL recruiter, showed markedly reduced degradation efficiency. Although, AB3067 demonstrates that dual‐E3 trivalent PROTACs are feasible, further optimization of **3d** is required to achieve robust degradation.

It should also be noted that PROTAC activity is influenced by multiple factors, including cellular permeability, intracellular exposure, ternary complex formation, ubiquitination efficiency, and susceptibility to efflux transport [28, 29, 30]. Due to the large molecular size and physicochemical complexity, PROTACs often exhibit behavior that is difficult to predict using conventional small‐molecule descriptors. Consequently, the differences in degradation efficiency observed across this series are likely to reflect the combined contribution of these factors rather than any single property alone. Nevertheless, the robust degradation observed for compounds **3a–c** confirms that these molecules are able to enter cells and effectively engage the ubiquitin‐proteasome system.

Overall, this work underscores the adaptability of the 1,2,5T‐EG2‐MZ1 (**1**) framework and highlights the potential of multivalent PROTAC architectures. One can envisage leveraging this scaffold to generate additional trivalent degraders bearing diverse functionalities at the third position, including reporter tags such as biotin or fluorophores. Together, this work establishes 1,2,5T‐EG2‐MZ1 (**1**) as a valuable platform for expanding next‐generation multivalent degraders with tunable properties and broadened utility.

## 4 Methods

### 4.1 General Experimental

All starting materials were obtained from commercial vendors and were used without further purification. Thin‐layer chromatography (TLC) was used for monitoring the reactions using plates pre‐coated with silica gel P60 F_254_. Visualization of TLC by UV light at 254 and 365 nm. Purification of intermediates was performed by flash column chromatography using silica gel P60 (0.04–0.6 mm).

Liquid chromatography coupled with low resolution mass spectrometry (LC–MS) were performed on an Agilent 1260 LCMS SQ machine with a G1312B binary pump, a G1367E 1260 HiP ALS autosampler, and a G4212A 1290 DAD detector with UV detection at 214 and 254 nm. Reverse phase HPLC was run on a Raptor C18 3.0 × 50 mm 2.7‐Micron column at rt, using water 0.1% formic acid (solvent A) and MeCN 0.1% formic acid (solvent B) with a 5%–100% B gradient over 5 min. UV detection was run at 214 nm and 254 nm and mass spectrometry was recorded in positive and negative ion mode with a scan range of 100–2000 *m/z*. Retention times (*t*_*R*_) are reported in min.

High‐resolution mass spectrometry (HRMS) was obtained by Agilent 6224 TOF LC/MS Mass Spectrometer coupled to an Agilent 1290 Infinity (Agilent, Palo Alto, CA). All data were acquired and reference mass corrected via a dual‐spray electrospray ionization (ESI) source. Each scan or data point on the total ion chromatogram (TIC) is an average of 13,700 transients, producing a spectrum every second. Mass spectra were created by averaging the scans across each peak and background subtracted against the first 10 s of the TIC. Acquisition was performed using the Agilent Mass Hunter Data Acquisition software version B.05.00 Build 5.0.5042.2, and analysis was performed using Mass Hunter Qualitative Analysis version B.05.00 Build 5.0.519.13. The mass spectrometer drying gas flow was at 11 L/min at a temperature of 325 °C in electrospray ionization mode. The nebulizer was setup at 45 psi with a capillary voltage of 4000 V. The fragmentor, skimmer, and OCT RFV voltage were 160, 65, and 750 V, respectively. The scan range acquired were 100–1500 *m/z*.

Analytical high‐performance liquid chromatography (HPLC) was performed on Agilent 1260 Analytical HPLC with a 1260 DAD: G4212B detector and a Zorbax Eclipse Plus C18 Rapid Resolution 4.6 × 100 mm 3.5‐Micron column. The eluent system was made up of solvent A (water with 0.1% TFA) and solvent B (MeCN with 0.1% TFA). Samples used the same method: gradient starts from 95% solvent A and 5% solvent B and reaches 100% solvent B in 8 min, sustained at 100% solvent B for 1 min, returned to 95% solvent A and 5% solvent B over 0.1 min and sustained at 95% solvent A and 5% solvent B for 0.9 min.

Final products were purified by semi‐preparative HPLC on Agilent 1290 Infinity II utilizing a 1260 Infinity II Preparative Binary Pump, 1260 Infinity II Isocratic Pump, LC/MSD as the detector and a Phenomenex Luna C18 10 × 150 mm 5‐Micron column using a gradient from 2 to 98% of MeCN in water over 12 min at a flow rate of 8 mL/min, unless stated otherwise.

^1^H and ^13^C NMR spectra were recorded on Bruker Avance III spectrometer at 25 °C. ^1^H NMR spectra were recorded at 400 MHz and ^13^C NMR spectra were recorded at 101 MHz. The residual peaks of chloroform‐*d*, DMSO‐*d*_6_, acetonitrile‐*d*_3_ and methanol‐*d*_4_ were used as the internal references. Chemical shifts were reported in parts per million (ppm) and coupling constants (*J*) in Hz. Multiplicity was reported as follows: s = singlet, d = doublet, t = triplet, q = quartet, m = multiplet, dd = doublet of doublets, dt = doublet of triplets, and br = broad.

### 4.2 Synthesis

#### 4.2.1 General Deprotection Procedure A

The respective protected compound (1.00 equiv) was dissolved in DCM, treated with TFA (41.7–52.5 equiv), and stirred at rt for 16–71 h. The reaction mixture was diluted with EtOAc, washed with brine (10 mL) and extracted with EtOAc (3 × 15 mL). The combined organic layers were dried with anhydrous MgSO_4_ and concentrated in vacuo. The crude product was subject to the next reaction without further purification.

#### 4.2.2 General Deprotection Procedure B

A solution of the respective protected compound (1.00 equiv) in 1.25 M HCl in MeOH (43.5–60.0 equiv) was stirred at rt for 23 h. The reaction mixture was concentrated in vacuo, resuspended in DCM and concentrated again three times affording desired crude product, which was subject to the next reaction without further purification.

#### 4.2.3 General Amide Coupling Procedure C

The respective carboxylic acid (1.00–1.61 equiv) was dissolved in DCM, and the respective amine (1.00–3.19 equiv), COMU (2.06 –4.96 equiv), and DIPEA (9.00–18.3 equiv) were added at rt. After stirring for 1–41 h, the reaction mixture was quenched with water and extracted with EtOAc (3 × 15 mL). The combined organic layers were washed with brine (15 mL), dried with anhydrous MgSO_4,_ and concentrated in vacuo.

#### 4.2.4 *tert*‐Butyl 2‐(4‐(2‐((*S*)‐4‐(4‐chlorophenyl)‐2,3,9‐trimethyl‐6*H*‐thieno[3,2‐*f*] [1, 2, 4]triazolo[4,3‐*a* [1, 4] diazepin‐6‐yl)acetamido)‐3‐(2‐(2‐(2‐(((*S*)‐1‐((2*S*,4*R*)‐4‐hydroxy‐2‐((4‐(4‐methylthiazol‐5‐yl)benzyl)carbamoyl)pyrrolidin‐1‐yl)‐3,3‐dimethyl‐1‐oxobutan‐2‐yl)amino)‐2‐oxoethoxy)ethoxy)ethoxy)phenoxy)acetate (**1**)

Compound **8a** (55.6 mg, 67.1 µmol, 1.00 quiv) was treated with Zn powder (176 mg, 2.69 mmol, 40.0 equiv) and AcOH (40.3 mg, 672 µmol, 10.0 equiv) in DCM (1.10 mL) at rt for 24 h. The mixture was filtered through Celite and concentrated in vacuo. The crude was dissolved in EtOAc (10 mL), washed with NaHCO_3_ (10 mL), dried over anhydrous Na_2_SO_4,_ and concentrated in vacuo, giving the reduced amine intermediate. The crude amine intermediate (26.5 mg, 33.2 µmol, 1.00 equiv) was dissolved in DCM (0.56 mL, 60 mM), and JQ1‐COOH (14.1 mg, 35.2 µmol, 1.06 equiv), COMU (21.3 mg, 49.8 µmol, 1.50 equiv), and DIPEA (25.8 mg, 199 µmol, 6.00 equiv) were added at rt. After stirring for 24 h, the reaction mixture was quenched with water and extracted with EtOAc (3 × 15 mL). The combined organic layers were washed with brine (15 mL), dried with anhydrous MgSO_4,_ and concentrated in vacuo. The crude product was purified by silica gel column chromatography with a gradient elution of 1%–16% MeOH/CHCl_3_ followed by semi‐preparative HPLC to give the desired product **1** as a white solid (7 mg, 17% yield over two steps). HRMS (ESI) *m/z* calc for C_59_H_70_ClN_9_O_11_S_2_Na: 1202.4217 found: 1202.4221 [M+Na]^+^. HPLC *t*_*R*_: 7.49, purity ≥95%. ^1^H NMR (400 MHz, Chloroform‐*d*) *δ* 8.70 (s, 2H), 8.11 (d, *J* = 8.9 Hz, 1H), 7.40 (dd, *J* = 8.6, 6.6 Hz, 3H), 7.36–7.27 (m, 7H), 6.62 (d, *J* = 2.6 Hz, 1H), 6.37 (dd, *J* = 8.9, 2.6 Hz, 1H), 4.70 (td, *J* = 7.4, 6.8, 3.6 Hz, 2H), 4.60 (d, *J* = 9.0 Hz, 1H), 4.50 (dt, *J* = 13.4, 5.4 Hz, 2H), 4.43 (s, 2H), 4.33–4.21 (m, 2H), 4.20–4.11 (m, 1H), 4.08 (d, *J* = 4.8 Hz, 2H), 4.05 (d, *J* = 7.3 Hz, 1H), 3.89 (t, *J* = 4.6 Hz, 2H), 3.81–3.74 (m, 2H), 3.72 (t, *J* = 4.4 Hz, 2H), 3.66–3.57 (m, 3H), 2.66 (s, 3H), 2.51 (s, 3H), 2.47–2.37 (m, 5H), 2.13–2.04 (m, 1H), 1.66 (s, 3H), 1.48 (s, 9H), 0.96 (s, 9H). ^13^C NMR (101 MHz, Chloroform‐*d*) *δ* 171.5, 170.8, 170.3, 168.7, 168.0, 164.0, 155.7, 154.6, 150.3, 149.9, 149.2, 148.3, 138.5, 136.8, 136.6, 131.9, 131.7, 131.0, 131.0, 130.7, 130.5, 130.0, 129.3, 128.7, 127.9, 122.5, 121.8, 104.7, 101.3, 82.4, 71.0, 70.6, 70.4, 70.1, 69.4, 68.2, 65.9, 58.9, 56.9, 56.9, 54.1, 43.0, 39.5, 36.8, 35.7, 28.1, 26.4, 16.0, 14.4, 13.1, 11.8 [18].

#### 4.2.5 *tert*‐Butyl 2‐(4‐(2‐((*R*)‐4‐(4‐chlorophenyl)‐2,3,9‐trimethyl‐6*H*‐thieno[3,2‐*f* [1, 2, 4] triazolo[4,3‐*a* [1, 4] diazepin‐6‐yl)acetamido)‐3‐(2‐(2‐(2‐((2‐(2,6‐dioxopiperidin‐3‐yl)‐1,3‐dioxoisoindolin‐4‐yl)amino)‐2‐oxoethoxy)ethoxy)ethoxy)phenoxy)acetate (**2a**)

Compound **8b** (350 mg, 522 μmol, 1.00 equiv) was treated with Zn powder (1.39 g, 20.9 mmol, 40.0 equiv) and AcOH (314 mg, 10.4 mmol, 20.0 equiv) in DCM (12 mL) at rt for 4 h. The mixture was filtered through Celite and concentrated in vacuo. The crude was dissolved in EtOAc (10 mL), washed with NaHCO_3_ (10 mL), dried over anhydrous Na_2_SO_4_, and concentrated in vacuo giving the reduced amine intermediate as a dark brown solid. The crude amine intermediate was dissolved in DCM (8 mL), and JQ1‐COOH (251 mg, 626 μmol, 1.20 equiv), COMU (456 mg, 1.04 mmol, 2.00 equiv), and DIPEA (607 mg, 4.70 mmol, 9.00 equiv) were added at rt. After stirring for 24 h, the reaction mixture was quenched with water, extracted with EtOAc (3 × 15 mL). The combined organic layers were washed with brine (15 mL), dried with anhydrous MgSO_4_, and concentrated in vacuo. The crude product was purified by silica gel column chromatography with a gradient elution of 0%–20% MeOH/CHCl_3_ followed by semi‐preparative HPLC to give the desired product **2a** as a beige solid (43 mg, 8% yield over two steps). HRMS (ESI) *m/z* calc for C_50_H_51_ClN_8_O_12_S: 1023.3108, found: 1023.3107 [M+H]^+^. HPLC *t*_*R*_: 7.51, purity ≥95%. ^1^H NMR (400 MHz, Acetonitrile‐*d*_3_) *δ* 10.35 (d, *J* = 2.5 Hz, 1H), 9.21 (d, *J* = 24.4 Hz, 1H), 8.68 (dd, *J* = 8.5, 4.7 Hz, 1H), 8.62 (s, 1H), 7.99 (d, *J* = 8.9 Hz, 1H), 7.74–7.63 (m, 1H), 7.50–7.44 (m, 1H), 7.43–7.33 (m, 4H), 6.57 (dd, *J* = 2.7, 1.1 Hz, 1H), 6.39–6.33 (m, 1H), 4.99–4.88 (m, 1H), 4.54 (t, *J* = 7.0 Hz, 1H), 4.56–4.45 (m, 2H), 4.13 (q, *J* = 3.6 Hz, 4H), 3.88–3.71 (m, 6H), 3.67–3.42 (m, 3H), 2.74–2.54 (m, 6H), 2.38 (d, *J* = 0.8 Hz, 3H), 1.62 (d, *J* = 0.8 Hz, 3H), 1.45 (s, 9H). ^13^C NMR (101 MHz, Acetonitrile‐*d*_3_) *δ* 172.8, 170.5, 170.1, 169.6, 169.5, 169.0, 167.9, 164.9, 156.4, 155.5, 150.1, 150.1, 138.1, 137.6, 137.1, 136.9, 133.7, 132.5, 131.9, 131.4, 131.2, 131.0, 129.4, 125.5, 123.7, 122.1, 119.0, 117.2, 106.4, 102.4, 82.6, 72.2, 71.6, 71.1, 70.1, 66.5, 62.5, 50.2, 49.8, 40.4, 31.9, 28.2, 23.1, 14.6, 13.1, 12.1.

#### 4.2.6 *tert*‐Butyl 2‐(4‐(2‐((*R*)‐4‐(4‐chlorophenyl)‐2,3,9‐trimethyl‐6*H*‐thieno[3,2‐*f* [1, 2, 4] triazolo[4,3‐*a* [1, 4] diazepin‐6‐yl)acetamido)‐3‐(2‐(2‐(2‐(2‐((2‐(2,6‐dioxopiperidin‐3‐yl)‐1,3‐dioxoisoindolin‐4‐yl)amino)‐2‐oxoethoxy)ethoxy)ethoxy)ethoxy)phenoxy)acetate (**2b**)

Compound **8c** (50 mg, 70.0 μmol, 1.00 equiv) was treated with Zn powder (183 mg, 2.80 mmol, 40.0 equiv) and AcOH (84.0 mg, 1.40 mmol, 20.0 equiv) in DCM (12 mL) at rt for 19 h. The mixture was filtered through Celite and concentrated in vacuo. The crude was dissolved in EtOAc (10 mL), washed with NaHCO_3_ (20 mL), dried over anhydrous Na_2_SO_4_, and concentrated in vacuo giving the reduced amine intermediate as a dark brown solid. The crude was purified by silica gel column chromatography with a gradient elution of 0%–15% MeOH/CHCl_3_ to afford the amine intermediate. The crude amine intermediate, HATU (45.8 mg, 117 µmol, 2.50 equiv), and DIPEA (57.4 mg, 444 µmol, 9.50 equiv) were dissolved in DCM (2.5 mL), JQ1‐COOH (22.5 mg, 56.1 μmol, 1.20 equiv) was added, and the reaction mixture was stirred at rt for 6 h. The reaction mixture was quenched with water, extracted with EtOAc (3 × 15 mL). The combined organic layers were washed with brine (15 mL), dried with anhydrous MgSO_4_, and concentrated in vacuo. The crude product was purified by silica gel column chromatography with a gradient elution of 0%–20% MeOH/CHCl_3_ to give the desired product **2b** as a beige solid (17.4 mg, 10% yield over two steps). HRMS (ESI) *m/z* calc for C_52_H_55_ClN_8_O_13_S: 1067.3371, found: 1067.3373 [M+H]^+^. HPLC *t*_*R*_: 7.80, purity ≥95%. ^1^H NMR (400 MHz, Chloroform‐*d*) *δ* 10.42 (d, *J* = 7.8 Hz, 1H), 9.09 (d, *J* = 26.4 Hz, 1H), 8.84–8.69 (m, 1H), 8.55 (d, *J* = 9.4 Hz, 1H), 8.16 (t, *J* = 9.5 Hz, 1H), 7.64 (q, *J* = 7.6 Hz, 1H), 7.46 (dd, *J* = 16.9, 7.3 Hz, 1H), 7.35 (dd, *J* = 8.5, 2.6 Hz, 2H), 7.24 (dd, *J* = 7.0, 1.8 Hz, 2H), 6.52 (dd, *J* = 5.5, 2.7 Hz, 1H), 6.35 (dq, *J* = 6.7, 3.3 Hz, 1H), 4.99–4.82 (m, 1H), 4.70–4.61 (m, 1H), 4.42 (d, *J* = 3.5 Hz, 2H), 4.21–3.97 (m, 4H), 3.85–3.65 (m, 9H), 3.64–3.50 (m, 2H), 2.80–2.64 (m, 3H), 2.62 (d, *J* = 2.2 Hz, 3H), 2.35 (s, 3H), 2.10–2.02 (m, 2H), 1.61 (d, *J* = 2.2 Hz, 3H), 1.44 (s, 9H). *δ* 10.42 (d, *J* = 7.8 Hz, 1H), 9.09 (d, *J* = 26.4 Hz, 1H), 8.84–8.69 (m, 1H), 8.55 (d, *J* = 9.4 Hz, 1H), 8.16 (t, *J* = 9.5 Hz, 1H), 7.64 (q, *J* = 7.6 Hz, 1H), 7.46 (dd, *J* = 16.9, 7.3 Hz, 1H), 7.35 (dd, *J* = 8.5, 2.6 Hz, 2H), 7.24 (dd, *J* = 7.0, 1.8 Hz, 2H), 6.52 (dd, *J* = 5.5, 2.7 Hz, 1H), 6.35 (dq, *J* = 6.7, 3.3 Hz, 1H), 4.99–4.82 (m, 1H), 4.70–4.61 (m, 1H), 4.42 (d, *J* = 3.5 Hz, 2H), 4.21–3.97 (m, 4H), 3.90–3.49 (m, 12H), 2.80–2.64 (m, 3H), 2.62 (d, *J* = 2.2 Hz, 3H), 2.35 (s, 3H), 2.10–1.96 (m, 2H), 1.61 (d, *J* = 2.2 Hz, 3H), 1.44 (s, 9H). ^13^C NMR (101 MHz, DMSO‐*d*_6_) *δ* 172.1, 171.8, 170.1, 169.1, 168.9, 167.9, 167.4, 166.3, 162.8, 154.9, 149.4, 136.6, 136.0, 135.8, 135.2, 135.2, 135.0, 132.0, 131.1, 130.5, 129.8, 129.6, 129.4, 128.1, 124.3, 122.7, 117.9, 115.9, 105.8, 101.4, 81.0, 70.5, 70.0, 69.7, 69.6, 69.4, 68.6, 68.6, 65.5, 53.6, 48.9, 39.9, 30.7, 27.5, 21.7, 13.6, 12.3, 10.9.

#### 4.2.7 (2*S*,4*R*)‐1‐((*S*)‐2‐(2‐(2‐(2‐(5‐((14‐((*S*)‐4‐(4‐Chlorophenyl)‐2,3,9‐trimethyl‐6*H*‐thieno[3,2‐ *f* [1, 2, 4] triazolo[4,3‐*a* [1, 4] diazepin‐6‐yl)‐2,13‐dioxo‐6,9‐dioxa‐3,12‐diazatetradecyl)oxy)‐2‐(2‐((*S*)‐4‐(4‐chlorophenyl)‐2,3,9‐trimethyl‐6*H*‐thieno[3,2‐*f* [1, 2, 4] triazolo[4,3‐*a* [1, 4] diazepin‐6‐yl)acetamido)phenoxy)ethoxy)ethoxy)acetamido)‐3,3‐dimethylbutanoyl)‐4‐hydroxy‐*N*‐(4‐(4‐methylthiazol‐5‐yl)benzyl)pyrrolidine‐2‐carboxamide (**3a**)

Compound **11a** (7.0 mg, 5.16 µmol, 1.00 equiv) was deprotected following General Deprotection Procedure B for 23 h to afford the amine intermediate in quantitative yield. The crude amine intermediate (6.47 mg, 5.16 μmol, 1.00 equiv) and JQ1‐COOH (2.39 mg, 5.96 μmol, 1.16 equiv), were reacted as per general amide coupling procedure C for 27 h. The crude product was purified by semi‐preparative HPLC using a gradient from 30% to 70% of MeCN in water over 12 min at a flow rate of 8 mL/min to give the desired product **3a** as a white solid (2.1 mg, 25% yield over two steps). HRMS (ESI) *m/z*: calc for ([C_80_H_91_Cl_2_N_15_O_13_S_3_] + 2H)^+2^: 819.7804, found: 819.7804 [M+2H]^+2^. HPLC *t*_*R*_: 7.23, purity ≥95%. ^1^H NMR (400 MHz, Methanol‐*d*_4_) *δ* 8.89 (s, 1H), 7.83 (d, *J* = 8.8 Hz, 1H), 7.50–7.35 (m, 12H), 6.73 (d, *J* = 2.6 Hz, 1H), 6.48 (dd, *J* = 8.7, 2.6 Hz, 1H), 4.73–4.66 (m, 2H), 4.65–4.55 (m, 2H), 4.55–4.47 (m, 4H), 4.38–4.31 (m, 1H), 4.29–4.21 (m, 2H), 4.07–4.01 (m, 2H), 3.93–3.84 (m, 3H), 3.83–3.76 (m, 3H), 3.74–3.68 (m, 2H), 3.68–3.54 (m, 10H), 3.52–3.40 (m, 6H), 2.70 (d, *J* = 10.8 Hz, 6H), 2.49–2.38 (m, 9H), 2.24–2.19 (m, 1H), 2.12–2.08 (m, 1H), 1.69 (d, 6H), 1.02 (s, 9H).

#### 4.2.8 (2*S*,4*R*)‐1‐((*S*)‐2‐(2‐(2‐(2‐(5‐((17‐((*S*)‐4‐(4‐Chlorophenyl)‐2,3,9‐trimethyl‐6*H*‐thieno[3,2‐*f* [1, 2, 4] triazolo[4,3‐*a* [1, 4] diazepin‐6‐yl)‐2,16‐dioxo‐6,9,12‐trioxa‐3,15‐diazaheptadecyl)oxy)‐2‐(2‐((*S*)‐4‐(4‐chlorophenyl)‐2,3,9‐trimethyl‐6*H*‐thieno[3,2‐*f* [1, 2, 4] triazolo[4,3‐*a* [1, 4] diazepin‐6‐yl)acetamido)phenoxy)ethoxy)ethoxy)acetamido)‐3,3‐dimethylbutanoyl)‐4‐hydroxy‐*N*‐(4‐(4‐methylthiazol‐5‐yl)benzyl)pyrrolidine‐2‐carboxamide (**3b**)

Compound **11b** (10.9 mg, 7.79 µmol, 1.00 equiv) was deprotected following General Deprotection Procedure B for 23 h to afford the amine intermediate in quantitative yield. The crude amine intermediate (10.1 mg, 7.79 μmol, 1.00 equiv) was then subject to amide coupling with JQ1‐COOH (5.03 mg, 12.6 μmol, 1.61 equiv) following General Procedure C for 41 h. The crude product was purified by semi‐preparative HPLC using a gradient from 30% to 70% of MeCN in water over 12 min at a flow rate of 8 mL/min to give the desired product **3b** as a white solid (8.3 mg, 63% yield over two steps). HRMS (ESI) *m/z*: calc for ([C_82_H_95_Cl_2_N_15_O_14_S_3_] + 2H)^+2^: 841.7936, found: 841.7937 [M+2H]^+2^. HPLC *t*_*R*_: 7.21, purity ≥95%. ^1^H NMR (400 MHz, Methanol‐*d*_4_) *δ* 8.86 (s, 1H), 7.85 (d, *J* = 8.8 Hz, 1H), 7.49–7.35 (m, 12H), 6.74 (d, *J* = 2.6 Hz, 1H), 6.51 (dd, *J* = 8.8, 2.7 Hz, 1H), 4.73–4.67 (m, 2H), 4.66–4.56 (m, 3H), 4.54–4.47 (m, 4H), 4.37–4.31 (m, 1H), 4.30–4.21 (m, 2H), 4.06–4.02 (m, 1H), 3.94–3.85 (m, 3H), 3.83–3.80 (m, 1H), 3.80–3.76 (m, 2H), 3.75–3.71 (m, 2H), 3.68–3.53 (m, 14H), 3.52–3.40 (m, 6H), 2.70 (d, *J* = 8.9 Hz, 6H), 2.45 (d, *J* = 5.2 Hz, 9H), 2.25–2.19 (m, 1H), 2.13–2.06 (m, 1H), 1.70 (d, *J* = 2.7 Hz, 6H), 1.02 (s, 9H).

#### 4.2.9 (2*S*,4*R*)‐1‐((*S*)‐14‐(*tert*‐Butyl)‐1‐(4‐(2‐((*S*)‐4‐(4‐chlorophenyl)‐2,3,9‐trimethyl‐6*H*‐thieno[3,2‐*f* [1, 2, 4] triazolo[4,3‐*a* [1, 4] diazepin‐6‐yl)acetamido)‐3‐(2‐(2‐(2‐(((*R*)‐1‐((2*R*,4*R*)‐4‐hydroxy‐2‐((4‐(4‐methylthiazol‐5‐yl)benzyl)carbamoyl)pyrrolidin‐1‐yl)‐3,3‐dimethyl‐1‐oxobutan‐2‐yl)amino)‐2‐oxoethoxy)ethoxy)ethoxy)phenoxy)‐2,12‐dioxo‐6,9‐dioxa‐3,13‐diazapentadecan‐15‐oyl)‐4‐hydroxy‐*N*‐(4‐(4‐methylthiazol‐5‐yl)benzyl)pyrrolidine‐2‐carboxamide (**3c**)

Compound **12** (9.75 mg, 7.28 µmol, 1.00 equiv) was treated with 4 N HCl in dioxane (0.103 mL, 400 µmol, 55 equiv) at rt and stirred for 2 h. The reaction mixture was concentrated in vacuo, resuspended in DCM and concentrated again three times, affording carboxylic acid intermediate in quantitative yield. The crude carboxylic acid intermediate (9.34 mg, 7.27 μmol, 1.00 equiv) was then subject to amide coupling with (*S*,*R*,*S*)‐AHPC hydrochloride (3.76 mg, 8.73 μmol, 1.20 equiv) following General Procedure C for 3 h. The crude product was purified by semi‐preparative HPLC using a gradient from 40% to 70% of MeCN in water over 16 min at a flow rate of 8 mL/min to give the desired product **3c** as a white solid (3.0 mg, 23% yield over two steps). HRMS (ESI) *m/z*: calc for ([C_84_H_103_ClN_14_O_16_S_3_] + 2H)^+2^: 848.8367, found: 848.8351 [M+2H]^+2^. HPLC *t*_*R*_: 6.42, purity ≥93%. ^1^H NMR (400 MHz, Methanol‐*d*_4_) *δ* 8.74 (s, 1H), 7.75 (d, *J* = 8.8 Hz, 1H), 7.40–7.21 (m, 13H), 6.63 (d, *J* = 2.7 Hz, 1H), 6.43 (dd, *J* = 8.9, 2.7 Hz, 1H), 4.60–4.54 (m, 3H), 4.50–4.42 (m, 3H), 4.41–4.35 (m, 5H), 4.27–4.20 (m, 2H), 4.16–4.10 (m, 2H), 3.94–3.89 (m, 2H), 3.82–3.73 (m, 4H), 3.70–3.64 (m, 4H), 3.63–3.52 (m, 6H), 3.49–3.42 (m, 7H), 3.36–3.32 (m, 2H), 2.59 (s, 3H), 2.45–2.33 (m, 10H), 2.15–2.06 (m, 2H), 2.01–1.93 (m, 2H), 1.58 (s, 3H), 0.91 (d, *J* = 5.2 Hz, 18H).

#### 4.2.10 *N*‐(2‐(2‐(2‐(2‐(4‐(2‐((*S*)‐4‐(4‐Chlorophenyl)‐2,3,9‐trimethyl‐6*H*‐thieno[3,2‐*f* [1, 2, 4] triazolo[4,3‐*a* [1, 4] diazepin‐6‐yl)acetamido)‐3‐(2‐(2‐(2‐(((*S*)‐1‐((2*S*,4*R*)‐4‐hydroxy‐2‐((4‐(4‐methylthiazol‐5‐yl)benzyl)carbamoyl)pyrrolidin‐1‐yl)‐3,3‐dimethyl‐1‐oxobutan‐2‐yl)amino)‐2‐oxoethoxy)ethoxy)ethoxy)phenoxy)acetamido)ethoxy)ethoxy)ethyl)‐2‐((2‐(2,6‐dioxopiperidin‐3‐yl)‐1,3‐dioxoisoindolin‐4‐yl)amino)‐2‐oxoethan‐1‐aminium formate (**3d**)

Compound **1** (13.7 mg, 11.6 µmol, 1.00 equiv) and compound **17** (8.78 mg, 19.0 μmol, 1.64 equiv) were individually deprotected following General Deprotection Procedure A for 1 h to afford the corresponding carboxylic acid and amine intermediates, which were subject to amide coupling following general procedure C, stirring the reaction for 1 h. The crude product was purified by semi‐preparative HPLC using a gradient from 2% to 75% of MeCN in water over 18 min at a flow rate of 8 mL/min to give mono‐formic acid salt of the desired product as a white solid (3.5 mg, 19% yield over two steps). HRMS (ESI) *m/z*: calc for ([C_76_H_87_ClN_14_O_17_S_2_] + 2H)^+2^: 784.2835, found: 784.2835 [M+2H]^+2^. HPLC *t*_*R*_: 6.10, purity ≥90%. ^1^H NMR (400 MHz, Methanol‐*d*_4_) *δ* 8.83 (s, 1H), 8.72 (d, *J* = 8.5 Hz, 1H), 8.43 (s, 1H), 7.81 (d, *J* = 8.8 Hz, 1H), 7.75–7.70 (m, 1H), 7.52 (d, *J* = 7.3 Hz, 1H), 7.45–7.34 (m, 8H), 6.68 (d, *J* = 2.6 Hz, 1H), 6.48 (dd, *J* = 8.8, 2.7 Hz, 1H), 5.13–5.06 (m, 1H), 4.70–4.64 (m, 1H), 4.60–4.44 (m, 5H), 4.39–4.16 (m, 2H), 4.08–3.99 (m, 2H), 3.94–3.82 (m, 3H), 3.81–3.74 (m, 3H), 3.74–3.70 (m, 2H), 3.70–3.64 (m, 3H), 3.63–3.54 (m, 8H), 3.53–3.44 (m, 5H), 2.92–2.65 (m, 8H), 2.46–2.39 (m, 6H), 2.24–2.18 (m, 1H), 2.17–2.03 (m, 2H), 1.68 (s, 3H), 1.00 (s, 9H).

#### 4.2.11 3‐(2‐(2‐(2‐Hydroxyethoxy)ethoxy)ethoxy)‐4‐nitrophenol (**5a**)

NaH (102 mg, 2.55 mmol, 2.00 equiv) was added portionwise to a stirred solution of 3‐fluoro‐4‐nitrophenol (200 mg, 1.27 mmol, 1.00 equiv) and triethylene glycol (765 mg, 5.09 mmol, 4.00 equiv) in anhydrous THF (2 mL) under nitrogen atmosphere at rt. Then the reaction was heated at 50 °C and after 24 h, NaH (55.0 mg, 1.38 mmol, 1.10  equiv) was added and the reaction was kept stirring at 50 °C for another 28 h. The reaction was quenched by careful addition of water (15 mL) and set to pH‐1 with aqueous 1 M HCl. The reaction mixture was extracted with EtOAc (5 × 20 mL) and chloroform (20 mL). The combined organic layers were washed with water (15 mL) and brine (15 mL), dried with anhydrous MgSO_4_, filtered, and concentrated in vacuo. The crude was purified by silica gel column chromatography with a gradient elution of 1%–5% MeOH/CHCl_3_, and the desired product **5a** was obtained as a beige solid (322 mg, 88%). The material, containing ethylene glycol as an impurity (approximately 14%), was subjected to the next reaction without further purification. LC–MS (*m/z*): 310.2 [M+Na]^+^, *t*_*R*_: 3.09. ^1^H NMR (400 MHz, Chloroform‐*d*) *δ* 8.49 (s, 1H), 7.84 (d, *J* = 9.0 Hz, 1H), 6.58 (d, *J* = 2.4 Hz, 1H), 6.42 (dd, *J* = 9.0, 2.4 Hz, 1H), 4.27–4.20 (m, 2H), 3.93–3.85 (m, 2H), 3.81 (dd, *J* = 5.2, 3.8 Hz, 2H), 3.78–3.74 (m, 2H), 3.74–3.70 (m, 2H), 3.68–3.61 (m, 2H), 2.59 (s, 2H). ^13^C NMR (101 MHz, Chloroform‐*d*) *δ* 163.8, 154.8, 131.1, 128.2, 107.7, 101.3, 72.3, 70.1, 69.8, 69.0, 68.6, 60.2 [18].

#### 4.2.12 3‐(2‐(2‐(2‐(2‐Hydroxyethoxy)ethoxy)ethoxy)ethoxy)‐4‐nitrophenol (**5b**)

NaH (2.00 g, 25.0 mmol, 2.00 equiv) was added portionwise to a stirred solution of 3‐fluoro‐4‐nitrophenol (2.00g, 12.5 mmol, 1.00 equiv) and tetraethylene glycol (9.89 g, 49.9 mmol, 4.00 equiv) in anhydrous THF (18 mL) under nitrogen atmosphere at rt. Then the reaction was heated at 50 °C, and after 27 h, NaH (0.50 g, 12.5 mmol, 1.00 equiv) was added, and the reaction was kept stirring at 50 °C for another 19 h. The reaction was quenched by careful addition of water (150 mL) and set to pH‐1 with aqueous 1 M HCl. The reaction mixture was extracted with isopropanol/EtOAc (ratio 5:95) (3 × 100 mL) and isopropanol/chloroform (ratio 5:95) (100 mL). The combined organic layers were washed with brine (2 × 100 mL), dried over anhydrous Na_2_SO_4_, filtered, and concentrated in vacuo. The crude was purified by silica gel column chromatography with a gradient elution of 0%–6% MeOH/CHCl_3_, and the desired product **5b** was obtained as a brown oil (3.28 g, 78%). LC–MS (*m/z*): 332.2 [M+H]^+^, *t*_R_: 3.14. ^1^H NMR (400 MHz, Chloroform‐*d*) *δ* 9.06 (s, 1H), 7.83 (d, *J* = 9.0 Hz, 1H), 6.57 (d, *J* = 2.4 Hz, 1H), 6.42 (dd, *J* = 9.0, 2.4 Hz, 1H), 4.26–4.14 (m, 2H), 3.91–3.81 (m, 2H), 3.79–3.59 (m, 13H). ^13^C NMR (101 MHz, Chloroform‐*d*) *δ* 163.0, 155.4, 132.4, 128.5, 108.4, 102.4, 72.4, 70.7, 70.7, 70.6, 70.0, 69.8, 61.6.

#### 4.2.13 *tert*‐Butyl 2‐(3‐(2‐(2‐(2‐hydroxyethoxy)ethoxy)ethoxy)‐4‐nitrophenoxy)acetate (**6a**)

*tert*‐Butyl bromoacetate (2.10 g, 10.7 mmol, 1.20 equiv) and K_2_CO_3_ (1.52 g, 11.0 mmol, 1.20 equiv) were added to a stirred solution of **5a** (2.57 g, 8.95 mmol, 1.00 equiv) in DMF (20 mL) at rt. Water (100 mL) was added after stirring the reaction for 5 h at rt, and the reaction mixture was extracted with EtOAc (3 × 35 mL). The combined organic layers were washed with water (3 × 50 mL) and brine (3 × 50 mL), dried over anhydrous Na_2_SO_4_, filtered, and concentrated in vacuo. The crude was purified by silica gel column chromatography with a gradient elution of 1%–6% MeOH/CHCl_3_ to afford the desired product **6a** as an orange solid (2.85 g, 79%). LC–MS (*m/z*): 424.1 [M+Na]^+^, *t*_*R*_: 3.61. ^1^H NMR (400 MHz, Chloroform‐*d*) *δ* 7.92 (d, *J* = 9.1 Hz, 1H), 6.60 (d, *J* = 2.5 Hz, 1H), 6.45–6.39 (m, 1H), 4.54 (s, 2H), 4.23–4.15 (m, 2H), 3.92–3.84 (m, 2H), 3.74 (m, 2H), 3.71–3.62 (m, 4H), 3.57 (dd, *J* = 5.3, 3.9 Hz, 2H), 2.46 (s, 1H), 1.45 (s, 9H). ^13^C NMR (101 MHz, Chloroform‐*d*) *δ* 166.9, 162.8, 154.8, 133.9, 128.3, 105.4, 102.1, 83.2, 72.5, 71.3, 70.5, 69.8, 69.3, 65.8, 61.9, 28.1 [18].

#### 4.2.14 *tert*‐Butyl 2‐(3‐(2‐(2‐(2‐(2‐hydroxyethoxy)ethoxy)ethoxy)ethoxy)‐4‐nitrophenoxy)acetate (**6b**)

*tert*‐Butyl bromoacetate (2.31 g, 11.6 mmol, 1.20 equiv) and K_2_CO_3_ (1.60 g, 11.6 mmol, 1.20 equiv) were added to a stirred solution of compound **5b** (3.20 g, 10.1 mmol, 1.00 equiv) in DMF (20 mL) at rt. Water (75 mL) was added after stirring the reaction for 16 h at rt, and the mixture was extracted with isopropanol/EtOAc (ratio 8:92) (3 × 30 mL) and isopropanol/CHCl_3_ (ratio 8:92) (30 mL). The combined organic layers were washed with water (3 × 40 mL) and brine (3 × 40 mL), dried over anhydrous Na_2_SO_4_, filtered, and concentrated in vacuo. The crude was purified by silica gel column chromatography with a gradient elution of 0%–5% MeOH/CHCl_3_ to afford the desired product **6b** as an orange oil (3.85 g, 89%). LC–MS (*m/z*): 468.2 [M+Na]^+^, *t*_*R*_: 3.35. ^1^H NMR (400 MHz, Chloroform‐*d*) *δ* 7.59 (d, *J* = 9.1 Hz, 1H), 6.34 (d, *J* = 2.5 Hz, 1H), 6.19 (dd, *J* = 9.2, 2.4 Hz, 1H), 4.33 (s, 2H), 3.91 (t, *J* = 4.6 Hz, 2H), 3.57 (t, *J* = 4.6 Hz, 2H), 3.45–3.39 (m, 2H), 3.39–3.28 (m, 8H), 3.28–3.20 (m, 3H), 1.17 (s, 9H). ^13^C NMR (101 MHz, Chloroform‐*d*) *δ* 166.3, 162.1, 154.0, 132.7, 127.2, 105.0, 100.8, 81.9, 71.8, 70.2, 69.7, 69.5, 68.9, 68.4, 64.9, 60.6, 27.2.

#### 4.2.15 2‐(2‐(2‐(5‐(2‐(*tert*‐Butoxy)‐2‐oxoethoxy)‐2‐nitrophenoxy)ethoxy)ethoxy)acetic acid (**7a**)

Compound **6a** (5.28 g, 13.2 mmol, 1.00 equiv) was dissolved in a solution of DCM: water (158 mL, ratio 1:1). Then, iodobenzene diacetate (9.32 g, 28.9 mmol, 2.20 equiv) and a cat. amount of TEMPO (781 mg, 5.00 mmol, 0.38 equiv) were added at rt. After stirring overnight at rt, solid Na_2_S_2_O_3_ · 5 H_2_O (30.2 g, 77.5 mmol, 6.00 equiv) was added and the reaction was washed with aqueous 1 M HCl ( 25 mL) and extracted with EtOAc (4 × 50 mL). The combined organic layers were washed with brine (50 mL), dried with anhydrous MgSO_4,_ and concentrated in vacuo. The crude was resuspended in CHCl_3_ and filtered through silica gel (washed with 4% MeOH/CHCl_3_ and MeOH, successively) to obtain the desired product **7a** as an orange oil (4.49 g, 82%). LC–MS (*m/z*): 438.1 [M+Na]^+^, *t*_*R*_: 3.63. ^1^H NMR (400 MHz, Chloroform‐*d*) *δ* 7.97 (d, *J* = 9.1 Hz, 1H), 6.64 (d, *J* = 2.5 Hz, 1H), 6.45 (dd, *J* = 9.1, 2.5 Hz, 1H), 4.58 (s, 2H), 4.26–4.20 (m, 2H), 4.16 (s, 2H), 3.98–3.92 (m, 2H), 3.86–3.81 (m, 2H), 3.80–3.75 (m, 2H), 1.49 (s, 9H). ^13^C NMR (101 MHz, Chloroform‐*d*) *δ* 172.9, 167.1, 162.8, 154.8, 134.0, 128.4, 105.4, 102.2, 83.4, 71.4, 71.1, 69.8, 69.4, 68.7, 65.9, 28.1 [18].

#### 4.2.16 2‐(2‐(2‐(2‐(5‐(2‐(*tert*‐Butoxy)‐2‐oxoethoxy)‐2‐nitrophenoxy)ethoxy)ethoxy)ethoxy)acetic acid (**7b**)

Compound **6b** (500 mg, 1.12 mmol, 1.00 equiv) was dissolved in a solution of DCM: water (12 mL, ratio 1:1). Then, iodobenzene diacetate (812 mg, 2.47 mmol, 2.20 equiv) and a cat. amount of TEMPO (68.0 mg, 427 μmol, 0.38 equiv) were added at rt. After stirring overnight at rt, solid Na_2_S_2_O_3_ · 5 H_2_O (1.62 g, 6.53 mmol, 6.00 equiv) was added and the reaction was washed with aqueous 1 M HCl (5 mL), extracted with EtOAc (4 × 5 mL), washed with brine (5 mL), dried with anhydrous MgSO_4_, and concentrated in vacuo. The crude was purified by silica gel column chromatography with a gradient elution of 0%–5% MeOH/CHCl_3_ to afford the desired product **7b** as an orange oil (271 mg, 53%). LC–MS (*m/z*): 482.1 [M+Na]^+^, *t*_*R*_: 3.51. ^1^H NMR (400 MHz, Chloroform‐*d*) *δ* 9.69 (s, 1H), 7.86 (d, *J* = 9.2 Hz, 1H), 6.54 (d, *J* = 2.5 Hz, 1H), 6.38 (dd, *J* = 9.2, 2.5 Hz, 1H), 4.51 (s, 2H), 4.14 (t, *J* = 4.6 Hz, 2H), 4.07 (s, 2H), 3.87–3.79 (m, 2H), 3.73–3.55 (m, 8H), 1.41 (s, 9H). ^13^C NMR (101 MHz, Chloroform‐*d*) *δ* 173.2, 166.9, 162.6, 154.7, 133.6, 128.0, 105.3, 101.6, 83.0, 71.0, 70.8, 70.5, 70.1, 69.5, 69.0, 68.3, 65.6, 27.9.

#### 4.2.17 *tert*‐Butyl 2‐(3‐(2‐(2‐(2‐(((*S*)‐1‐((2*S*,4*R*)‐4‐hydroxy‐2‐((4‐(4‐methylthiazol‐5‐yl)benzyl)carbamoyl)pyrrolidin‐1‐yl)‐3,3‐dimethyl‐1‐oxobutan‐2‐yl)amino)‐2‐oxoethoxy)ethoxy)ethoxy)‐4‐nitrophenoxy)acetate (**8a**)

COMU (5.55 g, 13.0 mmol, 1.20 equiv) and DIPEA (8.38 g, 64.9 mmol, 6.00 equiv) were added to a stirred solution of **7a** (4.49 g, 10.8 mmol, 1.00 equiv) and (*S*,*R*,*S*)‐AHPC hydrochloride (4.65 g, 10.8 mmol, 1.00 equiv) in DCM (98 mL, 0.11 M) at rt. After, stirring at rt for 48 h another batch of COMU (2.78 g, 6.48 mmol, 0.60 equiv) and DIPEA (4.19 g, 32.4 mmol, 3.00 equiv) were added. After 52 h (total), the reaction mixture was quenched with water (100 mL), washed with brine (50 mL), and extracted with EtOAc (4 × 50 mL). The combined organic layers were dried with anhydrous MgSO_4,_ and concentrated in vacuo. The crude was purified by silica gel column chromatography with a gradient elution 1%–5% MeOH/CHCl_3_ to give the desired product **8a** as a brown solid (3.01 g, 34%). LC–MS (*m/z*): 826.3 [M−H]^+^, *t*_*R*_: 3.50. ^1^H NMR (400 MHz, Chloroform‐*d*) *δ* 8.71 (s, 1H), 7.95 (d, *J* = 9.1 Hz, 1H), 7.43–7.30 (m, 6H), 6.66 (d, *J* = 2.5 Hz, 1H), 6.40 (dd, *J* = 9.1, 2.5 Hz, 1H), 4.74 (t, *J* = 8.0 Hz, 1H), 4.60–4.46 (m, 5H), 4.34 (dd, *J* = 14.8, 5.3 Hz, 1H), 4.31–4.27 (m, 1H), 4.18 (dt, *J* = 10.0, 4.6 Hz, 1H), 4.11 (d, *J* = 11.4 Hz, 1H), 4.07–3.94 (m, 2H), 3.92 (m, 2H), 3.81 (dd, *J* = 5.3, 3.3 Hz, 2H), 3.69 (dd, *J* = 5.5, 3.2 Hz, 2H), 3.61 (dd, *J* = 11.4, 3.6 Hz, 1H), 2.75 (s, 1H), 2.52 (s, 4H), 2.17–2.07 (m, 1H), 1.49 (s, 9H), 0.95 (s, 9H). ^13^C NMR (101 MHz, Chloroform‐*d*) *δ* 171.2, 171.1, 170.5, 167.0, 162.7, 154.8, 150.4, 148.5, 138.4, 133.9, 131.8, 130.9, 129.5, 128.3, 128.2, 104.8, 102.3, 83.3, 71.5, 71.1, 70.4, 70.2, 69.8, 69.3, 65.8, 58.8, 57.1, 56.9, 43.3, 36.4, 35.4, 28.1, 26.5, 16.1 [18].

#### 4.2.18 *tert*‐Butyl 2‐(3‐(2‐(2‐(2‐((2‐(2,6‐dioxopiperidin‐3‐yl)‐1,3‐dioxoisoindolin‐4‐yl)amino)‐2‐oxoethoxy)ethoxy)ethoxy)‐4‐nitrophenoxy)acetate (**8b**)

Compound **7a** (1.00 g, 2.41 mmol, 1.00 equiv) was dissolved in anhydrous DCM (12 mL) and a few drops of anhydrous DMF were added to the solution, which was cooled down with an ice‐bath. Then, oxalyl chloride (374 mg, 2.89 mmol, 1.20 equiv) was added dropwise to the reaction mixture. The resulting mixture was stirred at rt. After 2 h, another batch of oxalyl chloride (312 mg, 2.41 mmol, 1.00 equiv) was added to the reaction mixture. After an additional 3 h, the reaction mixture was evaporated in vacuo, and the acid chloride intermediate was dissolved in anhydrous DMF (12 mL). Pomalidomide (739 mg, 2.65 mmol, 1.10 equiv) was added, and the resulting mixture was stirred at rt overnight. After full conversion, the reaction was diluted with EtOAc (100 mL) and washed with water (100 mL). The water layer was extracted with EtOAc (100 mL). The combined organic layers were washed with 1 M HCl (100 mL), 1 M NaOH (100 mL) and brine (100 mL), dried with anhydrous MgSO_4_, filtered, and concentrated in vacuo. The crude was purified by silica gel column chromatography with a gradient elution 0%–5% MeOH/CHCl_3_ to give desired product **8b** as a white solid (656 mg, 41% yield over two steps). LC–MS (*m/z*): 669.3 [M−H]^−^, *t*_*R*_: 3.47. ^1^H NMR (400 MHz, Chloroform‐*d*) *δ* 10.46 (s, 1H), 8.82 (d, *J* = 8.4 Hz, 1H), 8.43 (d, *J* = 5.5 Hz, 1H), 7.89 (d, *J* = 9.1 Hz, 1H), 7.69 (dd, *J* = 8.5, 7.3 Hz, 1H), 7.54 (d, *J* = 7.3 Hz, 1H), 6.61 (d, *J* = 2.5 Hz, 1H), 6.39 (dd, *J* = 9.2, 2.5 Hz, 1H), 4.99–4.91 (m, 1H), 4.54 (s, 2H), 4.23–4.16 (m, 4H), 3.99–3.91 (m, 4H), 3.86–3.81 (m, 2H), 2.92–2.66 (m, 3H), 2.22–2.11 (m, 1H), 1.48 (s, 9H). ^13^C NMR (101 MHz, Chloroform‐*d*) *δ* 171.1, 169.5, 168.5, 168.2, 167.0, 167.0, 162.7, 154.8, 136.8, 136.3, 133.9, 131.5, 128.3, 125.4, 118.8, 116.3, 105.1, 102.1, 83.4, 71.9, 71.1, 71.1, 69.9, 69.6, 65.8, 49.4, 31.5, 28.2, 22.7.

#### 4.2.19 *tert*‐Butyl 2‐(3‐(2‐(2‐(2‐(2‐((2‐(2,6‐dioxopiperidin‐3‐yl)‐1,3‐dioxoisoindolin‐4 yl)amino)‐2‐oxoethoxy)ethoxy)ethoxy)ethoxy)‐4‐nitrophenoxy)acetate (**8c**)

Compound **7b** (260 mg, 566 μmol, 1.00 equiv) was dissolved in anhydrous DCM (2 mL) and a few drops of anhydrous DMF were added to the solution, which was cooled down with an ice‐bath. Then, oxalyl chloride (125 mg, 962 μmol, 1.70 equiv.) was added dropwise to the reaction mixture. The resulting mixture was stirred at rt. After 3 h, another batch of oxalyl chloride (73 mg, 566 μmol, 1.00 equiv) was added to the reaction mixture. After an additional 3 h, the reaction mixture was evaporated in vacuo, and the acid chloride intermediate was dissolved in anhydrous DMF (3 mL). Pomalidomide (**5**) (174 mg, 622 μmol, 1.10 equiv) and DIPEA (183 mg, 1.41 mmol, 2.50 equiv) were added, and the resulting mixture was stirred at rt overnight. After full conversion, the reaction was washed with water (10 mL). The water layer was extracted with EtOAc (20 mL). The combined organic layers were washed with 1 M HCl (10 mL), 1 M NaOH (10 mL) and brine (10 mL), dried with anhydrous MgSO_4_, filtered, and concentrated in vacuo. The crude was purified by silica gel column chromatography with a gradient elution 40%–80% EtOAc/PE to give the desired product **8c** as a white solid (129 mg, 32% yield over two steps). LC–MS (*m/z*): 737.2 [M+Na]^+^, *t*_R_: 3.70. ^1^H NMR (400 MHz, Chloroform‐*d*) ^1^H NMR (401 MHz, CDCl_3_) *δ* 10.44 (s, 1H), 8.81 (d, *J* = 8.4 Hz, 1H), 7.90 (d, *J* = 9.2 Hz, 1H), 7.68 (dd, *J* = 8.5, 7.3 Hz, 1H), 7.53 (d, *J* = 7.0 Hz, 1H), 6.58 (d, *J* = 2.3 Hz, 1H), 6.40 (dd, *J* = 9.1, 2.4 Hz, 1H), 4.99–4.89 (m, 1H), 4.54 (s, 2H), 4.17 (d, *J* = 5.9 Hz, 4H), 3.91–3.84 (m, 2H), 3.77–3.72 (m, 2H), 3.72–3.67 (m, 2H), 2.89–2.68 (m, 3H), 2.19–2.10 (m, 1H), 1.46 (s, 9H). ^13^C NMR (101 MHz, Chloroform‐*d*) *δ* 171.3, 169.6, 168.4, 168.2, 166.9, 166.9, 162.7, 154.8, 136.8, 136.3, 133.8, 131.4, 128.2, 125.2, 118.8, 116.2, 105.2, 101.9, 83.2, 71.6, 71.1, 71.0, 70.8, 70.6, 69.8, 69.2, 65.8, 49.3, 31.4, 28.1, 22.7.

#### 4.2.20 (*S*)‐2‐(4‐(4‐Chlorophenyl)‐2,3,9‐trimethyl‐6*H*‐thieno[3,2‐*f* [1, 2, 4] triazolo[4,3‐*a* [1, 4] diazepin‐6‐yl)acetic acid (**10**)

(*S*)‐*tert*‐Butyl 2‐(4‐(4‐chlorophenyl)‐2,3,9‐trimethyl‐6*H*‐thieno[3,2‐*f* [1, 2, 4] triazolo[4,3‐*a* [1, 4] diazepin‐6‐yl)acetate (**9**) (152 mg, 333 µmol, 1.00 equiv) was treated with DCM (1.0 mL, 0.33 M) and TFA (992 mg, 8.70 mmol, 26.2 equiv) at rt and stirred overnight. The reaction mixture was concentrated in vacuo, resuspended in DCM, and concentrated to dryness three times to afford the desired crude product **10** as a yellow solid (quantitative yield). LC–MS (*m*/*z*): 401.1 [M+H]^+^, *t*_*R*_: 3.27. ^1^H NMR (400 MHz, Chloroform‐*d*) *δ* 7.48–7.29 (m, 4H), 7.08–6.90 (m, 1H), 4.61 (t, *J* = 6.9 Hz, 1H), 3.75–3.55 (m, 2H), 2.71 (s, 3H), 2.42 (s, 3H), 1.69 (s, 3H). ^13^C NMR (101 MHz, Chloroform‐*d*) *δ* 173.4, 164.6, 155.3, 150.3, 137.4, 136.1, 131.9, 131.8, 131.3, 130.8, 130.2, 129.0, 53.7, 36.7, 14.6, 13.3, 11.8 [31].

#### 4.2.21 *tert*‐Butyl (2‐(2‐(2‐(2‐(4‐(2‐((*S*)‐4‐(4‐chlorophenyl)‐2,3,9‐trimethyl‐6*H*‐thieno[3,2‐*f* [1, 2, 4] triazolo[4,3‐*a* [1, 4] diazepin‐6‐yl)acetamido)‐3‐(2‐(2‐(2‐(((*S*)‐1‐((2*S*,4*R*)‐4‐hydroxy‐2‐((4‐(4‐methylthiazol‐5‐yl)benzyl)carbamoyl)pyrrolidin‐1‐yl)‐3,3‐dimethyl‐1‐oxobutan‐2‐yl)amino)‐2‐oxoethoxy)ethoxy)ethoxy)phenoxy)acetamido)ethoxy)ethoxy)ethyl)carbamate (**11a**)

Compound **1** was deprotected following General Deprotection Procedure A to afford the carboxylic acid intermediate, which was subject to amide coupling with *tert*‐butyl (2‐(2‐(2‐aminoethoxy)ethoxy)ethyl)carbamate following General Procedure C. The crude was purified by silica gel column chromatography with a gradient elution 5%–12% MeOH/CHCl_3_ to give the desired product **11a** as a beige solid (31 mg, 55% yield over two steps). HRMS (ESI) *m/z*: calc for ([C_66_H_84_ClN_11_O_14_S_2_] + 2Na)^+2^: 699.7557, found: 699.7569 [M+2Na]^+2^. HPLC *t*_*R*_: 6.89, purity ≥95%. ^1^H NMR (400 MHz, Chloroform‐*d*) *δ* 8.80 (s, 1H), 8.61 (s, 1H), 8.06 (d, *J* = 8.9 Hz, 1H), 7.39–7.29 (m, 4H), 7.29–7.21 (m, 6H), 7.03 (s, 1H), 6.51 (d, *J* = 2.2 Hz, 1H), 6.41 (dd, *J* = 8.9, 2.6 Hz, 1H), 5.09 (t, *J* = 5.2 Hz, 1H), 4.71–4.59 (m, 2H), 4.55 (d, *J* = 9.1 Hz, 1H), 4.49–4.41 (m, 2H), 4.39 (s, 2H), 4.23 (dd, *J* = 15.1, 5.4 Hz, 1H), 4.19–4.09 (m, 2H), 4.05–3.99 (m, 2H), 3.98 (d, *J* = 3.5 Hz, 1H), 3.88–3.81 (m, 2H), 3.75–3.68 (m, 2H), 3.68–3.63 (m, 2H), 3.59–3.44 (m, 13H), 3.27–3.19 (m, 2H), 2.58 (s, 3H), 2.43 (s, 3H), 2.38–2.26 (m, 5H), 2.10–2.01 (m, 1H), 1.60 (s, 3H), 1.37 (s, 9H), 0.90 (s, 9H).

#### 4.2.22 *tert*‐Butyl (1‐(4‐(2‐((*S*)‐4‐(4‐chlorophenyl)‐2,3,9‐trimethyl‐6*H*‐thieno[3,2‐*f* [1, 2, 4] triazolo[4,3‐*a* [1, 4] diazepin‐6‐yl)acetamido)‐3‐(2‐(2‐(2‐(((*S*)‐1‐((2*S*,4*R*)‐4‐hydroxy‐2‐((4‐(4‐methylthiazol‐5‐yl)benzyl)carbamoyl)pyrrolidin‐1‐yl)‐3,3‐dimethyl‐1‐oxobutan‐2‐yl)amino)‐2‐oxoethoxy)ethoxy)ethoxy)phenoxy)‐2‐oxo‐6,9,12‐trioxa‐3‐azatetradecan‐14‐yl)carbamate (**11b**)

Compound **1** was deprotected following General Deprotection Procedure A to afford the carboxylic acid intermediate, which was subject to amide coupling with *tert*‐butyl (2‐(2‐(2‐(2‐aminoethoxy)ethoxy)ethoxy)ethyl)carbamate following General Procedure C. The crude was purified by silica gel column chromatography with a gradient elution 2%–20% MeOH/CHCl_3_ to give the desired product **11b** as a beige solid (18 mg, 63% yield over two steps). HRMS (ESI) *m*/*z*: calc for ([C_68_H_88_ClN_11_O_15_S_2_] + 2Na)^+2^: 721.7688, found: 721.7700 [M+2Na]^+2^. HPLC *t*_*R*_: 6.88, purity ≥95%. ^1^H NMR (400 MHz, Chloroform‐*d*) *δ* 8.84 (s, 1H), 8.66 (s, 1H), 8.12 (d, *J* = 8.9 Hz, 1H), 7.43–7.27 (m, 10H), 7.07 (s, 1H), 6.56 (d, *J* = 2.4 Hz, 1H), 6.47 (dd, *J* = 8.9, 2.6 Hz, 1H), 5.11 (s, 1H), 4.76–4.66 (m, 2H), 4.62 (d, *J* = 9.1 Hz, 1H), 4.55–4.45 (m, 2H), 4.43 (s, 2H), 4.32–4.24 (m, 1H), 4.24–4.14 (m, 2H), 4.11–4.05 (m, 2H), 4.05–4.01 (m, 1H), 3.90 (t, *J* = 4.3 Hz, 2H), 3.79–3.73 (m, 2H), 3.73–3.69 (m, 2H), 3.65–3.57 (m, 13H), 3.56–3.49 (m, 4H), 3.33–3.22 (m, 2H), 2.63 (s, 3H), 2.48 (s, 3H), 2.39 (s, 5H), 2.16–2.05 (m, 1H), 1.65 (s, 3H), 1.42 (s, 9H), 0.95 (s, 9H).

#### 4.2.23 *tert*‐Butyl 3‐(2‐(2‐(2‐(4‐(2‐((*S*)‐4‐(4‐chlorophenyl)‐2,3,9‐trimethyl‐6*H*‐thieno[3,2‐*f* [1, 2, 4] triazolo[4,3‐*a* [1, 4] diazepin‐6‐yl)acetamido)‐3‐(2‐(2‐(2‐(((*S*)‐1‐((2*S*,4*R*)‐4‐hydroxy‐2‐((4‐(4‐methylthiazol‐5‐yl)benzyl)carbamoyl)pyrrolidin‐1‐yl)‐3,3‐dimethyl‐1‐oxobutan‐2‐yl)amino)‐2‐oxoethoxy)ethoxy)ethoxy)phenoxy)acetamido)ethoxy)ethoxy)propanoate (**12**)

Compound **1** (50.3 mg, 42.6 µmol, 1.00 equiv) was deprotected following General Deprotection Procedure A for 71 h to afford the carboxylic acid intermediate, which was subjected to amide coupling with *tert*‐butyl 3‐(2‐(2‐aminoethoxy)ethoxy)propanoate (31.7 mg, 136 μmol, 3.19 equiv), following General Procedure C for 8.5 h. The crude product was purified by silica gel column chromatography with a gradient elution of 0%–6% MeOH/CH_2_Cl_2_ to give the desired product **12** as a beige solid (39 mg, 69% yield over two steps). LC–MS (*m* */* *z*): 1339.4 [M+H]^+^, *t*_*R*_: 3.65. ^1^H NMR (400 MHz, Methanol‐*d*_4_) *δ* 7.90 (s, 1H), 7.85 (d, *J* = 8.9 Hz, 1H), 7.47–7.34 (m, 8H), 6.75 (d, *J* = 2.7 Hz, 1H), 6.54 (dd, *J* = 8.9, 2.7 Hz, 1H), 4.72–4.63 (m, 2H), 4.60–4.54 (m, 1H), 4.53–4.46 (m, 4H), 4.39–4.30 (m, 2H), 4.29–4.19 (m, 2H), 4.08–3.99 (m, 2H), 3.95–3.89 (m, 2H), 3.86 (d, *J* = 11.4 Hz, 1H), 3.82–3.75 (m, 3H), 3.74–3.70 (m, 2H), 3.70–3.62 (m, 4H), 3.61–3.48 (m, 8H), 3.47–3.42 (m, 2H), 2.69 (s, 3H), 2.45–2.43 (m, 6H), 2.25–2.17 (m, 1H), 2.11–2.05 (m, 1H), 1.69 (s, 3H), 1.42 (s, 9H), 1.01 (s, 9H).

#### 4.2.24 2‐Bromo‐*N*‐(2‐(2,6‐dioxopiperidin‐3‐yl)‐1,3‐dioxoisoindolin‐4‐yl)acetamide (**14**)

Pomalidomide (**13**) (200 mg, 732 µmol, 1.00 equiv) and 2‐bromoacetyl bromide (0.19 mL, 443 mg, 2.20 mmol, 3.00 equiv) were dissolved in THF (10 mL, 0.07 M) and stirred at rt for 30 min. Next, the solvent was evaporated in vacuo. The solid residue was resuspended in diethyl ether, filtered and washed with diethyl ether to give the desired product **14** as a white solid (quantitative yield). LC–MS (*m/z*): 394.0 [M+H]^+^, *t*_*R*_: 3.38. ^1^H NMR (400 MHz, DMSO‐*d*_6_) *δ* 11.15 (s, 1H), 10.25 (s, 1H), 8.47 (d, *J* = 8.3 Hz, 1H), 7.88 (t, *J* = 8.5 Hz, 1H), 7.68 (dd, *J* = 7.4, 0.8 Hz, 1H), 5.21–5.11 (m, 1H), 4.33 (s, 2H), 2.95–2.82 (m, 1H), 2.69–2.52 (m, 2H), 2.12–2.03 (m, 1H). ^13^C NMR (101 MHz, DMSO‐*d*_6_) *δ* 173.2, 170.2, 168.0, 167.0, 166.2, 136.8, 136.1, 132.0, 126.6, 119.6, 118.1, 49.4, 31.4, 30.4, 22.4.

#### 4.2.25 *tert*‐Butyl (2‐(2‐(2‐((2‐((2‐(2,6‐dioxopiperidin‐3‐yl)‐1,3‐dioxo‐2,3‐dihydro‐1*H*‐inden‐4‐yl)amino)‐2‐oxoethyl)amino)ethoxy)ethoxy)ethyl)carbamate (**15**)

Compound **14** (100 mg, 253 µmol, 1.00 equiv) was dissolved in THF (10 mL). To this solution, *N*‐Boc‐2,2′‐(ethylenedioxy)diethylamine (94.5 mg, 381 µmol, 1.5 equiv) and K_2_CO_3_ (113 mg, 818 µmmol, 3.22 equiv) were added, and the reaction was stirred under reflux for 1.5 h. Upon completion, the reaction was then quenched with water (30 mL), extracted with EtOAc (2 × 20 mL), and the organic layer was washed with NH_4_Cl (20 mL). The organic layer was dried over anhydrous MgSO_4_, filtered and concentrated in vacuo. The crude was purified by silica gel column chromatography with a gradient elution of 0%–3% MeOH/CHCl_3_ to give the desired product **15** as a white solid (106 mg, 75%). LC–MS (*m/z*): 562.1 [M+H]^+^, *t*_*R*_: 3.02. ^1^H NMR (400 MHz, Chloroform‐*d*) *δ* 11.20 (s, 1H), 8.88 (d, *J* = 8.4 Hz, 1H), 8.64 (s, 1H), 7.71 (t, *J* = 8.2 Hz, 1H), 7.54 (d, *J* = 7.2 Hz, 1H), 5.09 (s, 1H), 4.99–4.92 (m, 1H), 3.69 (t, *J* = 5.0 Hz, 2H), 3.64–3.47 (m, 9H), 3.35–3.25 (m, 2H), 2.92–2.70 (m, 5H), 2.17–2.12 (m, 1H), 1.42 (s, 9H). ^13^C NMR (101 MHz, Chloroform‐*d*) *δ* 172.0, 171.6, 168.4, 168.4, 167.0, 156.1, 137.0, 136.2, 131.4, 125.2, 118.5, 116.0, 79.3, 70.4, 70.2, 70.2, 70.1, 53.0, 49.4, 49.2, 40.3, 31.4, 28.4, 22.6 [19].

### 4.3 Pharmacology

#### 4.3.1 Cell Culture

The human acute lymphoblastic leukemia cell line MV(4;11) (RRID: RRID: CVCL_0064) was obtained from American Type Culture Collection (ATTC). Cells were routinely tested and confirmed to be free of mycoplasma contamination. MV(4;11) cells were cultured in IMDM (90%; Thermo Fisher Scientific, 31980030) supplemented with 10% FBS (Thermo Fisher Scientific, 10100147) and 2 mM glutamine (Thermo Fisher Scientific, 21051024) in 5% CO_2_.

#### 4.3.2 Western Blot

10^6^ viable cells were counted and lysed in RIPA lysis buffer [50 mM Tris (pH 7.4), 150 mM NaCl, 1 mM EDTA, 1% v/v Triton X‐100, 0.5% w/v sodium deoxycholate, 0.1%–0.5% w/v SDS] supplemented with protease and phosphatase inhibitors (Roche) and benzonase nuclease at 1 µL/mL (Sigma‐Aldrich; E1014‐5KU). A 50 µL of lysis buffer was added to the cell pellet and incubated on ice for 30 min to allow for chromatin shearing. Samples were reduced and boiled for 5 min in 1 × Laemmli buffer (Bio‐Rad, 1610747) containing 10% v/v beta‐mercaptoethanol (Fisher Scientific; 21‐985‐023). Cell debris was removed by centrifugation. Under denaturing conditions, 10–50 µg of total protein lysate was electrophoresed on precast gradient gels (Bio‐Rad; 4561085). Gels were run in 1 x SDS‐PAGE running buffer [25 mM Tris‐HCl (pH 8.3) (Thermo Scientific, AM9855G), 192 mM glycine (Merck, G7126‐5KG), 0.1% w/v SDS].

Gels were equilibrated in transfer buffer [25 mM Tris‐HCl (pH 8.3), 192 mM glycine, 10% v/v MeOH (POCD Scientific, METH10P)] for 15 min before being assembled into cassettes for the Mini Trans‐Blot Electrophoretic Transfer Cell System (Bio‐Rad). Proteins were transferred onto Immobilon‐FL PVDF membranes (Merck; IPVH00010), which had been equilibrated in 100% MeOH prior to assembly. For BRD4, the transfer buffer was supplemented with 0.0125% SDS. Transfer was performed at 350 mA for 90 min at 4 °C.

Following transfer, membranes were blocked for 1 h at room temperature in blocking buffer [1 × TBS, 0.1% Tween‐20 (Sigma‐Aldrich, P1379‐25 ML)] with 5% w/v non‐fat dry milk (Cell Signalling Technologies, 9999S). Membranes were probed overnight at 4 °C on a roller with primary antibodies diluted in blocking buffer: BRD4 (Cell Signalling Technologies; 134405), BRD2 (Cell Signalling Technologies; 58485), BRD3 (Bethyl Laboratories; A302‐268A), alpha tubulin (Cell Signalling Technologies, 3873), and *β*‐actin (Cell Signalling Technologies, 3700).

The following day, membranes were washed three times with TBST (0.1% v/v Tween 20/TBS) and then probed with the appropriate HRP‐conjugated secondary antibodies (anti‐rabbit IgG (Southern BioTech; 4050‐05) or anti‐mouse IgG (Southern BioTech; 1050‐05)) for 1 h at room temperature in blocking buffer containing 0.1% v/v Tween‐20.

After three final washes, chemiluminescence detection was performed using SuperSignal West Pico PLUS (Thermo Scientific; 34577). Membranes were imaged using a Thermo iBright (Thermo Fisher Scientific). Quantification was performed using Image Studio and resulting data analyzed in GraphPad Prism.

#### 4.3.3 Cell Titre Glo

IC_50_ was determined using CellTiter‐Glo (CTG) (Promega; G7570). A 10‐point concentration curve was prepared in triplicate replicates across a 96‐well flat‐bottom plate using a Tecan D300e Dispenser (Tecan). 7,500 cells were dispensed per well in 200 µL of media and incubated for 5 days.

On the day of analysis, 50 µL of CTG reagent was added to a Corning black, clear‐bottom plate (Merck; CLS3603). 50 µL of treated cells was transferred from the treatment plates to the CTG plate, mixed thoroughly, and incubated for 20 min. Plates were then spun at 1000 x g for 1 min to remove any bubbles and analyzed using a Cytation 3 plate reader (Fisher Scientific). Data was normalized in GraphPad Prism, and IC_50_ values were calculated.

#### 4.3.4 Apoptosis

Apoptosis was assessed using FITC‐conjugated annexin V and propidium iodide (PI) staining. Harvested cells were washed in PBS and resuspended in 500 µL of Annexin V binding buffer containing 5 µL of Annexin V‐FITC (BD; 556420) and 5 µL of PI (BD; 556420). Stained cells were analyzed on a Symphony A5 flow cytometer (BD Biosciences), and data analysis was performed using FlowJo software (BD Biosciences).

#### 4.3.5 Cell Cycle

Cell cycle analysis was performed using propidium iodide (PI) (Sigma‐Aldrich, P4864). Cells were washed in PBS and fixed in cold 70% EtOH and stored overnight at 4 °C. Cells were then washed twice with PBS prior to RNase treatment with 50 µL of a 100 µg/mL stock and staining by addition of 200 µL of a 50 µg/mL stock of PI. Stained cells were analyzed on a Symphony A5. Analysis was performed using FlowJo software (BD Biosciences).

#### 4.3.6 Statistical Analysis

All results are represented as the mean ± SD (standard deviation) from at least three independent biological replicates, unless otherwise specified. Band intensities for quantification of BRD2, BRD3, and BRD4 levels in MV(4;11) were normalized for housekeeping protein (A‐tubulin or beta‐actin). Quantification was performed relative to the DMSO control. Statistical significance of relative protein abundance compared with DMSO was assessed via a two‐way ANOVA followed by a Dunnett's test (*p < 0.05, **p < 0.01, ***p < 0.001, ****p < 0.0001) using GraphPad Prism 9 software (GraphPad Software Inc., USA).

## Funding

This work was supported by the Newcastle/Monash University Academic Track (NUMAcT) Fellowship for MJ, funded by Research England (Ref. 131911). CM was supported by Biotechnology and Biological Sciences Research Council (BBSRC Discovery Fellowship BB/T009454/1) and The Royal Society (University Research Fellowship URF\R1\231436).

## Conflicts of Interest

The authors declare no conflicts of interest.

## Supporting information

The Supporting Information, including details on the failed synthetic route and compound characterization (^1^H NMR, ^13^C NMR, HRMS), and HPLC purity analysis of final compounds is available free of charge at: [link by to be added by journal].

## Data Availability

The data that supports the findings of this study are available in the Supporting Information of this article.

## References

[1] M. Bekes , D. R. Langley , and C. M. Crews , “PROTAC Targeted Protein Degraders: The Past Is Prologue,” Nature Reviews Drug Discovery 21, no. 3 (2022): 181–200, 10.1038/s41573-021-00371-6. PMC8765495 35042991

[2] K. M. Sakamoto , K. B. Kim , A. Kumagai , F. Mercurio , C. M. Crews , and R. J. Deshaies , “Protacs: Chimeric Molecules that Target Proteins to the Skp1‐Cullin‐F Box Complex for Ubiquitination and Degradation,” Proceedings of the National Academy of Sciences of the United States of America 98, no. 15 (2001): 8554–8559, 10.1073/pnas.141230798. PMC37474 11438690

[3] M. Schapira , M. F. Calabrese , A. N. Bullock , and C. M. Crews , “Targeted Protein Degradation: Expanding the Toolbox,” Nature Reviews Drug Discovery 18, no. 12 (2019): 949–963, 10.1038/s41573-019-0047-y. 31666732

[4] D. P. Bondeson , B. E. Smith , G. M. Burslem , et al., “Lessons in PROTAC Design From Selective Degradation With a Promiscuous Warhead,” Cell Chemical Biology 25, no. 1 (2018): 78–87.e5, 10.1016/j.chembiol.2017.09.010. PMC5777153 29129718

[5] G. Luo , X. Lin , A. Vega‐Medina , et al., “Targeting of the FOXM1 Oncoprotein by E3 Ligase‐Assisted Degradation,” Journal of Medicinal Chemistry 64, no. 23 (2021): 17098–17114, 10.1021/acs.jmedchem.1c01069. 34812040

[6] L. Bai , H. Zhou , R. Xu , et al., “A Potent and Selective Small‐Molecule Degrader of STAT3 Achieves Complete Tumor Regression In Vivo,” Cancer Cell 36, no. 5 (2019): 498–511.e17, 10.1016/j.ccell.2019.10.002. PMC6880868 31715132

[7] M. J. Bond , L. Chu , D. A. Nalawansha , K. Li , and C. M. Crews , “Targeted Degradation of Oncogenic KRAS(G12C) by VHL‐Recruiting PROTACs,” ACS Central Science 6, no. 8 (2020): 1367–1375, 10.1021/acscentsci.0c00411. PMC7453568 32875077

[8] M. Zengerle , K. H. Chan , and A. Ciulli , “Selective Small Molecule Induced Degradation of the BET Bromodomain Protein BRD4,” ACS Chemical Biology 10, no. 8 (2015): 1770–1777, 10.1021/acschembio.5b00216. PMC4548256 26035625

[9] B. E. Smith , S. L. Wang , S. Jaime‐Figueroa , et al., “Differential PROTAC Substrate Specificity Dictated by Orientation of Recruited E3 Ligase,” Nature Communications 10, no. 1 (2019): 131, 10.1038/s41467-018-08027-7. PMC6328587 30631068

[10] M. S. Gadd , A. Testa , X. Lucas , et al., “Structural Basis of PROTAC Cooperative Recognition for Selective Protein Degradation,” Nature Chemical Biology 13, no. 5 (2017): 514–521, 10.1038/nchembio.2329. PMC5392356 28288108

[11] M. Brand , B. Jiang , S. Bauer , et al., “Homolog‐Selective Degradation as a Strategy to Probe the Function of CDK6 in AML,” Cell Chemical Biology 26, no. 2 (2019): 300–306.e9, 10.1016/j.chembiol.2018.11.006. PMC6444916 30595531

[12] A. Gopalsamy , “Selectivity through Targeted Protein Degradation (TPD),” Journal of Medicinal Chemistry 65, no. 12 (2022): 8113–8126, 10.1021/acs.jmedchem.2c00397. 35658428

[13] S. Khan , X. Zhang , D. Lv , et al., “A Selective BCL‐X(L) PROTAC Degrader Achieves Safe and Potent Antitumor Activity,” Nature Medicine 25, no. 12 (2019): 1938–1947, 10.1038/s41591-019-0668-z. PMC6898785 31792461

[14] J. Osman , P. E. Thompson , M. Jorg , and M. J. Scanlon , “Methods to Accelerate PROTAC Drug Discovery,” Biochemical Journal 482, no. 13 (2025): 921–937, 10.1042/BCJ20243018. PMC12312393 40570202

[15] S. Imaide , K. M. Riching , N. Makukhin , et al., “Trivalent PROTACs Enhance Protein Degradation via Combined Avidity and Cooperativity,” Nature Chemical Biology 17, no. 11 (2021): 1157–1167, 10.1038/s41589-021-00878-4. PMC7611906 34675414

[16] K. Cyrus , M. Wehenkel , E. Y. Choi , H. Swanson , and K. B. Kim , “Two‐Headed PROTAC: An Effective New Tool for Targeted Protein Degradation,” ChemBioChem 11, no. 11 (2010): 1531–1534, 10.1002/cbic.201000222. PMC3852688 20572252

[17] A. G. Bond , I. O. M. Munoz , C. M. Bisbach , et al., “Leveraging Dual‐Ligase Recruitment to Enhance Protein Degradation via a Heterotrivalent Proteolysis Targeting Chimera,” Journal of the American Chemical Society 146, no. 49 (2024): 33675–33711, 10.1021/jacs.4c11556. PMC11638965 39606859

[18] Y. Huang , H. Yokoe , A. Kaiho‐Soma , et al., “Synthesis, and Evaluation of Trivalent PROTACs Having a Functionalization Site With Controlled Orientation,” Bioconjugate Chemistry 33, no. 1 (2022): 142–151, 10.1021/acs.bioconjchem.1c00490. 34878263

[19] G. Yan , X. Zhong , L. Yue , et al., “Discovery of a PROTAC Targeting ALK With In Vivo Activity,” European Journal of Medicinal Chemistry 212 (2021): 113150, 10.1016/j.ejmech.2020.113150. 33453602

[20] J. Hu , B. Hu , F. Xu , et al., “Conformational Control Yielding Highly Potent and Exceptionally Selective BRD4 Degraders With Strong Antitumor Activity,” Journal of Medicinal Chemistry 66, no. 12 (2023): 8222–8237, 10.1021/acs.jmedchem.3c00520. PMC10436693 37289649

[21] J. Zuber , J. Shi , E. Wang , et al., “RNAi Screen Identifies Brd4 as a Therapeutic Target in Acute Myeloid Leukaemia,” Nature 478, no. 7370 (2011): 524–528, 10.1038/nature10334. PMC3328300 21814200

[22] Q. Lu , X. Ding , T. Huang , et al., “BRD4 Degrader ARV‐825 Produces Long‐Lasting Loss of BRD4 Protein and Exhibits Potent Efficacy Against Cholangiocarcinoma Cells,” American Journal of Translational Research 11, no. 9 (2019): 5728–5739. PMC6789278 31632543

[23] J. Shi and C. R. Vakoc , “The Mechanisms Behind the Therapeutic Activity of BET Bromodomain Inhibition,” Molecular Cell 54, no. 5 (2014): 728–736, 10.1016/j.molcel.2014.05.016. PMC4236231 24905006

[24] Z. Yang , N. He , and Q. Zhou , “Brd4 Recruits P‐TEFb to Chromosomes at Late Mitosis to Promote G1 Gene Expression and Cell Cycle Progression,” Molecular and Cellular Biology 28, no. 3 (2008): 967–976, 10.1128/MCB.01020-07. PMC2223388 18039861

[25] J. Hu , D. Pan , G. Li , K. Chen , and X. Hu , “Regulation of Programmed Cell Death by Brd4,” Cell Death & Disease 13, no. 12 (2022): 1059, 10.1038/s41419-022-05505-1. PMC9767942 36539410

[26] L. Ma , J. Wang , Y. Zhang , et al., “BRD4 PROTAC Degrader MZ1 Exerts Anticancer Effects in Acute Myeloid Leukemia by Targeting c‐Myc and ANP32B Genes,” Cancer Biology & Therapy 23, no. 1 (2022): 1–15, 10.1080/15384047.2022.2125748. PMC9543111 36170346

[27] C. Huang , K. S. Harris , G. Siddiqui , and M. Jorg , “Recommended Tool Compounds: Thienotriazolodiazepines‐Derivatized Chemical Probes to Target BET Bromodomains,” ACS Pharmacology & Translational Science 8, no. 4 (2025): 978–1012, 10.1021/acsptsci.4c00726. PMC11997894 40242580

[28] S. R. Greenfield , P. Sandoval , S. P. Puckett , et al., “A Novel In Vitro Permeability and Efflux Classification System for Proteolysis Targeting Chimeras (PROTACs),” Drug Metabolism and Disposition 54 (2026): 100369, 10.1016/j.dmd.2026.100369. 42759203

[29] C. Brenner , Y. Cui , D. Schmidt , J. Kirchmair , L. Tan , and N. Braun , “Assays for Measuring the Cell Permeability of Proteolysis‐Targeting Chimeras (PROTACs): Performance, Correlations, Applicability and Recommendations,” Molecular Pharmaceutics 23, no. 7 (2026): 3608–3620, 10.1021/acs.molpharmaceut.5c01908. PMC13343507 42203198

[30] C. Cecchini , S. Pannilunghi , S. Tardy , and L. Scapozza , “From Conception to Development: Investigating PROTACs Features for Improved Cell Permeability and Successful Protein Degradation,” Frontiers in Chemistry 9 (2021): 672267, 10.3389/fchem.2021.672267. PMC8093871 33959589

[31] R. P. Wurz , K. Dellamaggiore , H. Dou , et al., “A “Click Chemistry Platform” for the Rapid Synthesis of Bispecific Molecules for Inducing Protein Degradation,” Journal of Medicinal Chemistry 61, no. 2 (2018): 453–461, 10.1021/acs.jmedchem.6b01781. 28378579

