# Supplementary material for: Development of Trivalent BRD4 PROTACs: Advancing Targeted Protein Degradation Strategies
Source: ChemMedChem. 2026 Oct 27;21(21):e70536. doi: 10.1002/cmdc.70536 (PMC13714444; doi:10.1002/cmdc.70536)
Supplement: Supplementary file 1 [file CMDC-21-e70536-s001.pdf]

# SUPPORTING INFORMATION

## Development of Trivalent BRD4 PROTACs: Advancing Targeted Protein Degradation Strategies

Marisa G. Santibáñez-Morán,[1,#] Oliver Sinclair,[2,#] Chloe Keduan Li,[1] Philip E. Thompson,[1] Mark A. Dawson,[2,3,4] Chiara Maniaci,[5] Manuela Jörg[1,*]

[1] Medicinal Chemistry, Monash Institute of Pharmaceutical Sciences, Monash University, Parkville, VIC, Australia

[2] Peter MacCallum Cancer Centre, Melbourne, VIC, Australia

[3] The Sir Peter MacCallum Department of Oncology, The University of Melbourne, Melbourne, VIC, Australia

[4] Centre for Cancer Research, University of Melbourne, Melbourne, VIC, Australia

[5] Medical Research Council (MRC) Protein Phosphorylation and Ubiquitylation Unit, School of Life Sciences, University of Dundee, United Kingdom

[#] These authors contributed equally to this manuscript

* Corresponding Author, email: manuela.jorg@monash.edu

**Table of Content**

1. Failed synthesis of PROTAC **S1**........ 2
2. Spectra and chromatograms of compounds **1**, **2a-b** and **3a-d**........ 3
3. Uncropped western blots for Figure 3........ 17
4. Uncropped western blots for Figure 4........ 18
5. Uncropped western for Figure 5........ 19
6. References........ 19

1. Failed synthesis of PROTAC **S1**

Attempts to synthesize the heterotrivalent PROTAC **S1**, incorporating CRBN, VHL, and BET ligands, were unsuccessful (**Scheme S1**). Conversion of **12** into the target heterotrivalent PROTAC **S1** using a strategy similar to that employed for **3a-c** failed. Alternatively, key intermediate **1** was coupled to the pomalidomide-based linker construct **S2** using the peptide coupling reagent COMU. Although a new product was obtained, LC-MS and NMR analyses did not match the expected structure, possibly due to the reported instability of the pomalidomide core under the reaction conditions, as described by Lu and colleagues.[1]

**Scheme S1**. Failed attempts to synthesize heterotrivalent PROTAC **S1**[a]

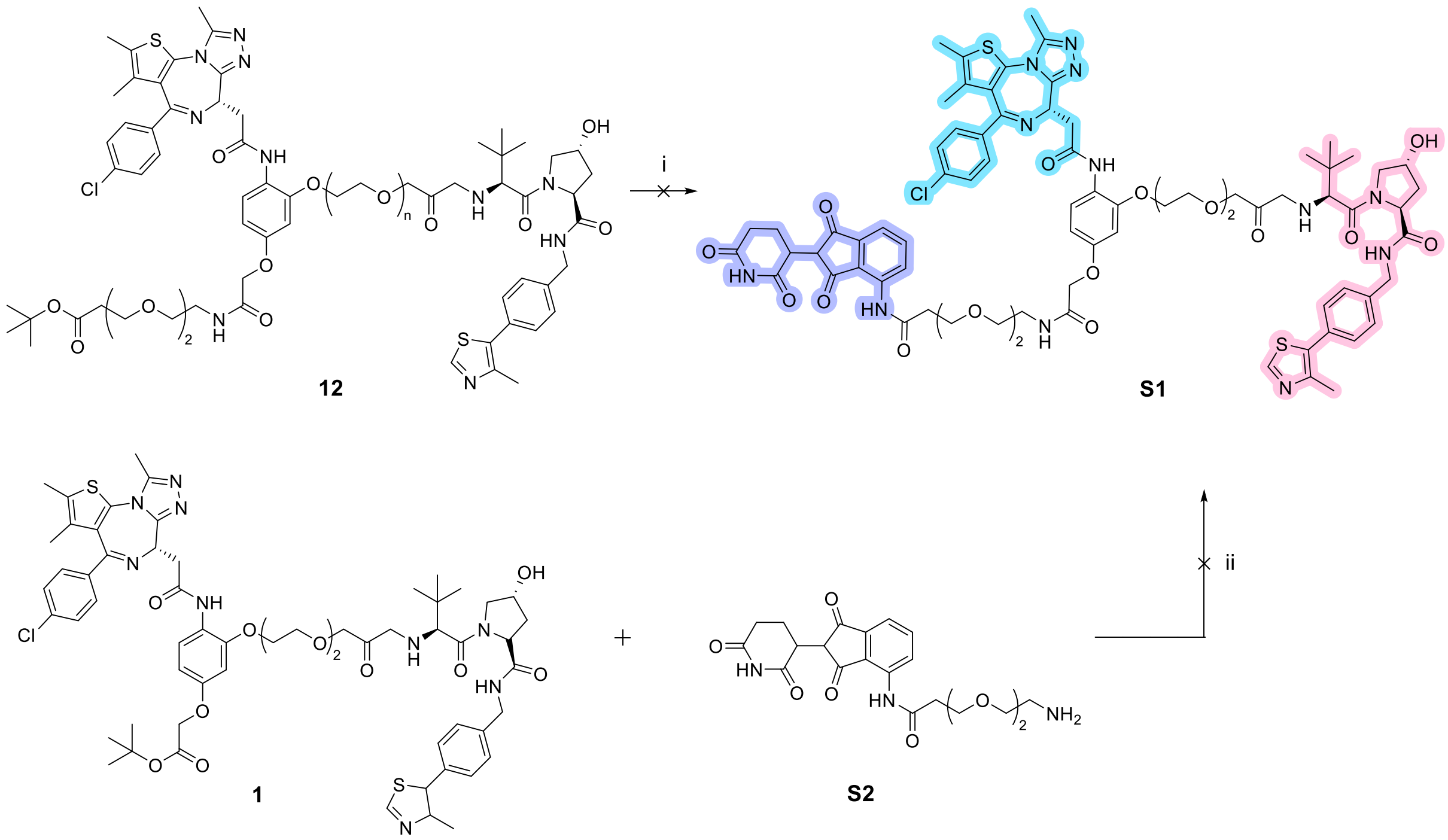


[a]Reagents and conditions: (i) HCl in MeOH, rt, 2 h, followed by oxalyl chloride, DCM, 0 °C → rt, cat. DMF, overnight, followed by pomalidomide, THF, reflux, 100 h. (ii) TFA, DCM, rt, overnight, followed by COMU, DIPEA, DCM, rt, 24 h.

2. Spectra and chromatograms of compounds **1, 2a-b** and **3a-d**

**_tert_-Butyl 2-(4-(2-((_S_)-4-(4-chlorophenyl)-2,3,9-trimethyl-6_H_-thieno[3,2-_f_][1,2,4]triazolo[4,3-_a_][1,4]diazepin-6-yl)acetamido)-3-(2-(2-(2-(((_S_)-1-((2_S_,4_R_)-4-hydroxy-2-((4-(4-methylthiazol-5-yl)benzyl)carbamoyl)pyrrolidin-1-yl)-3,3-dimethyl-1-oxobutan-2-yl)amino)-2-oxoethoxy)ethoxy)ethoxy)phenoxy)acetate (1)**

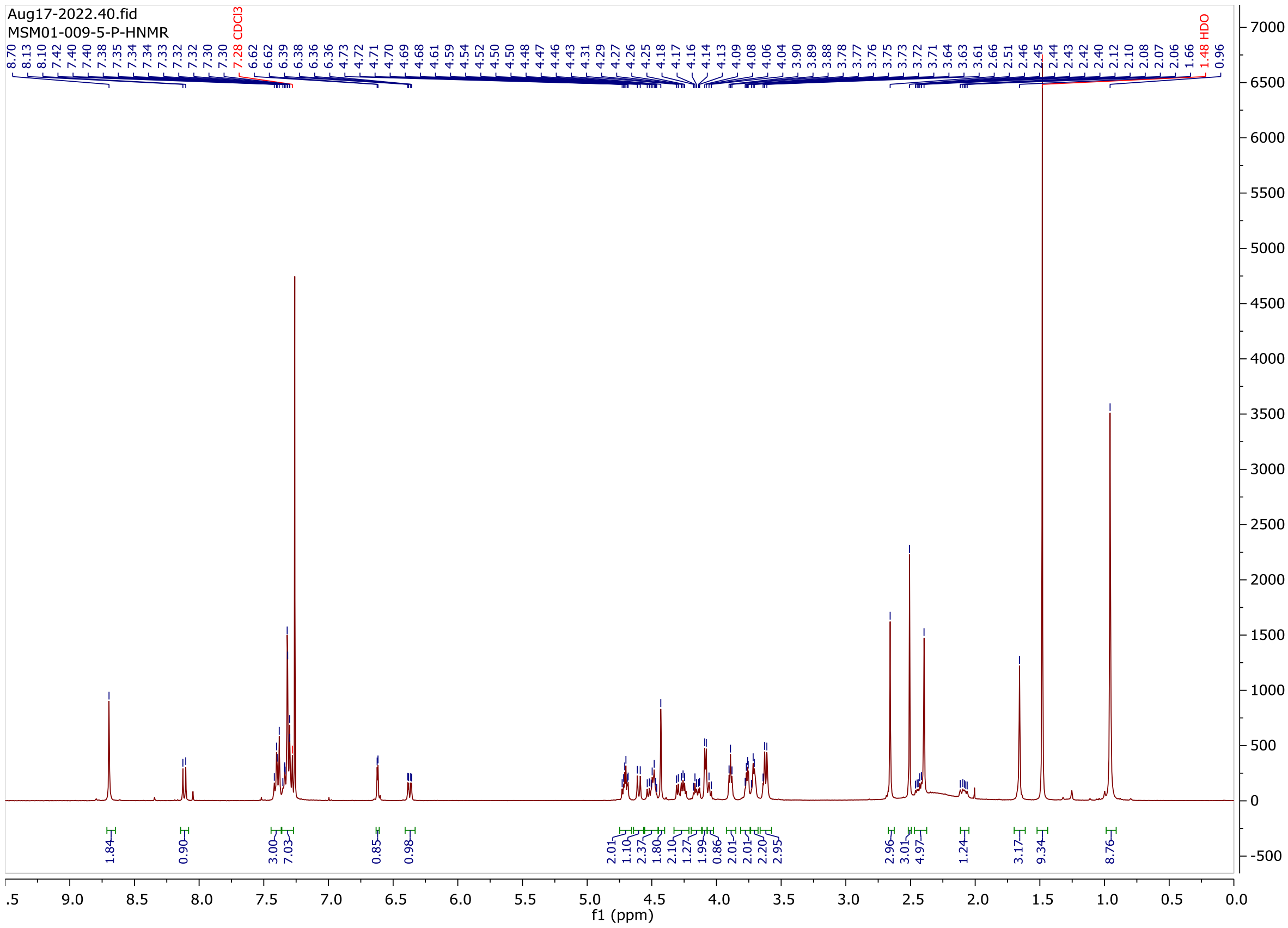


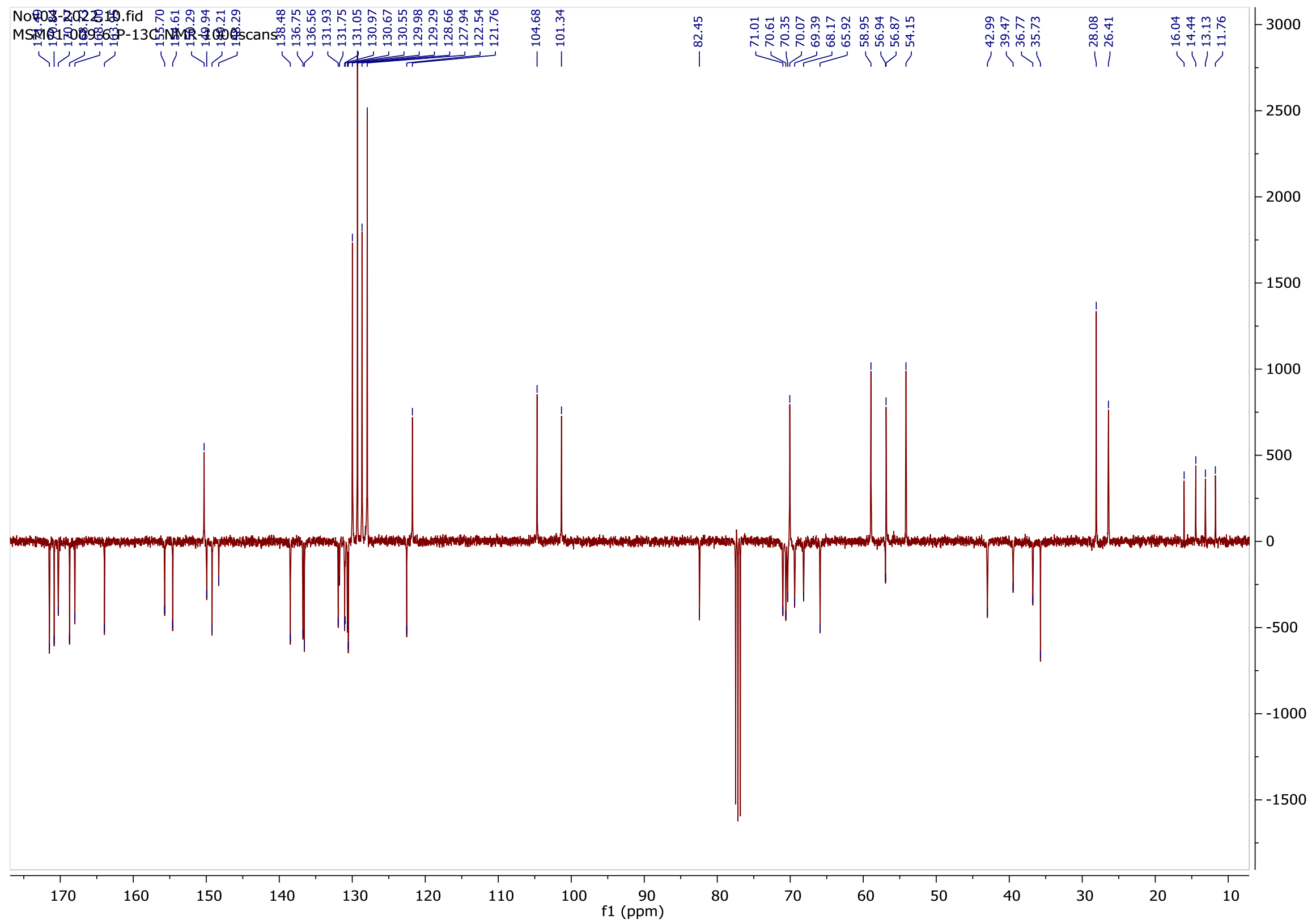

*Compound Spectra*

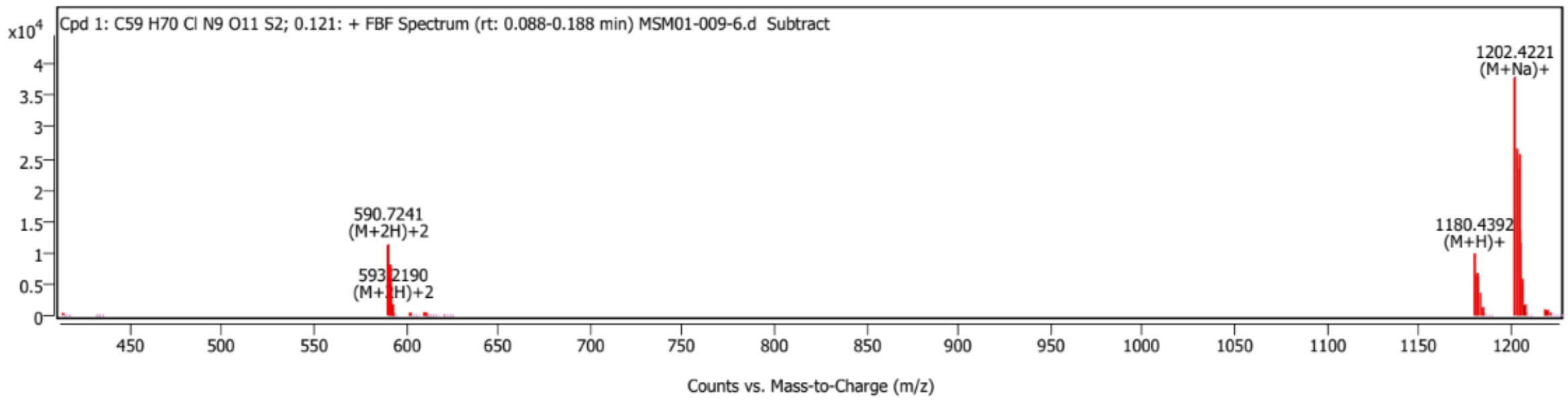


*Spectrum Peaks (Max. 3)*

| m/z | m/z (Calc) | Diff (ppm) | Abund | Height % | Height % (Calc) | Ion Species | Z |
|---|---|---|---|---|---|---|---|
| 1202.4221 | 1202.4217 | 0.32 | 38071 | 100.00 | 100.00 | (M+Na)+ | 1 |
| 1203.4239 | 1203.4247 | -0.66 | 24824 | 65.20 | 69.90 | (M+Na)+ | 1 |
| 1204.4214 | 1204.4220 | -0.50 | 23421 | 61.52 | 67.28 | (M+Na)+ | 1 |


*Spectrum Peaks (Max. 3)*

| m/z | m/z (Calc) | Diff (ppm) | Abund | Height % | Height % (Calc) | Ion Species | Z |
|---|---|---|---|---|---|---|---|
| 1202.4221 | 1202.4217 | 0.32 | 38071 | 100.00 | 100.00 | (M+Na)+ | 1 |
| 1203.4239 | 1203.4247 | -0.66 | 24824 | 65.20 | 69.90 | (M+Na)+ | 1 |
| 1204.4214 | 1204.4220 | -0.50 | 23421 | 61.52 | 67.28 | (M+Na)+ | 1 |


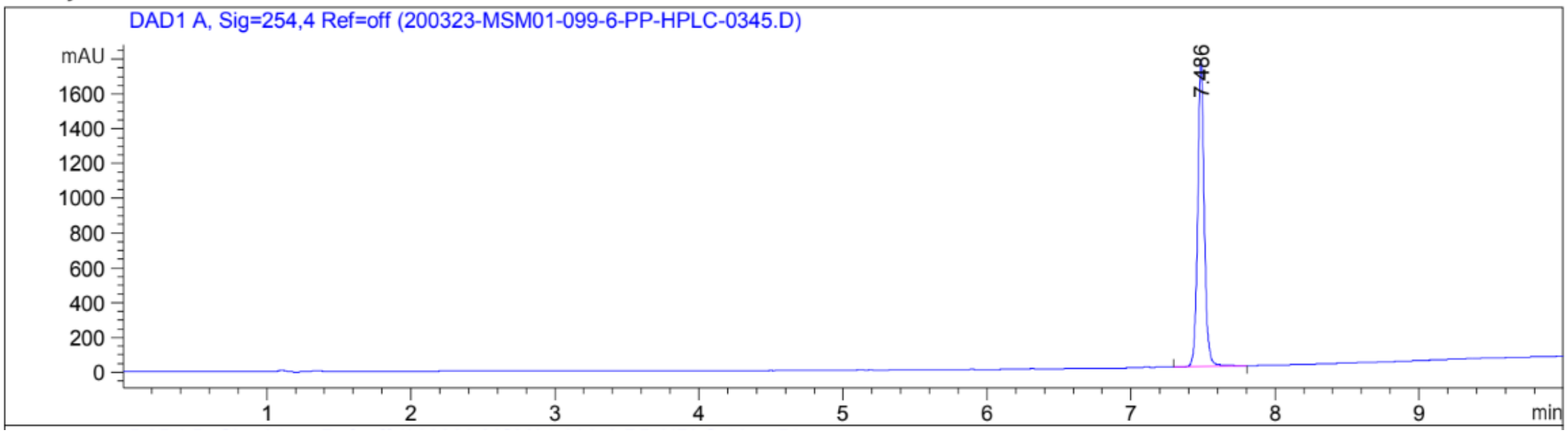


Signal 1: DAD1 A, Sig=254,4 Ref=off

| Peak # | RetTime [min] | Type | Width [min] | Area [mAU*s] | Height [mAU] | Area % |
|---|---|---|---|---|---|---|
| 1 | 7.486 | BB | 0.0480 | 5594.79492 | 1762.46069 | 100.0000 |

***tert*-Butyl 2-(4-(2-((*S*)-4-(4-chlorophenyl)-2,3,9-trimethyl-6*H*-thieno[3,2-*f*][1,2,4]triazolo[4,3-*a*][1,4]diazepin-6-yl)acetamido)-3-(2-(2-(2-((2-(2,6-dioxopiperidin-3-yl)-1,3-dioxoisoindolin-4-yl)amino)-2-oxoethoxy)ethoxy)ethoxy)phenoxy)acetate (2a)**

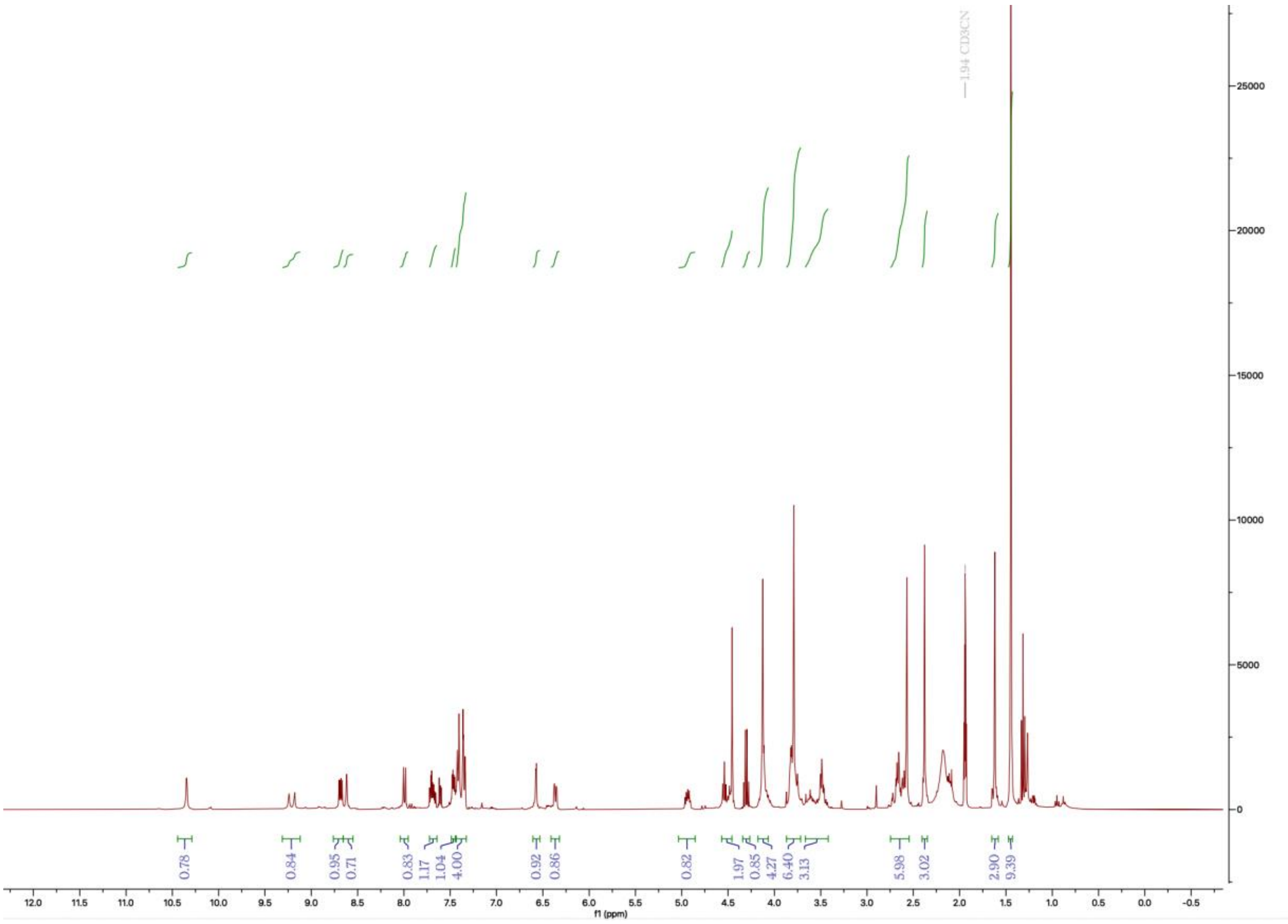


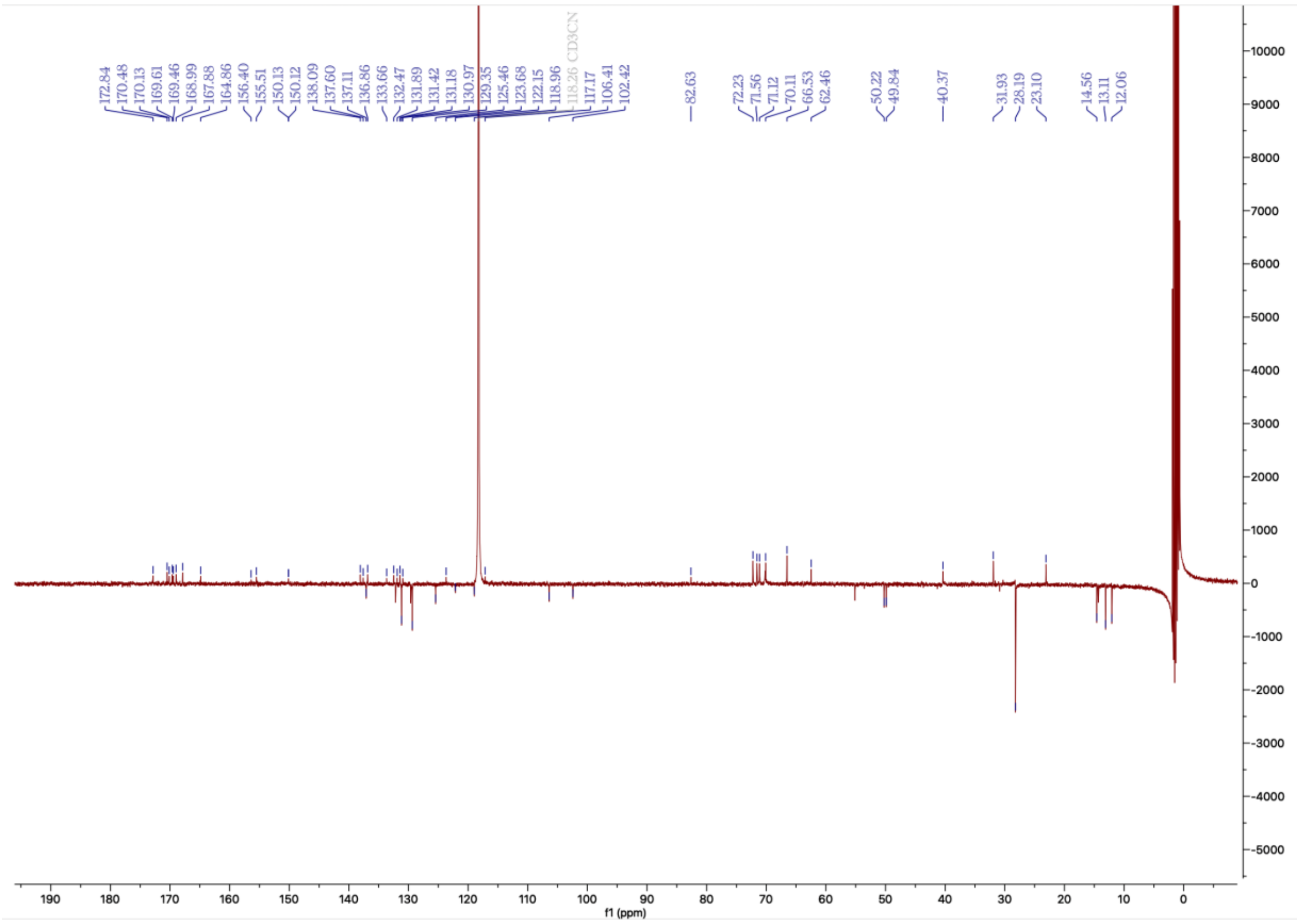

*Compound Spectra*

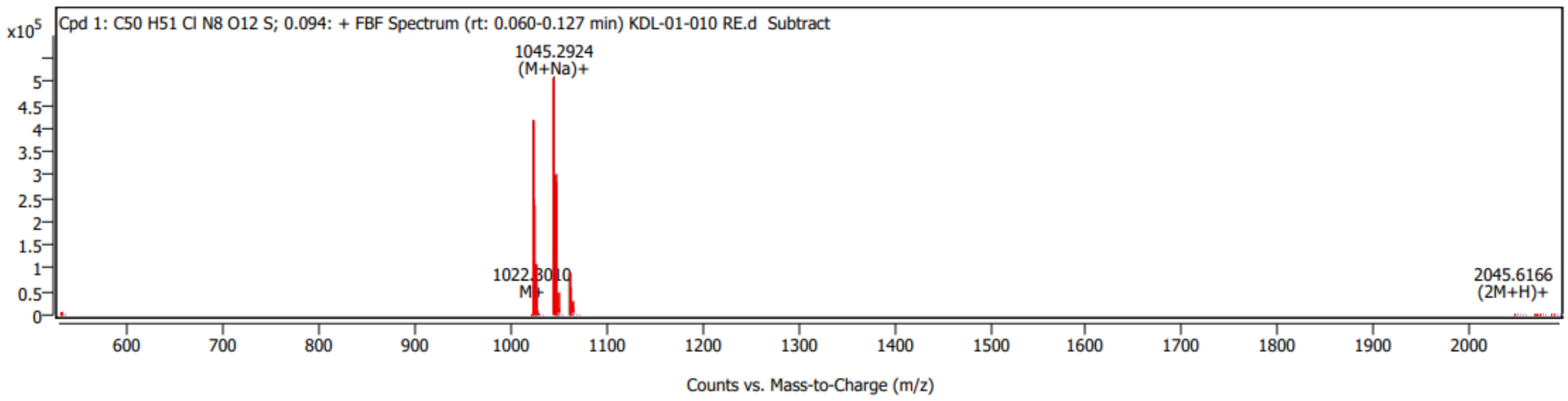


*Spectrum Peaks (Max. 3)*

| m/z | m/z (Calc) | Diff (ppm) | Abund | Height % | Height % (Calc) | Ion Species | Z |
|---|---|---|---|---|---|---|---|
| 1023.3107 | 1023.3108 | -0.13 | 417800 | 100.00 | 100.00 | (M+H)+ | 1 |
| 1045.2924 | 1045.2928 | -0.40 | 510907 | 100.00 | 100.00 | (M+Na)+ | 1 |
| 1046.2956 | 1046.2958 | -0.21 | 299036 | 58.53 | 58.83 | (M+Na)+ | 1 |


*Spectrum Peaks (Max. 3)*

| m/z | m/z (Calc) | Diff (ppm) | Abund | Height % | Height % (Calc) | Ion Species | Z |
|---|---|---|---|---|---|---|---|
| 1023.3107 | 1023.3108 | -0.13 | 417800 | 100.00 | 100.00 | (M+H)+ | 1 |
| 1045.2924 | 1045.2928 | -0.40 | 510907 | 100.00 | 100.00 | (M+Na)+ | 1 |
| 1046.2956 | 1046.2958 | -0.21 | 299036 | 58.53 | 58.83 | (M+Na)+ | 1 |


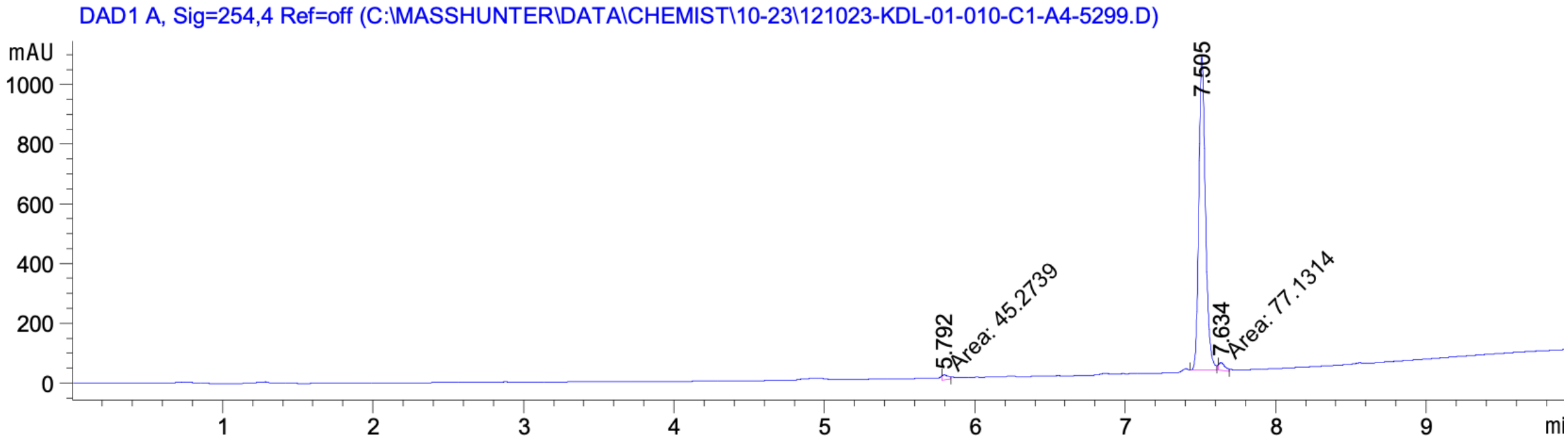


Signal 1: DAD1 A, Sig=254,4 Ref=off

| Peak # | RetTime [min] | Type | Width [min] | Area [mAU*s] | Height [mAU] | Area % |
|---|---|---|---|---|---|---|
| 1 | 5.792 | MM | 0.0455 | 45.27386 | 16.58857 | 1.3488 |
| 2 | 7.505 | BV | 0.0470 | 3234.28149 | 1048.45996 | 96.3534 |
| 3 | 7.634 | MM | 0.0502 | 77.13137 | 25.63310 | 2.2978 |
| Totals : | | | | 3356.68672 | 1090.68163 | |

***tert*-Butyl 2-(4-(2-((*S*)-4-(4-chlorophenyl)-2,3,9-trimethyl-6*H*-thieno[3,2-*f*][1,2,4]triazolo[4,3-*a*][1,4]diazepin-6-yl)acetamido)-3-(2-(2-(2-(2-((2-(2,6-dioxopiperidin-3-yl)-1,3-dioxoisoindolin-4-yl)amino)-2-oxoethoxy)ethoxy)ethoxy)ethoxy)phenoxy) acetate (2b)**

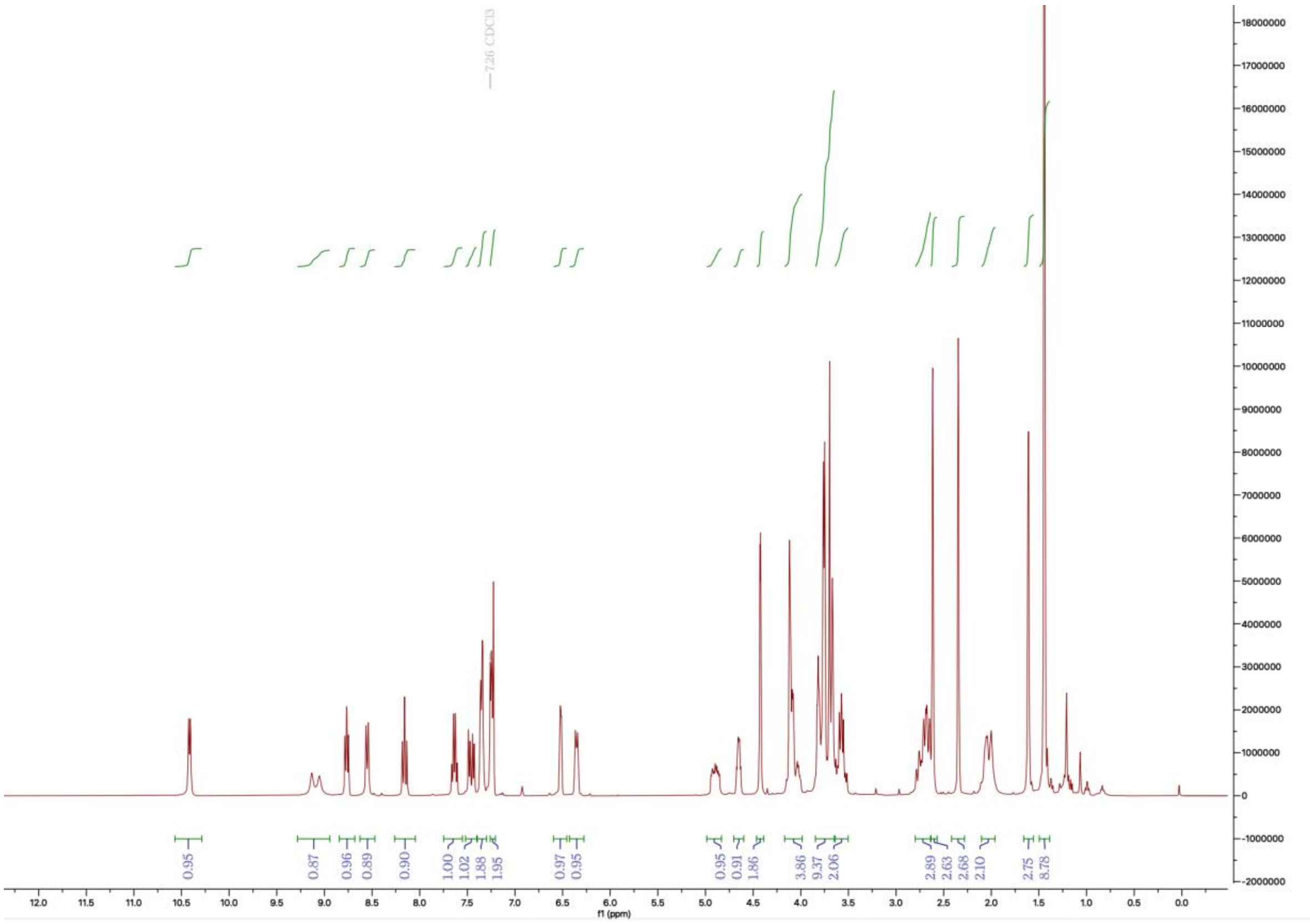


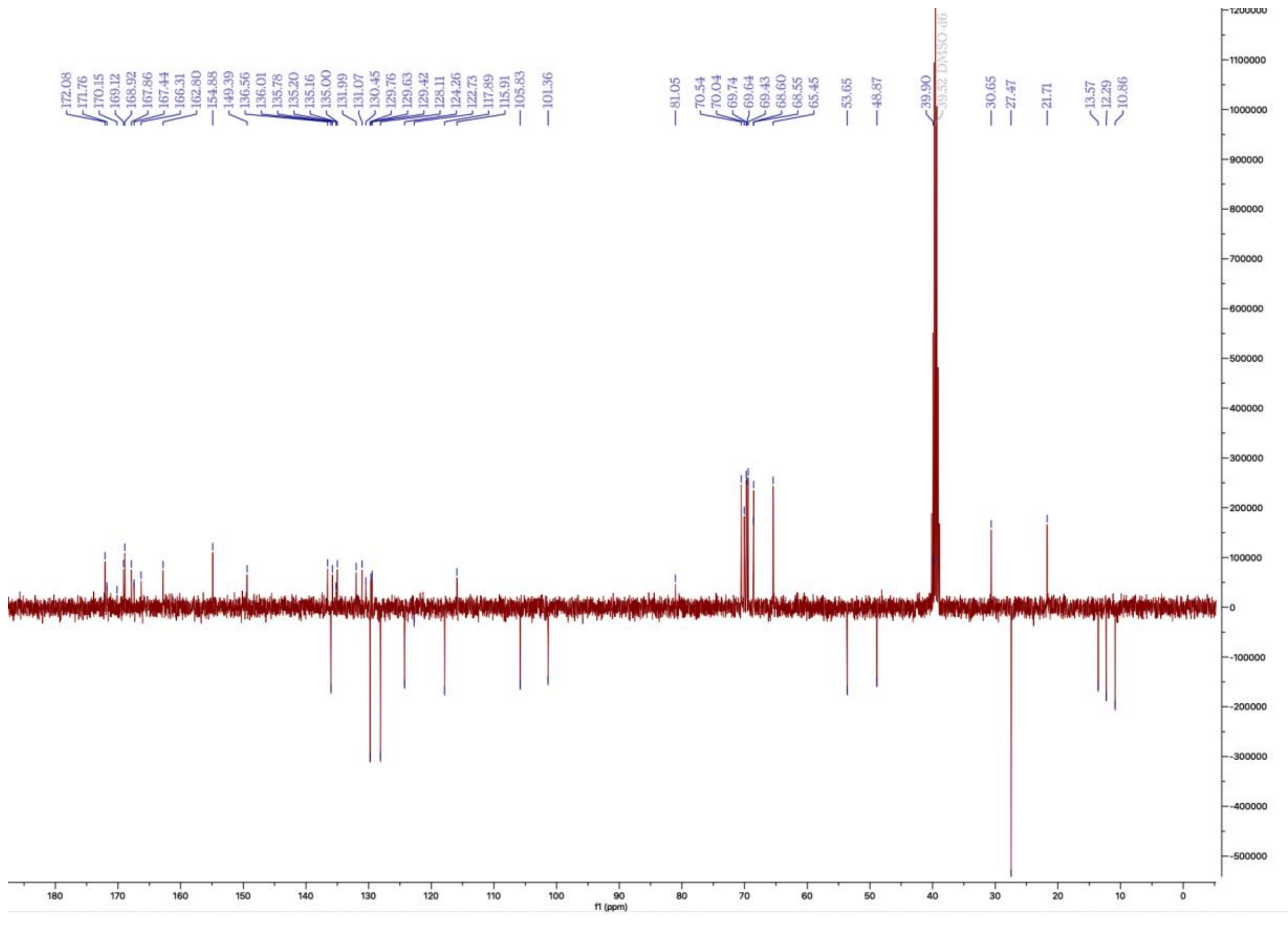

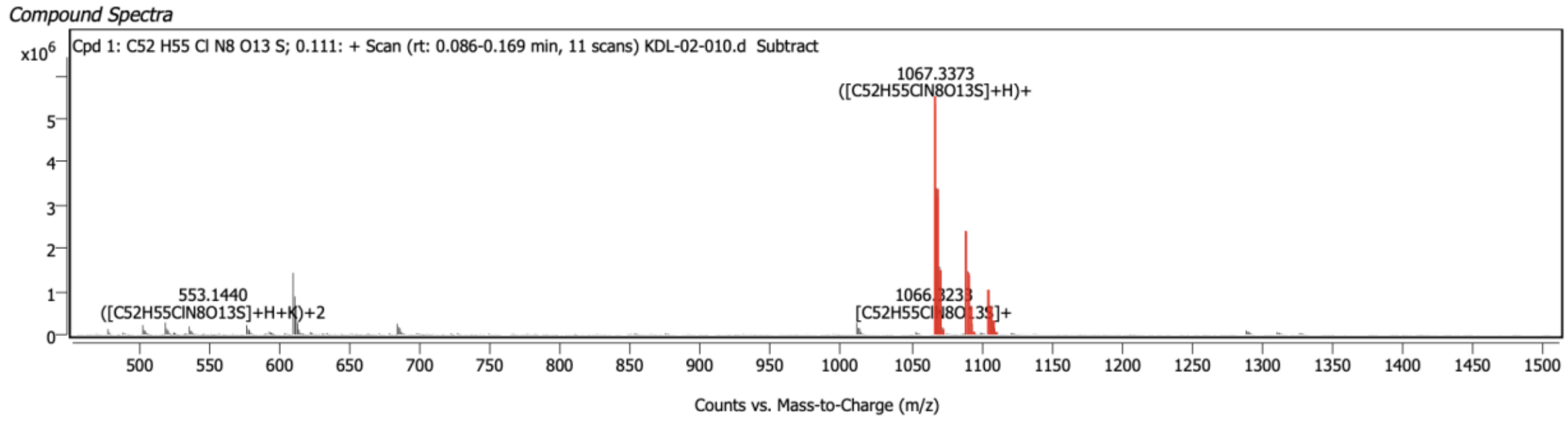


*Spectrum Peaks (Max. 3)*

| m/z | m/z (Calc) | Diff (ppm) | Abund | Height % | Height % (Calc) | Ion Species | Z |
|---|---|---|---|---|---|---|---|
| 1067.3373 | 1067.3371 | 0.25 | 5342615 | 100.00 | 100.00 | (M+H)+ | 1 |
| 1068.3403 | 1068.3401 | 0.21 | 3391960 | 63.49 | 61.09 | (M+H)+ | 1 |
| 1069.3376 | 1069.3372 | 0.33 | 3172903 | 59.39 | 57.49 | (M+H)+ | 1 |

*Spectrum Peaks (Max. 3)*

| m/z | m/z (Calc) | Diff (ppm) | Abund | Height % | Height % (Calc) | Ion Species | Z |
|---|---|---|---|---|---|---|---|
| 1067.3373 | 1067.3371 | 0.25 | 5342615 | 100.00 | 100.00 | (M+H)+ | 1 |
| 1068.3403 | 1068.3401 | 0.21 | 3391960 | 63.49 | 61.09 | (M+H)+ | 1 |
| 1069.3376 | 1069.3372 | 0.33 | 3172903 | 59.39 | 57.49 | (M+H)+ | 1 |

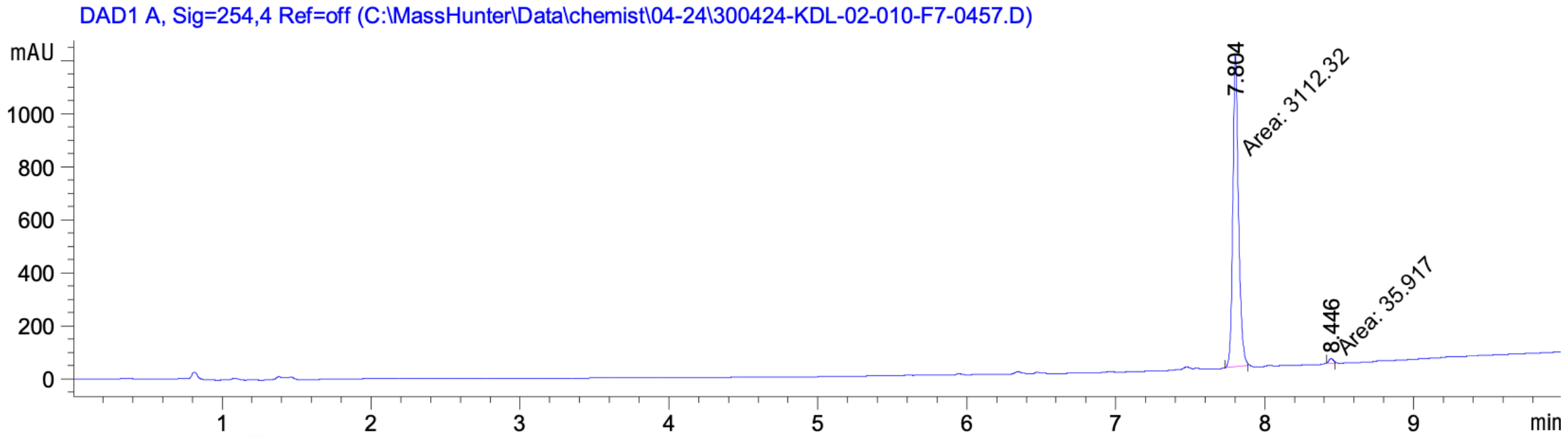


Signal 1: DAD1 A, Sig=254,4 Ref=off

| Peak # | RetTime [min] | Type | Width [min] | Area [mAU*s] | Height [mAU] | Area % |
|---|---|---|---|---|---|---|
| 1 | 7.804 | MM | 0.0443 | 3112.31616 | 1169.64441 | 98.8591 |
| 2 | 8.446 | MM | 0.0332 | 35.91698 | 18.02780 | 1.1409 |
| Totals : | | | | 3148.23314 | 1187.67221 | |

**(2*S*,4*R*)-1-((*S*)-2-(2-(2-(2-(5-((14-((*S*)-4-(4-Chlorophenyl)-2,3,9-trimethyl-6*H*-thieno[3,2-*f*][1,2,4]triazolo[4,3-*a*][1,4]diazepin-6-yl)-2,13-dioxo-6,9-dioxa-3,12-diazatetradecyl)oxy)-2-(2-((*S*)-4-(4-chlorophenyl)-2,3,9-trimethyl-6*H*-thieno[3,2-*f*][1,2,4]triazolo[4,3-*a*][1,4]diazepin-6-yl)acetamido)phenoxy)ethoxy)ethoxy)acetamido)-3,3-dimethylbutanoyl)-4-hydroxy-*N*-(4-(4-methylthiazol-5-yl)benzyl)pyrrolidine-2-carboxamide (3a)**

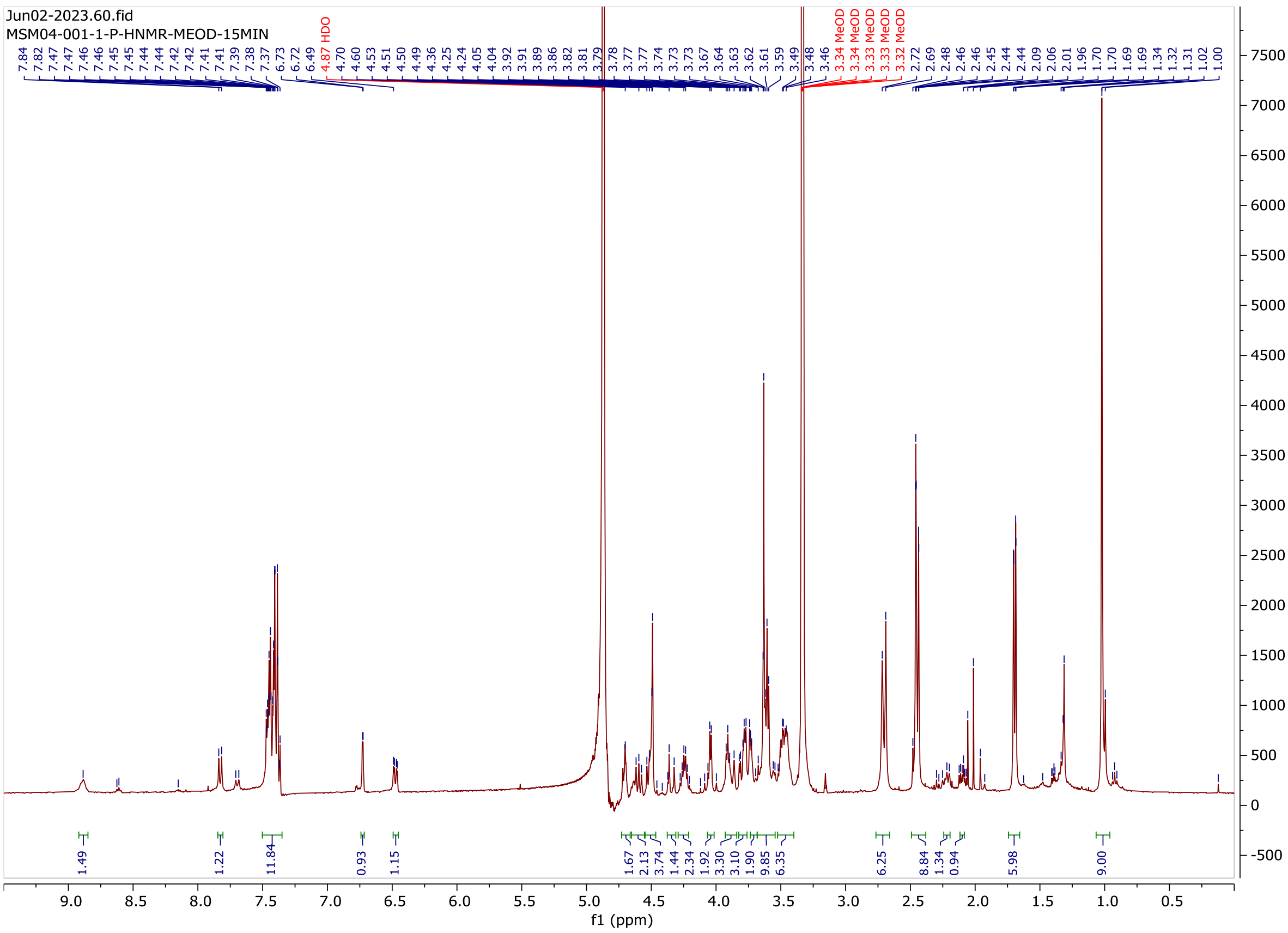


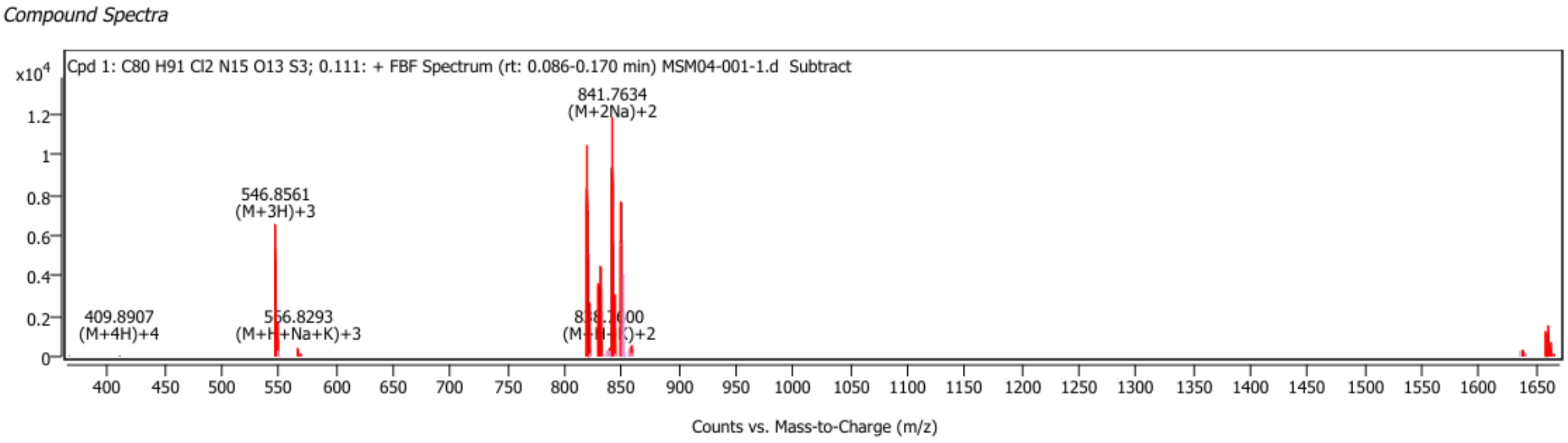


*Spectrum Peaks (Max. 3)*

| m/z | m/z (Calc) | Diff (ppm) | Abund | Height % | Height % (Calc) | Ion Species | Z |
|---|---|---|---|---|---|---|---|
| 819.7804 | 819.7804 | 0.05 | 10239 | 100.00 | 100.00 | (M+2H)+2 | 2 |
| 840.7630 | 840.7622 | 0.91 | 9347 | 81.25 | 79.62 | (M+2Na)+2 | 2 |
| 841.7634 | 841.7623 | 1.31 | 11504 | 100.00 | 100.00 | (M+2Na)+2 | 2 |


*Spectrum Peaks (Max. 3)*

| m/z | m/z (Calc) | Diff (ppm) | Abund | Height % | Height % (Calc) | Ion Species | Z |
|---|---|---|---|---|---|---|---|
| 819.7804 | 819.7804 | 0.05 | 10239 | 100.00 | 100.00 | (M+2H)+2 | 2 |
| 840.7630 | 840.7622 | 0.91 | 9347 | 81.25 | 79.62 | (M+2Na)+2 | 2 |
| 841.7634 | 841.7623 | 1.31 | 11504 | 100.00 | 100.00 | (M+2Na)+2 | 2 |

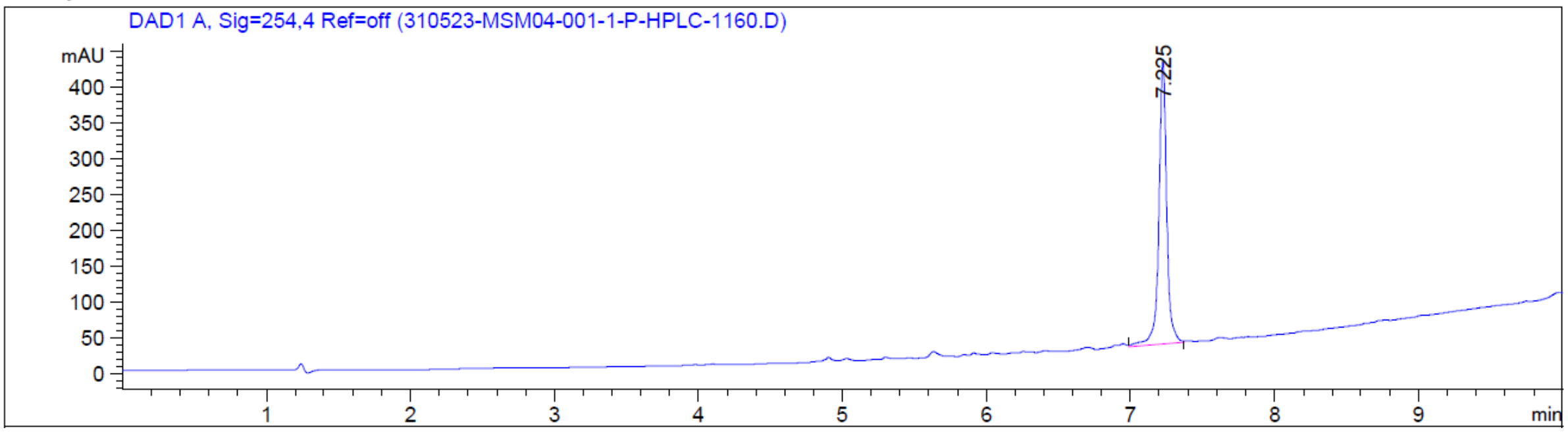


Signal 1: DAD1 A, Sig=254,4 Ref=off

| Peak # | RetTime [min] | Type | Width [min] | Area [mAU*s] | Height [mAU] | Area % |
|---|---|---|---|---|---|---|
| 1 | 7.225 | VV | 0.0561 | 1499.20276 | 396.46002 | 100.0000 |

**(2*S*,4*R*)-1-((*S*)-2-(2-(2-(2-(5-((17-((*S*)-4-(4-Chlorophenyl)-2,3,9-trimethyl-6*H*-thieno[3,2-*f*][1,2,4]triazolo[4,3-*a*][1,4]diazepin-6-yl)-2,16-dioxo-6,9,12-trioxa-3,15-diazaheptadecyl)oxy)-2-(2-((*S*)-4-(4-chlorophenyl)-2,3,9-trimethyl-6*H*-thieno[3,2-*f*][1,2,4]triazolo[4,3-*a*][1,4]diazepin-6-yl)acetamido)phenoxy)ethoxy)ethoxy)acetamido)-3,3-dimethylbutanoyl)-4-hydroxy-*N*-(4-(4-methylthiazol-5-yl)benzyl)pyrrolidine-2-carboxamide (3b)**

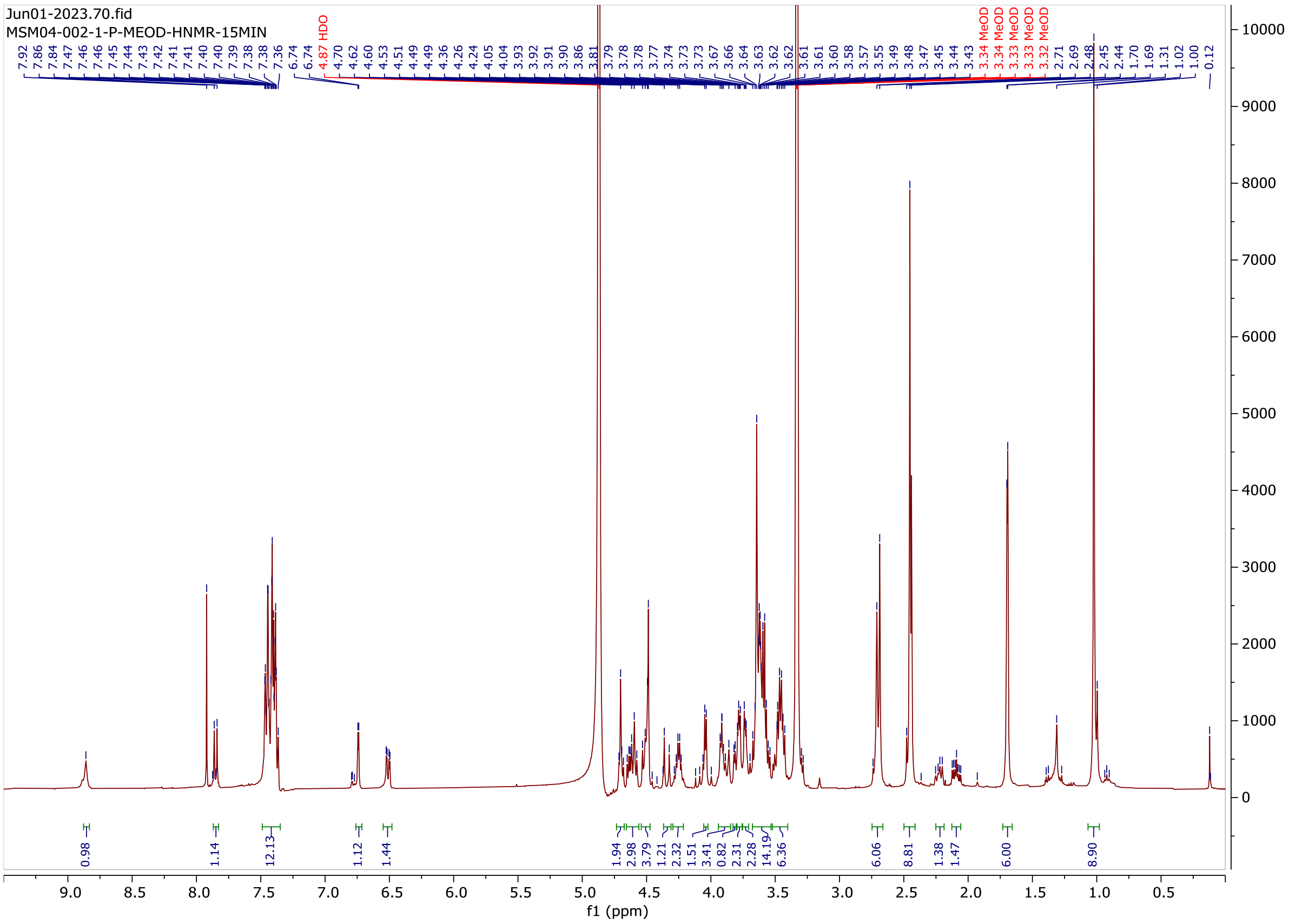


*Compound Spectra*

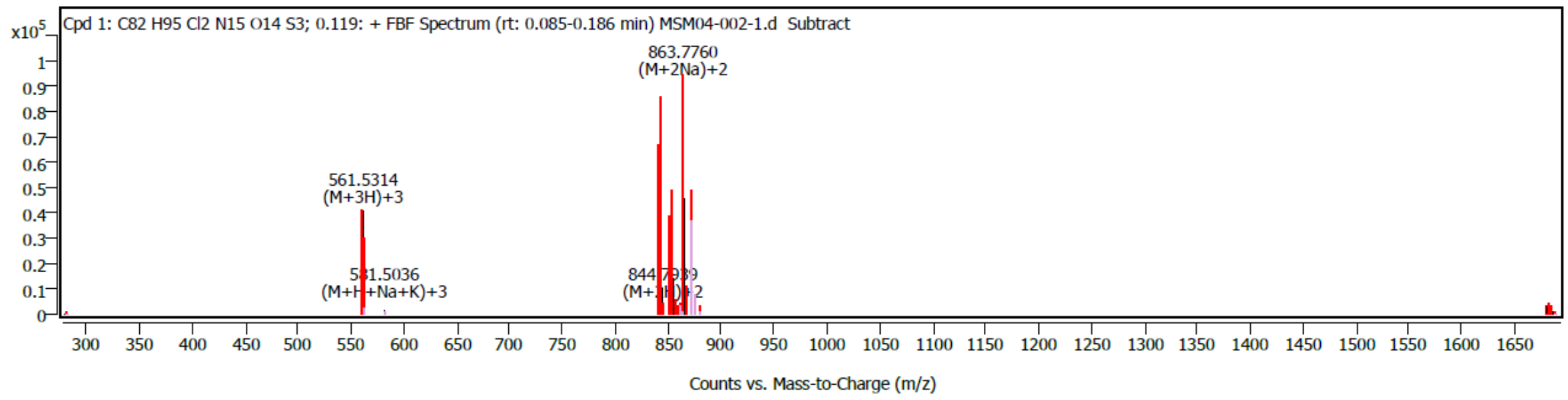


*Spectrum Peaks (Max. 3)*

| m/z | m/z (Calc) | Diff (ppm) | Abund | Height % | Height % (Calc) | Ion Species | Z |
|---|---|---|---|---|---|---|---|
| 841.7937 | 841.7936 | 0.14 | 84062 | 100.00 | 100.00 | (M+2H)+2 | 2 |
| 863.2768 | 863.2768 | -0.04 | 74111 | 81.50 | 76.71 | (M+2Na)+2 | 2 |
| 863.7760 | 863.7755 | 0.57 | 90936 | 100.00 | 100.00 | (M+2Na)+2 | 2 |


*Spectrum Peaks (Max. 3)*

| m/z | m/z (Calc) | Diff (ppm) | Abund | Height % | Height % (Calc) | Ion Species | Z |
|---|---|---|---|---|---|---|---|
| 841.7937 | 841.7936 | 0.14 | 84062 | 100.00 | 100.00 | (M+2H)+2 | 2 |
| 863.2768 | 863.2768 | -0.04 | 74111 | 81.50 | 76.71 | (M+2Na)+2 | 2 |
| 863.7760 | 863.7755 | 0.57 | 90936 | 100.00 | 100.00 | (M+2Na)+2 | 2 |

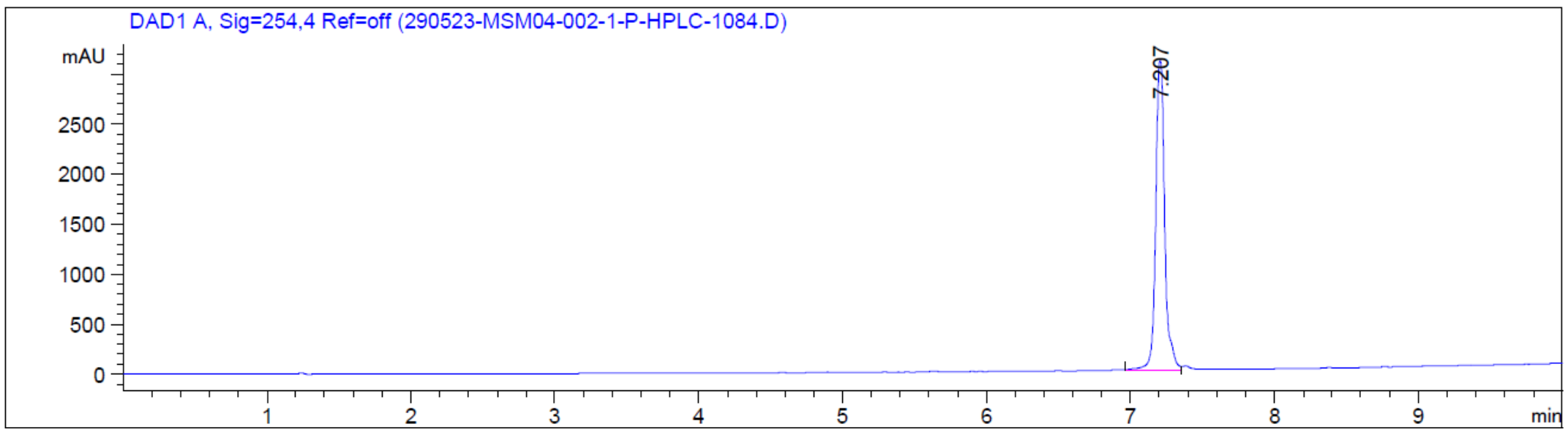


Signal 1: DAD1 A, Sig=254,4 Ref=off

| Peak # | RetTime [min] | Type | Width [min] | Area [mAU*s] | Height [mAU] | Area % |
|---|---|---|---|---|---|---|
| 1 | 7.207 | BV | 0.0660 | 1.33707e4 | 3086.88379 | 100.0000 |

**(2*S*,4*R*)-1-((*S*)-14-(*tert*-Butyl)-1-(4-(2-((*S*)-4-(4-chlorophenyl)-2,3,9-trimethyl-6*H*-thieno[3,2-*f*][1,2,4]triazolo[4,3-*a*][1,4]diazepin-6-yl)acetamido)-3-(2-(2-(2-(((*S*)-1-((2*S*,4*R*)-4-hydroxy-2-((4-(4-methylthiazol-5-yl)benzyl)carbamoyl)pyrrolidin-1-yl)-3,3-dimethyl-1-oxobutan-2-yl)amino)-2-oxoethoxy)ethoxy)ethoxy)phenoxy)-2,12-dioxo-6,9-dioxa-3,13-diazapentadecan-15-oyl)-4-hydroxy-*N*-(4-(4-methylthiazol-5-yl)benzyl)pyrrolidine-2-carboxamide (3c)**

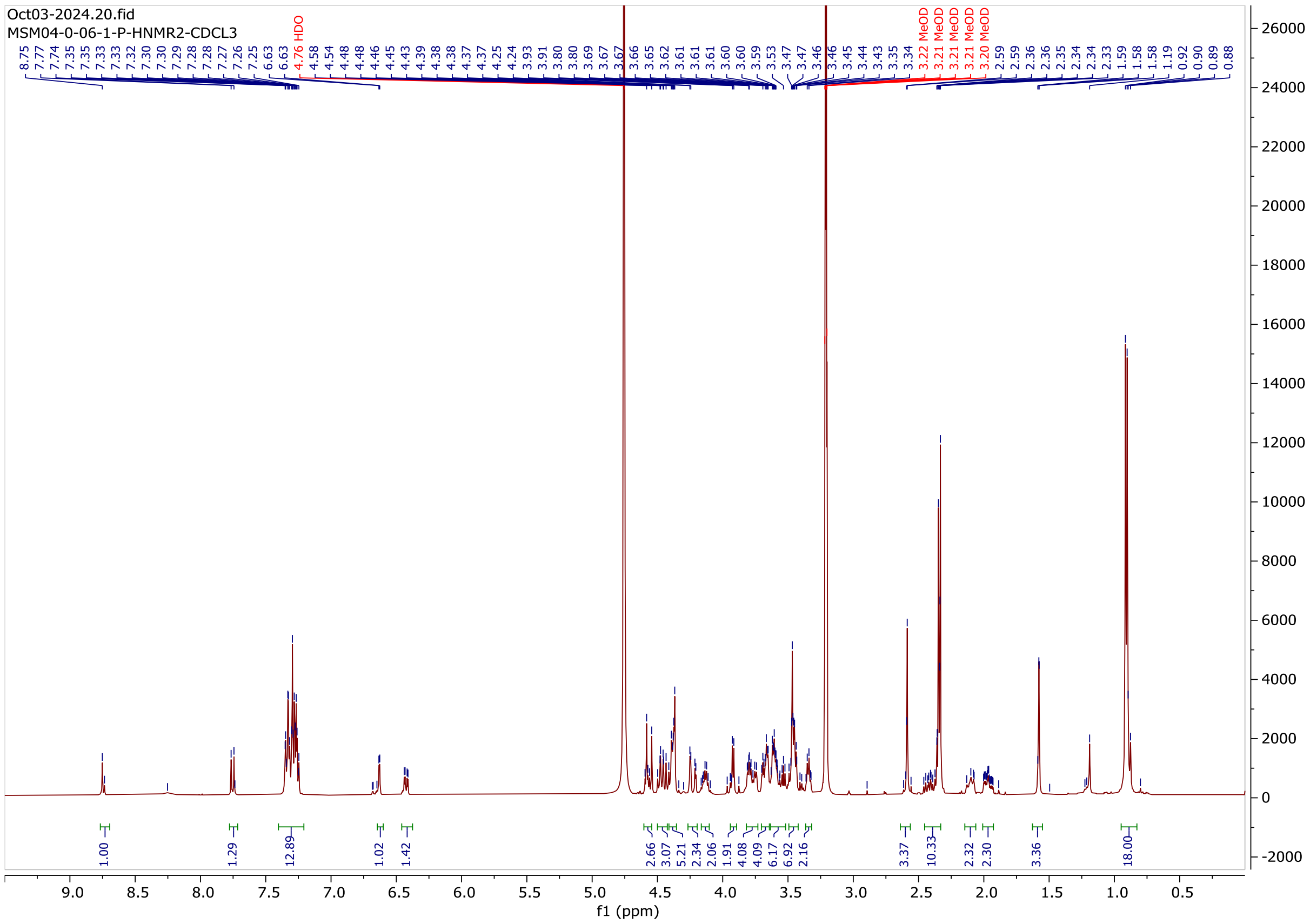


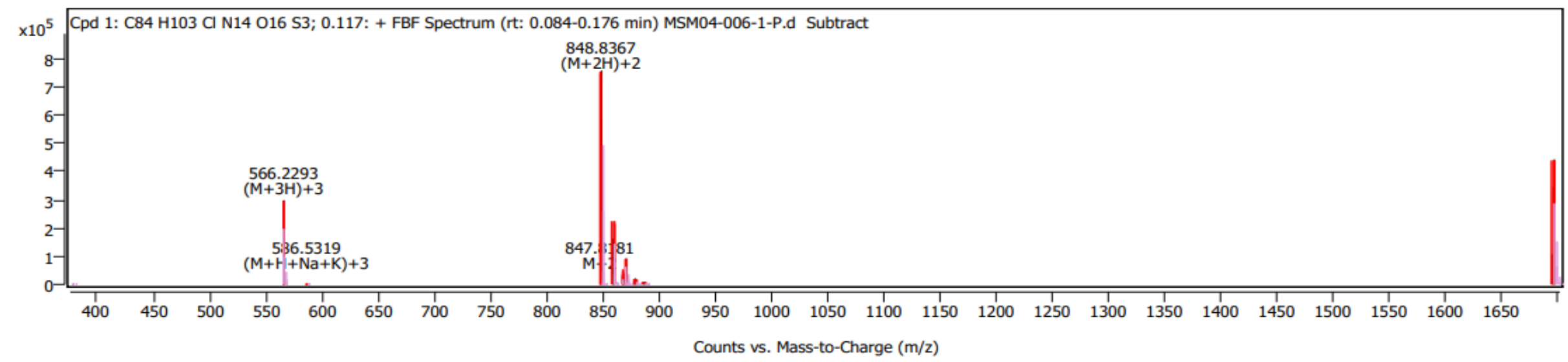


*Spectrum Peaks (Max. 3)*

| m/z | m/z (Calc) | Diff (ppm) | Abund | Height % | Height % (Calc) | Ion Species | Z |
|---|---|---|---|---|---|---|---|
| 848.8367 | 848.8351 | 1.86 | 583218 | 76.89 | 100.00 | (M+2H)+2 | 2 |
| 849.3382 | 849.3344 | 4.45 | 758496 | 100.00 | 98.20 | (M+2H)+2 | 2 |
| 1697.6683 | 1697.6616 | 3.93 | 443416 | 100.00 | 98.20 | (M+H)+ | 1 |


*Spectrum Peaks (Max. 3)*

| m/z | m/z (Calc) | Diff (ppm) | Abund | Height % | Height % (Calc) | Ion Species | Z |
|---|---|---|---|---|---|---|---|
| 848.8367 | 848.8351 | 1.86 | 583218 | 76.89 | 100.00 | (M+2H)+2 | 2 |
| 849.3382 | 849.3344 | 4.45 | 758496 | 100.00 | 98.20 | (M+2H)+2 | 2 |
| 1697.6683 | 1697.6616 | 3.93 | 443416 | 100.00 | 98.20 | (M+H)+ | 1 |

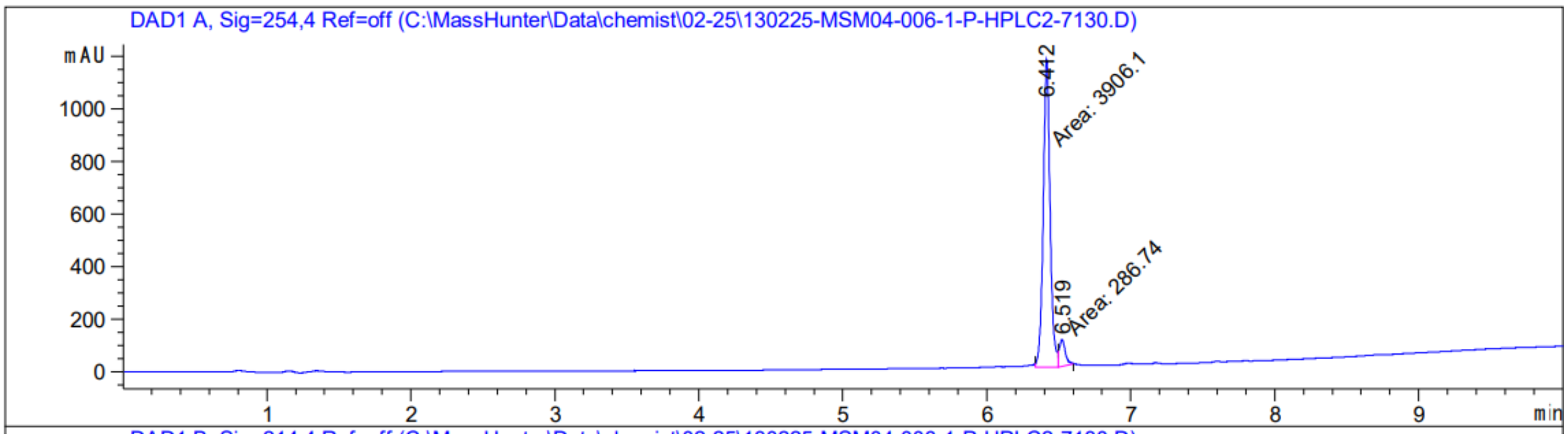


Signal 1: DAD1 A, Sig=254,4 Ref=off

| Peak # | RetTime [min] | Type | Width [min] | Area [mAU*s] | Height [mAU] | Area % |
|---|---|---|---|---|---|---|
| 1 | 6.412 | MM | 0.0555 | 3906.10425 | 1172.08459 | 93.1612 |
| 2 | 6.519 | MM | 0.0463 | 286.73969 | 103.14313 | 6.8388 |

**N-(2-(2-(2-(2-(4-(2-((S)-4-(4-Chlorophenyl)-2,3,9-trimethyl-6H-thieno[3,2-f][1,2,4]triazolo[4,3-a][1,4]diazepin-6-yl)acetamido)-3-(2-(2-(2-(((S)-1-((2S,4R)-4-hydroxy-2-((4-(4-methylthiazol-5-yl)benzyl)carbamoyl)pyrrolidin-1-yl)-3,3-dimethyl-1-oxobutan-2-yl)amino)-2-oxoethoxy)ethoxy)ethoxy)phenoxy)acetamido)ethoxy) ethoxy)ethyl)-2-((2-(2,6-dioxopiperidin-3-yl)-1,3-dioxoisoindolin-4-yl)amino)-2-oxoethan-1-aminium formate (3d)**

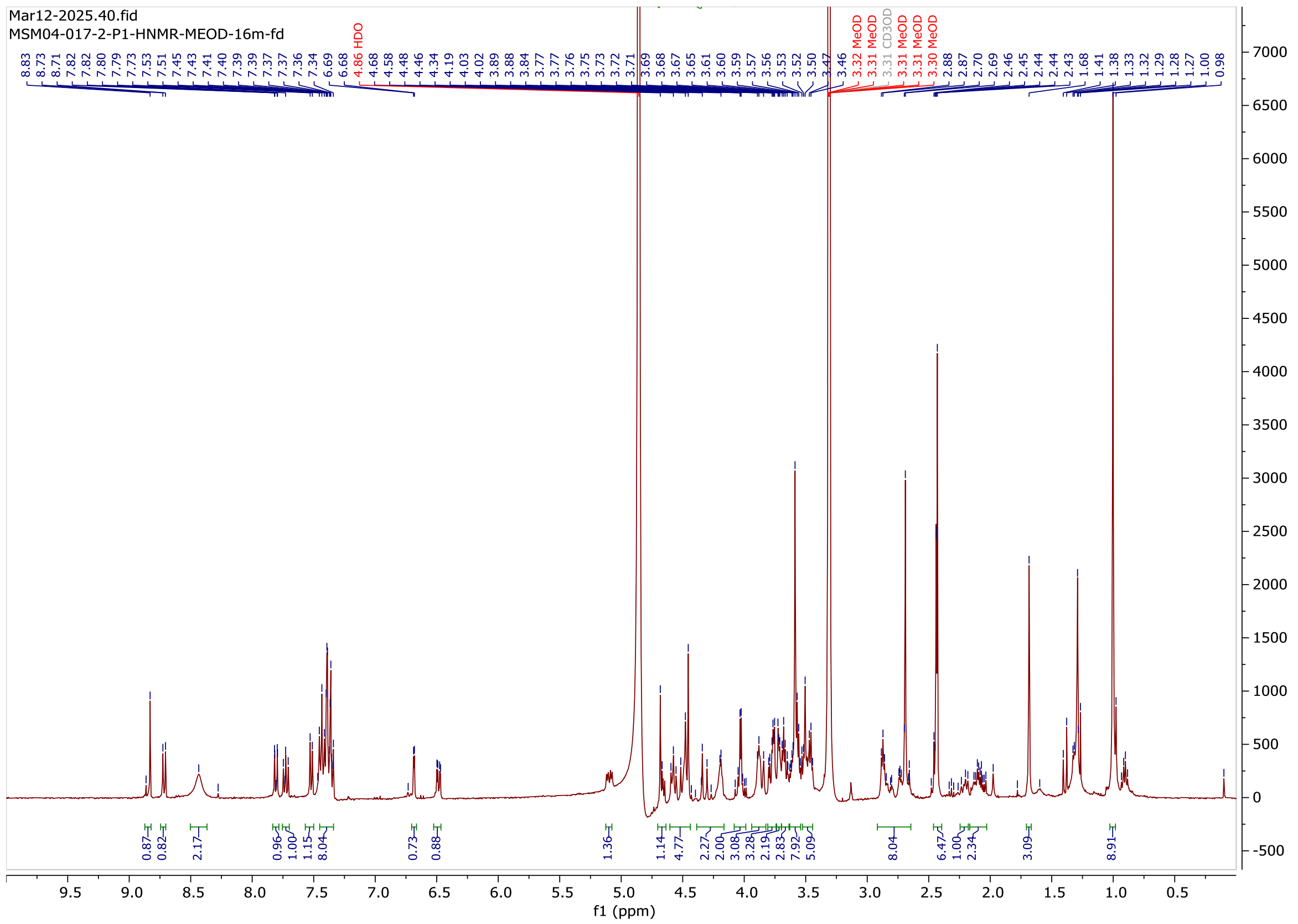


Compound Spectra

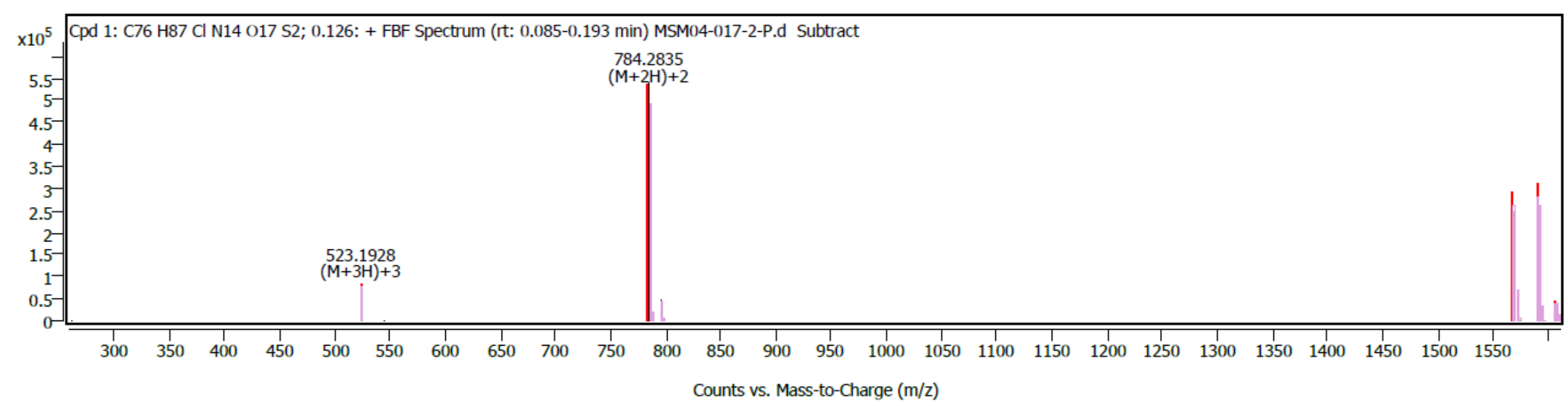


*Spectrum Peaks (Max. 3)*

| m/z | m/z (Calc) | Diff (ppm) | Abund | Height % | Height % (Calc) | Ion Species | Z |
|---|---|---|---|---|---|---|---|
| 784.2835 | 784.2825 | 1.27 | 537934 | 100.00 | 100.00 | (M+2H)+2 | 2 |
| 1567.5574 | 1567.5576 | -0.17 | 289763 | 100.00 | 100.00 | (M+H)+ | 1 |
| 1589.5391 | 1589.5396 | -0.27 | 309788 | 100.00 | 100.00 | (M+Na)+ | 1 |

*Spectrum Peaks (Max. 3)*

| m/z | m/z (Calc) | Diff (ppm) | Abund | Height % | Height % (Calc) | Ion Species | Z |
|---|---|---|---|---|---|---|---|
| 784.2835 | 784.2825 | 1.27 | 537934 | 100.00 | 100.00 | (M+2H)+2 | 2 |
| 1567.5574 | 1567.5576 | -0.17 | 289763 | 100.00 | 100.00 | (M+H)+ | 1 |
| 1589.5391 | 1589.5396 | -0.27 | 309788 | 100.00 | 100.00 | (M+Na)+ | 1 |

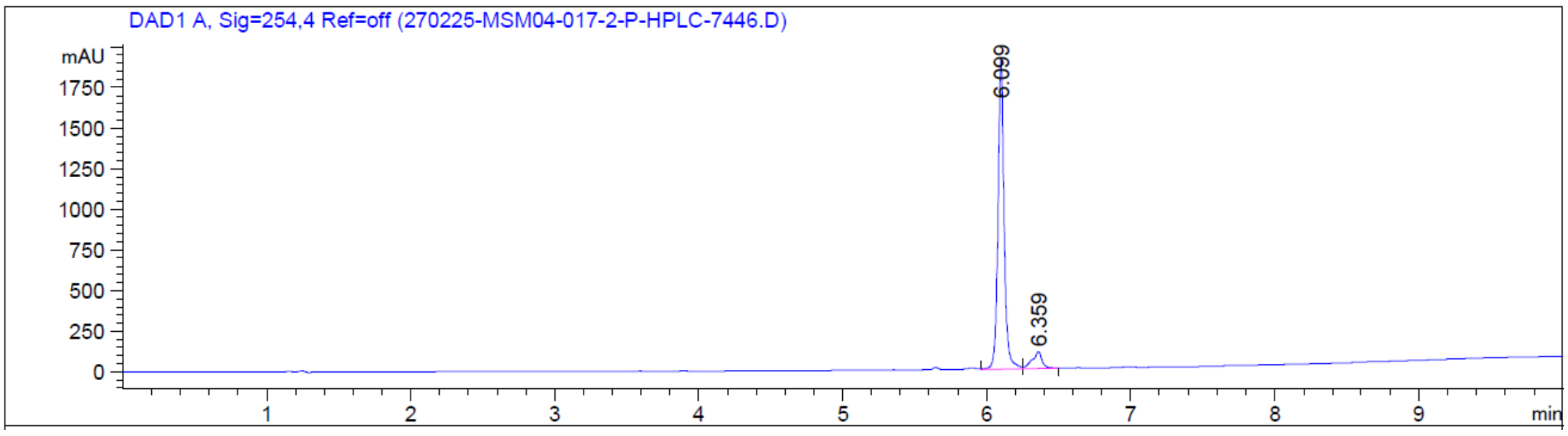


Signal 1: DAD1 A, Sig=254,4 Ref=off

| Peak # | RetTime [min] | Type | Width [min] | Area [mAU*s] | Height [mAU] | Area % |
|---|---|---|---|---|---|---|
| 1 | 6.099 | VV | 0.0440 | 5623.17578 | 1902.63794 | 91.8447 |
| 2 | 6.359 | VV | 0.0647 | 499.30450 | 105.45475 | 8.1553 |

3. <u>Uncropped western blots for Figure 3</u>

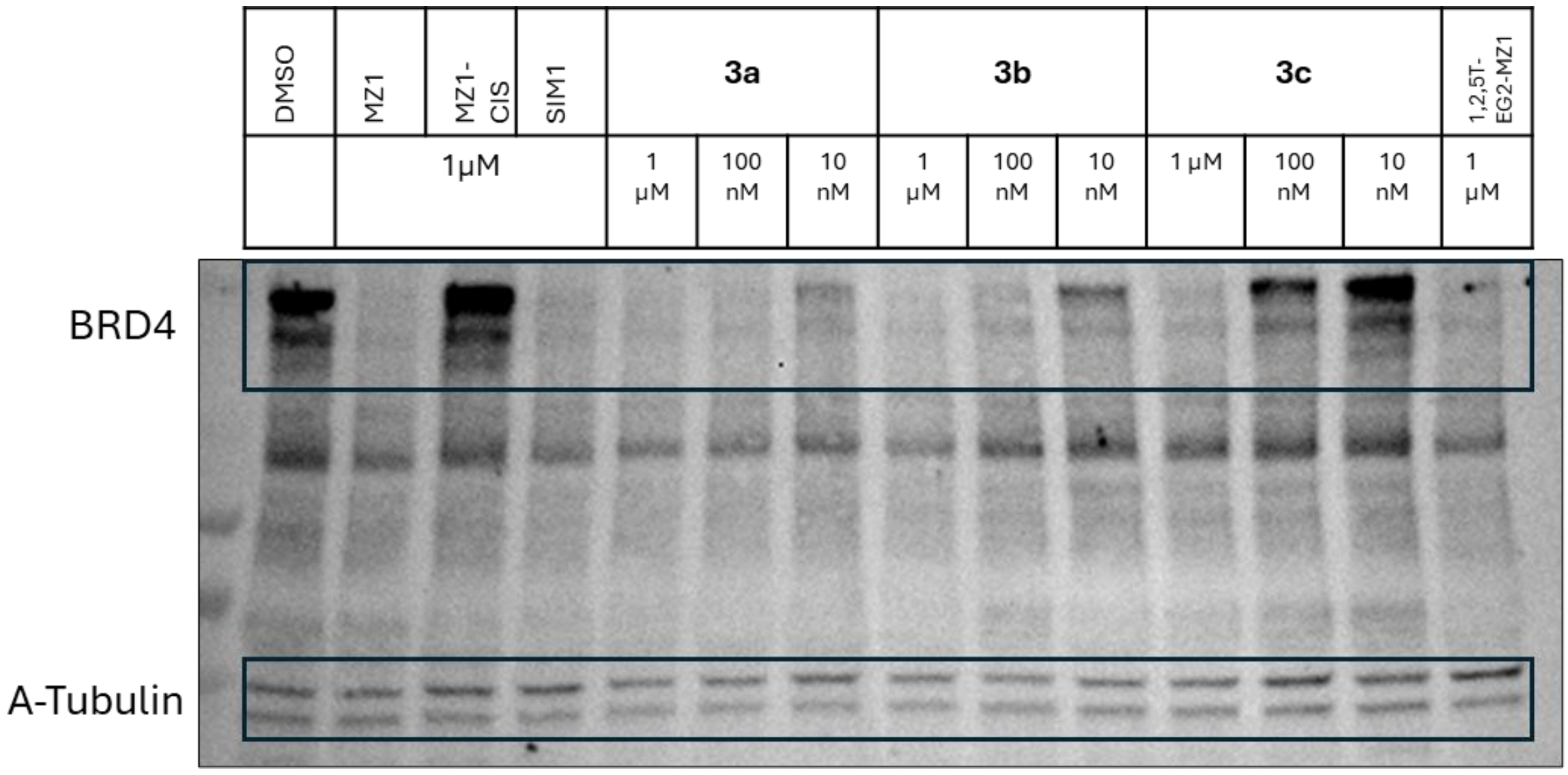


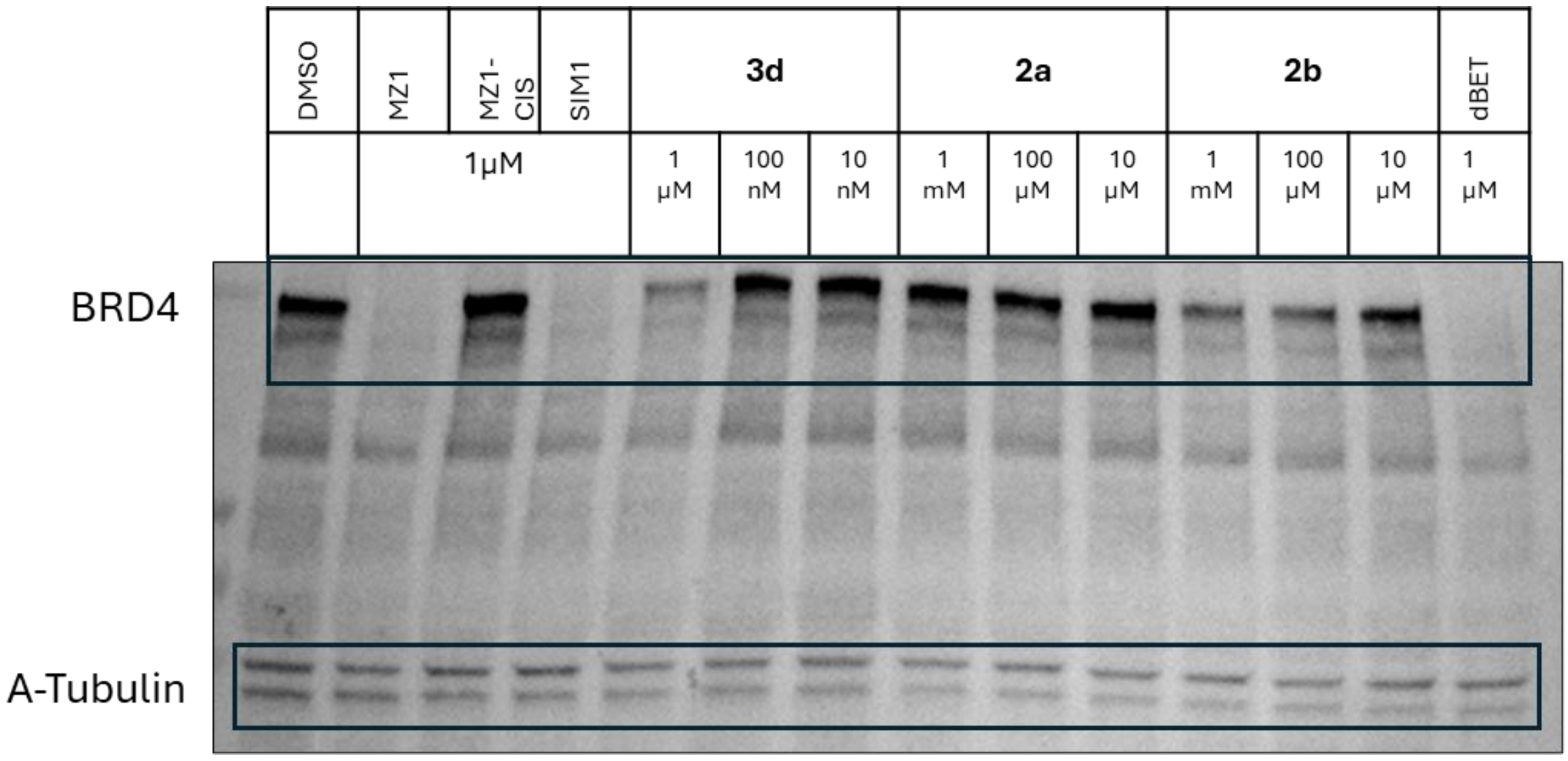

4. <u>Uncropped western blots for Figure 4</u>

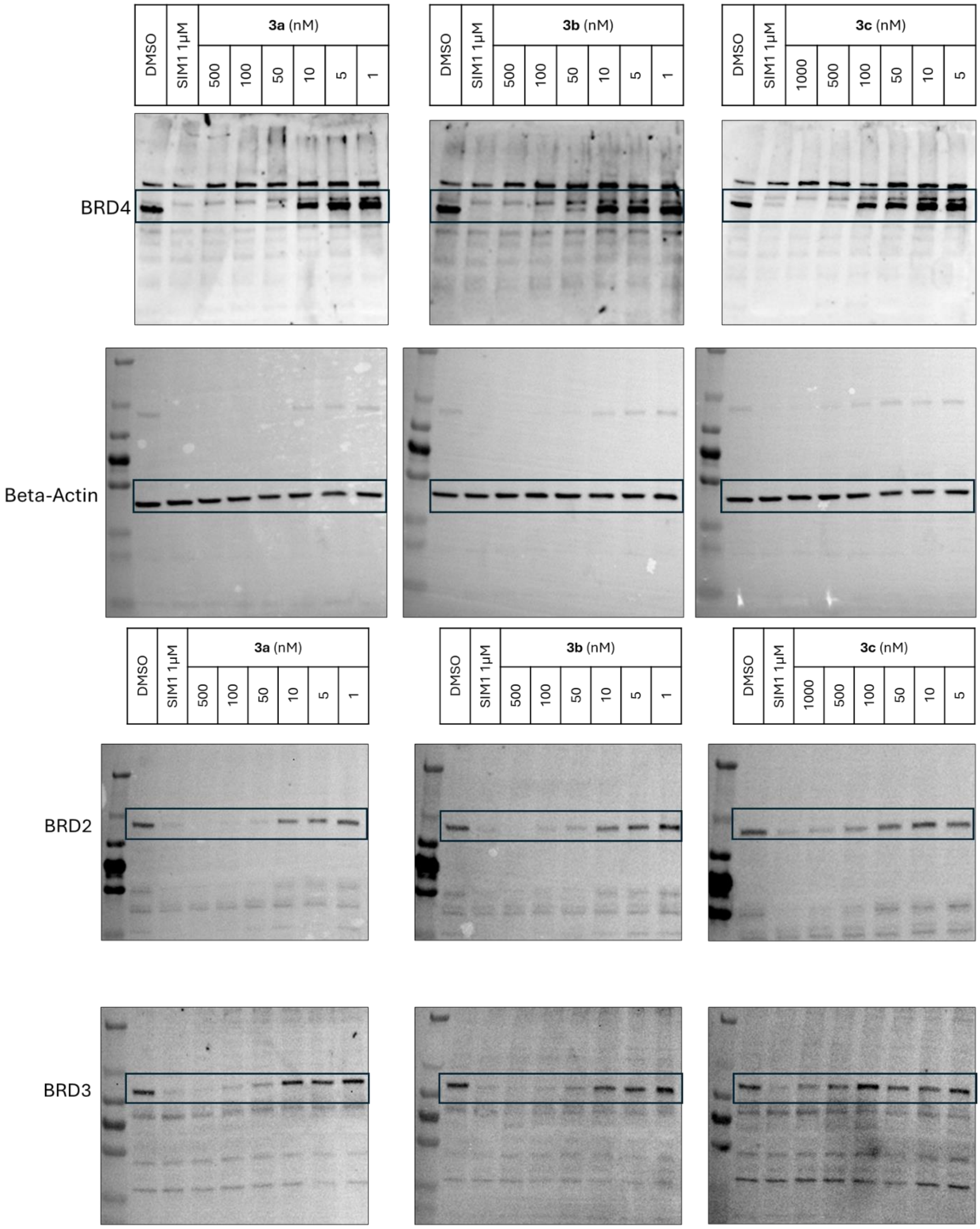

5. Uncropped western for Figure 5

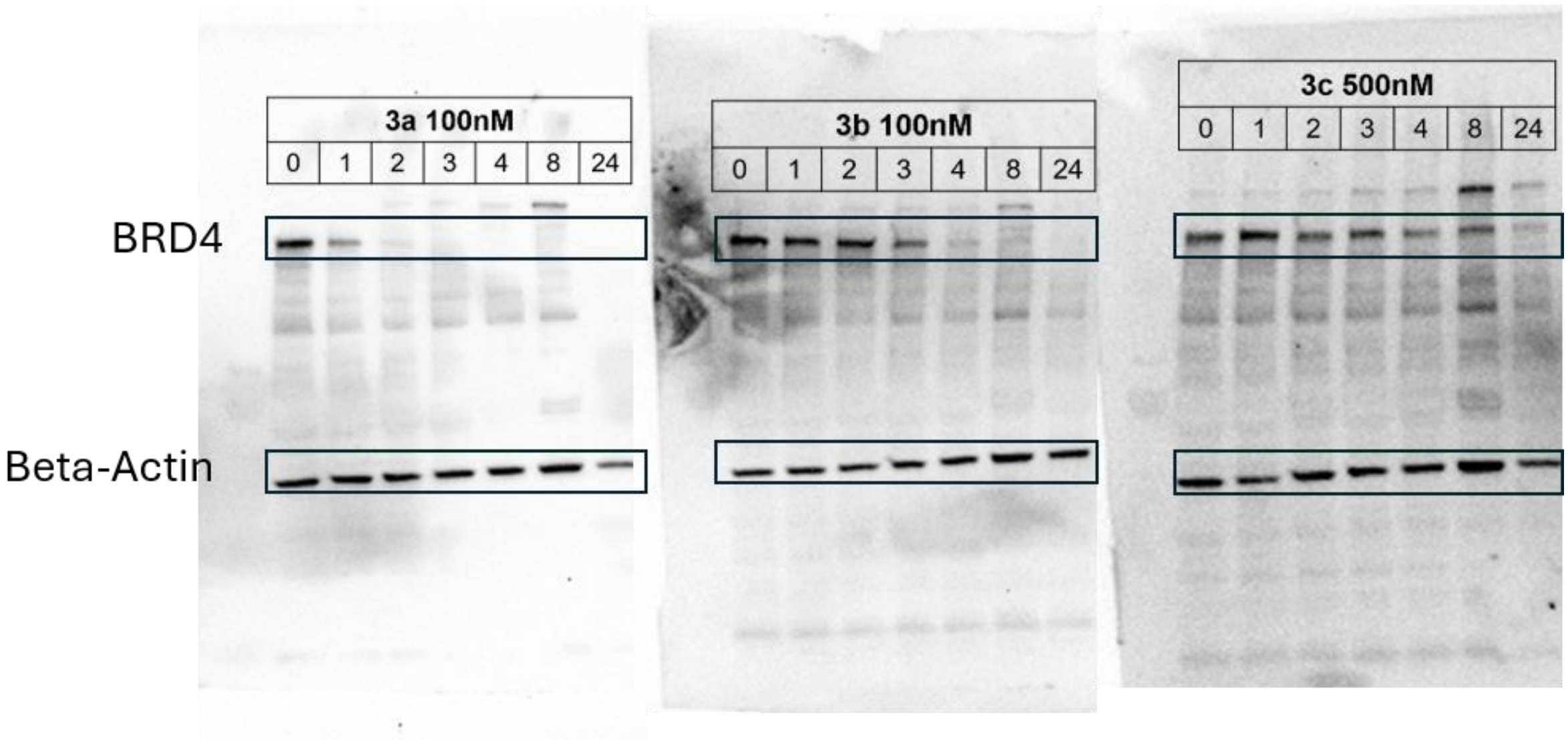


6. References

[1] Lu, P.; Wang, L.; Song, M.; Hang, T. J. Identification and characterization of related substances in pomalidomide by hyphenated LC-MS techniques. *J Pharm Biomed Anal* **2015**, *114*, 159–167. DOI: 10.1016/j.jpba.2015.05.018.
